# Unexpected Dual Function of Plant YUCCA Enzymes Links Chlorophyll Catabolism to Auxin Homeostasis

**DOI:** 10.1002/anie.202525568

**Published:** 2026-01-21

**Authors:** Sina Rütschlin, Lei Zhang, Cornelia A. Karg, Michael Zwerger, Johanna M. Gostner, Simone Moser, Robin Teufel

**Affiliations:** ^1^ Department of Pharmaceutical Sciences, Pharmaceutical Biology University of Basel Basel 4056 Switzerland; ^2^ Department of Pharmacognosy Institute of Pharmacy University of Innsbruck Innsbruck A‐6020 Austria; ^3^ Institute of Medical Biochemistry Medical University of Innsbruck Innsbruck A‐6020 Austria; ^4^ Core Facility Metabolomics II, Institute of Medical Biochemistry Medical University of Innsbruck Innsbruck A‐6020 Austria

**Keywords:** Auxin, Baeyer–Villiger monooxygenase, Chlorophyll catabolism, Hormones, Phyllobilins

## Abstract

Chlorophyll (Chl) metabolism is pivotal to both photosynthesis and plant senescence and represents one of the most fundamental biological processes on Earth with an estimated annual turnover of 1 billion tons. During Chl degradation, only early catabolites and corresponding enzymes are well characterized, whereas for late‐stage degradation products it remains often unclear if their formation involves specific enzymes. Here, we report that the ubiquitous YUCCA10 enzymes from the YUCCA flavin‐containing monooxygenase (FMOs) family in land plants, normally implicated in the biosynthesis of indole‐3‐acetic acid (IAA) as the primary form of auxin, surprisingly catalyze the production of several predominant Chl catabolites via mechanistically distinct Baeyer–Villiger oxidation and subsequent hydrolytic γ‐lactam‐forming deformylation reactions. These historically postulated but hitherto undiscovered Chl degradation steps on several high molecular weight chl catabolites were verified for YUCCA10 from *Vitis vinifera* and *Coffea arabica*, while YUCCA10 from *Arabidopsis thaliana* lacked this activity. In contrast, all three homologs were able to catalyze the rate‐limiting key step in IAA biosynthesis, akin to other YUCCA enzymes. Interestingly, Chl catabolites at physiological concentrations impaired IAA formation by YUCCA10 in vitro, suggesting a key role in leaf senescence through enzymatic feedback regulation of auxin levels.

## Graphical abstract

YUCCA enzymes are well known to catalyze the main step of auxin biosynthesis in plants. Here, a hitherto undescribed dual function was discovered, revealing that some YUCCAs also act in chlorophyll degradation. In vitro feedback regulation furthermore suggests a link between chlorophyll degradation and hormone homeostasis and a physiological role of accumulating chlorophyll catabolites for plant senescence.

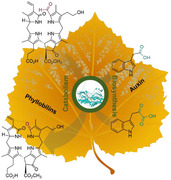

## Introduction

**Scheme 1:**
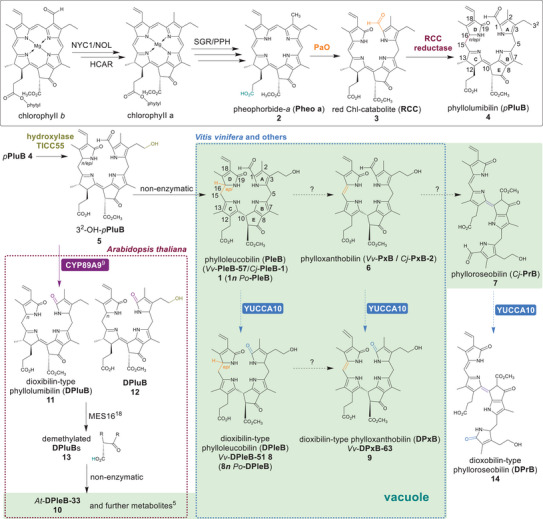
Overview on early and late chlorophyll degradation steps in plants, in particular *Arabidopsis thaliana* and *Vitis vinifera*. Dashed arrows indicate unknown enzymatic transformations, the blue dashed lines indicate enzymatic reactions elucidated in this work. Important enzymatic modifications are highlighted in colour. The absolute configuration at C16 remains undetermined. The “*n*” assignment for non‐fluorescent Chl catabolites (NCCs/ PleBs) originates from primary fluorescent Chl catabolites (*p*FCC / *p*PluB) and “*epi*” assignment for PleBs originates from ***epi***‐*p*FCC/ *p*PluB. **Black box**: Well‐investigated early steps of chlorophyll degradation in plants leading to the formation of *p*PluB **4** that is further converted to 3^2^‐OH‐*p*PluB **5**. ***A. thaliana* box** (red): Branching of the PaO/phyllobilin pathway in *A. thaliana* occurs at the stage of *p*PluBs. In the simplified structure for **13**, R denotes the identical remaining structural components of **11** and **12**. ***V. vinifera* box**(blue): Chl degradation pathway leading to yellow Chl catabolites (YCCs / PxBs) as final catabolites as well as deformylated DPleB‐51 **8** and DPxB‐63 **9** found, for example, in leaf extracts.^[^ ^16^ ^]^ In this manuscript, the C16 *n*‐epimer, PleB **1*n***, was used for various assays to yield DPleB **8*n***. Note that the pink Chl catabolite (PiCC / PrB) **7** is not found in *V. vinifera* and DPrB **14** has so far not been described as natural product but can be produced by YUCCA10 in vitro. **Vacuole box** (green background): Part of the Chl degradation pathway, which presumably takes place in the plant vacuole.

In autumn or under pathogen invasion, the controlled degradation of the green pigment chlorophyll (Chl) leads to the abscission of leaves and other plant parts.^[^ ^1^ ^]^ Notably, antioxidative properties, anti‐inflammatory or anti‐cancer activities have been reported for these Chl catabolites, referred to as the phyllobilins, suggesting that they are likely not mere degradation products but serve biological roles. This is further supported by recent studies showing the direct inhibition of actin dynamics in human cells by some of these compounds.^[^ ^2^ ^]^ The early steps of this degradation pathway are conserved, enzymatically driven and well regulated, resulting in the formation of the frequently observed “non‐fluorescent” Chl catabolites (NCCs) **1** (lacking an extended π‐electron system due to the missing C10 double bond), which are nowadays referred to as phylloleucobilins (PleBs)^[^ ^3^, ^4^ ^]^ (Scheme 1; for compound nomenclature see methods section and ref.[5]). The first catabolic steps are the dephytylation and dechelation of Mg^2+^ of Chl a by the enzymes pheophytinase (PPH) and Mg‐dechelatase (SGR) (Scheme 1, black box). Subsequently, the highly phototoxic pheophorbide‐*a* (Pheo *a*) **2** is transformed into the red Chl catabolite (RCC) **3** by the key enzyme pheophorbide‐*a* oxygenase (PaO) that oxidatively cleaves the bond between rings D and A, transforming the bridging carbon into an aldehyde group on ring A. RCC **3** is then reduced at the C15 ═ C16 double bond by one of two types of stereospecific RCC reductases to the primary fluorescent Chl catabolite phyllolumibilin *p*PluB **4** (formerly known as *p*FCC) or its C16 epi isomer, which is further modified at position C3^2^ by hydroxylase TIC55 (located at the outer/inner envelope membrane of chloroplasts) to 3^2^‐OH‐*p*PluB **5** (Scheme 1).^[^ ^6^ ^]^ From here on, several major and minor downstream pathways lead to the formation of species‐specific Chl catabolites,^[^ ^3^ ^]^ for example, PleB **1**, reported for *V. vinifera* (grapevine) and *Cercidiphyllum japonicum* (katsura tree). This compound arises from an acid‐catalyzed non‐enzymatic isomerization of **5** likely taking place in the plant vacuole,^[^ ^7^ ^]^ the proposed major final storage place of PleBs,^[^ ^8^, ^9^ ^]^ which were once considered the exclusive final products of Chl degradation. However, more recent studies have identified two additional classes of molecules commonly found in senescent leaves, such as those of the katsura tree^[^ ^10^ ^]^: yellow Chl catabolites (YCCs) and pink Chl catabolites (PiCCs) now referred to as the phylloxanthobilins (PxBs, **6**) and phylloroseobilins (PrBs, **7**), respectively. PxBs exhibit potent antioxidant and anticancer properties^[^ ^11^, ^12^, ^13^ ^]^ and feature a C15═C16 double bond. Their oxidation products, PrBs, have the C10═C11 double bond restored, increasing their reactivity and potential toxicity. This likely promotes further degradation, consistent with their low abundance in leaves (Scheme 1, vacuole box).^[^ ^14^ ^]^ The enzymes mediating the late‐stage conversions from PleBs to PxBs and PrBs remain unknown, despite observed activities.^[^ ^15^ ^]^

Aside from **1**, **6,** and **7**, a second branch of Chl catabolites, the dioxobilins (**8**, **9**, and **10**), derives from the PaO/phyllobilin pathway.^[^ ^17^ ^]^ These compounds can be structurally described as deformylated phyllobilins (e.g., dioxobilin‐type PleBs = DPleBs) and commonly accompany PleBs in senescent leaves, as shown for numerous unrelated plants ranging from *V. vinifera*, Norway maple (*Acer platanoides*), savoy cabbage (*Brassica oleracea* var. *sabauda*) to the model organism *Arabidopsis thaliana*.^[^ ^9^, ^12^, ^16^, ^17^ ^]^ DPleBs are structurally similar to human bilin and biliverdin and it is hypothesized that their close resemblance coincides with related physiological properties in the cell^[^ ^12^ ^]^ though their functions remain unknown aside from antioxidative properties.^[^ ^2^ ^]^ In *A. thaliana*, the cytochrome P450 enzyme CYP89A9 that is localized to the endoplasmatic reticulum (ER) first catalyzes deformylation of the *p*PluBs **4** and **5** to produce DPluBs **11** and **12**, respectively (Scheme 1, *A. thaliana* box).^[^ ^9^ ^]^ The subsequent enzymatic conversion of the DPluBs **11** and **12** by methylesterase MES16 affords distinct demethylated catabolites summarized as **13**, which then nonenzymatically isomerize to the corresponding dioxobilins such as *At*‐DPleB **10**.^[^ ^3^, ^5^, ^18^ ^]^ It is important to note that CYP89A9 acts specifically on the *p*PluBs **4** and **5**, but is not responsible for the deformylation of PleB **1**, respectively. The distinct branching of the PaO/phylloleucobilin pathway in *A. thaliana* is thus determined by CYP89A9 on the level of PluBs **4** and **5** and occurs before these compounds enter the vacuole. It appears that other plants like *V. vinifera* lack the methylesterase functionality and a significant gap in our understanding of Chl degradation in these plants is thus the missing enzyme(s) responsible for the deformylation of **1** and **6**.

Plant senescence including Chl degradation and the biosynthesis of growth‐promoting hormones, for example, by the major biosynthetic pathway of auxin indole‐3‐acetic acid (IAA **16**) from tryptophan via indole‐3‐pyruvic acid (IPA **15**),^[^ ^19^ ^]^ are evidently opposing processes, yet it remains unclear how they are exactly co‐regulated.^[^ ^20^ ^]^ Aside from induction of plant growth, **16** is crucial for regulation of nearly all plant developmental stages including fruit ripening and its presence is usually tightly regulated by a complex resolution of transient concentrations and gradients in various tissues and organs of the plant.^[^ ^21^ ^]^ Recently, the verification of **16** in vacuoles as well as of the first vacuolar auxin transporter WAT1 in *A. thaliana* suggest a key role of vacuoles in auxin homeostasis in the plant cell.^[^ ^22^ ^]^ The biosynthesis of **16** is initiated by the ubiquitously expressed cytoplasmatic tryptophan transaminase converting tryptophan (Trp) into **15**, which is then oxidatively decarboxylated into **16** in the second and last enzymatic step by YUCCA enzymes (Figure 3a).^[^ ^23^, ^24^, ^25^, ^26^ ^]^ YUCCAs comprise a total of 11 members and belong to the flavin‐containing monooxygenase (FMO) subgroup of the group B flavoprotein monooxygenases (FPMOs), which feature a Rossmann fold and utilize FAD and NAD(P)H as cofactors.^[^ ^27^, ^28^, ^29^ ^]^ To date, foremost YUCCA6 from *A. thaliana* was investigated in detail and proposed to employ a nucleophilic flavin‐C4a‐peroxide intermediate (Fl_C4aOO_) for the oxidative decarboxylation of **15**.^[^ ^23^ ^]^ FMOs including the YUCCAs are likely ancestral enzymes and presumably already present in the last universal common ancestor (LUCA).^[^ ^30^ ^]^ YUCCAs also share a close relationship by sequence similarity network with human liver FMOs, which exhibit broad substrate promiscuity for detoxification of xenobiotics^[^ ^23^ ^]^ due to an accessible and exposed active site.^[^ ^31^ ^]^ For **16** biosynthesis in plants, the expression of the different YUCCAs is tissue specific (in contrast to Trp transaminase) and the corresponding homologs can either be cytosolic or attached to the ER in *A. thaliana*.^[^ ^19^, ^32^, ^33^ ^]^ Furthermore, it was shown that *A. thaliana* can produce different splicing variants of YUCCA4, which localize in different compartments of the same organ.^[^ ^34^ ^]^ One of these enzymes, for example, was restricted to the ER in flowers, suggesting specialized roles of YUCCA variants in different plant parts. The crucial role of YUCCAs in auxin biosynthesis is underscored, for example, by the observed increased longevity and delayed senescence in *A. thaliana* upon overexpression of YUCCA6.^[^ ^20^, ^22^, ^35^, ^36^ ^]^ Importantly, YUCCA enzymes appear to operate isofunctionally and only multiple deletion mutants therefore show significant developmental defects.^[^ ^37^ ^]^ Recently, a dual role has been described for YUCCA6 which contains a thioredoxin domain in its active site that reduces reactive oxygen species (ROS), enhances stress tolerance and delays leaf senescence in *A. thaliana* plants overexpressing this enzyme, independent of its FMO activity and ability to produce **16**.^[^ ^20^, ^38^, ^39^ ^]^

In this work, we uncover a dual role for YUCCA10 in the phylogenetically distant plants *Vitis vinifera* and *Coffea arabica*. Beyond its established function in IAA **16** biosynthesis, we surprisingly identified phyllobilins **1**, **6**, and **7** as additional substrates for YUCCA10, which are converted in a Baeyer–Villiger monooxygenase (BVMO)‐type reaction, followed by hydrolytic deformylation to species‐specific high molecular weight compounds (**8**, **9**, and **14**) – a reaction similar to the CYP89A9‐catalyzed formation of **11** and **12** in *A. thaliana*. Intriguingly, in vitro assays with YUCCA10 suggest a feedback inhibition mechanism for regulating **16** levels and provide a molecular basis for decreasing auxin levels in aging leaves.

## Results and Discussion

### Identification of *Vitis vinifera* (*Vv*)YUCCA10 as Chl Catabolic Enzyme

In an effort to identify missing enzymes in the ChI degradation pathway responsible for the processing of late‐stage Chl catabolites such as **1**, **6**, **8**, or **9,** and with evidence for those activities in plant lysates,^[^ ^15^ ^]^ we synthesized an **8**‐Biotin‐PEG_7_ probe to perform affinity chromatography with a grapevine (*Vitis vinifera*) leaf lysate (Figure S1A). *V. vinifera* was chosen as model system, because ChI degradation and catabolites are well investigated.^[^ ^16^ ^]^ In addition, an annotated genome (genome assembly ASM3070453v1; NCBI data base), reference proteome (UP000009183; Uniprot data base) as well as processed transcriptomic data (co‐expression database ATTED‐II^[^ ^40^ ^]^) of *V. vinifera* are available. In short, for the synthesis of the probe, isolated *Vv*‐DPleB‐51 **8** (former *Vv*‐DNCC‐51) was used as bait and coupled via hydroxybenzotriazole (HOBt) and 1‐ethyl‐3‐(3‐dimethylaminopropyl)carbodiimide (EDCl) activation to biotin‐polyethylene glycol (PEG)_7_‐amine and purified by semi‐preparative HPLC. *V. vinifera* leaf lysate was prepared by grinding frozen aging yellow–green leaves in ice‐cold phosphate buffer, followed by lysis and centrifugation. In the succeeding affinity chromatography, the lysate‐derived proteins binding to the probe were analyzed by a proteomics approach and shown to comprise well‐known enzymes involved in Chl catabolism such as the Chlorophyll(ide) b reductase (NOL), TICC55, and PaO. We then scrutinized the pulled‐down proteins for novel enzyme candidates, focussing on annotated oxidoreductases such as cytochrome P450s or flavoproteins as plausible enzyme candidates for late‐stage ChI catabolism (Scheme 1, vacuole box). Furthermore, we complemented the search by feeding ATTED‐II^[^ ^40^ ^]^ with all enzymes known to be involved in the early steps of Chl degradation in *V. vinifera* (Table S1). In case the homologs from *V. vinifera* were not already described in the literature, we conducted manual pBLAST searches and curated them from the genome. The co‐expression network generated (retrieved in January, 2025) was then compared with the hit list from the pull‐down analysis (Figure S2 and S3). The comparison resulted in a single hit for a flavin‐dependent enzyme (F6HQ23), which was detected with around the same quantitative value (normalized NASF) than the other Chl degradation enzymes in the LC‐MS analysis and was further co‐expressed with PPH, PaO, and TIC55 in data retrieved from ATTED‐II. In NCBI, the corresponding gene sequence was predicted to encode a variant of an indole‐3‐pyruvate monooxygenase YUCCA10 (LOC100245859, Uniprot: F6HQ23) involved in auxin biosynthesis.^[^ ^20^, ^23^, ^26^, ^38^, ^41^, ^42^ ^]^ To further investigate this, *Vv*YUCCA10 was subsequently heterologously expressed as maltose binding protein (MBP)‐tagged enzyme in *E. coli* BL21 and successfully purified by affinity chromatography as yellow protein with a tightly bound FAD cofactor (Figure S4). A PDB search of the protein sequence resulted in low sequence similarity (<30%) hits with bacterial FMOs, in particular from *Streptomycetes* as well as psychrophilic bacteria like *Janthinobacterium svalbardensis* and, interestingly, reconstructed ancestral mammalian FMOs. Studies on the YUCCA family, for which a protein structure is lacking, have been mostly conducted with *A. thaliana* enzymes^[^ ^23^, ^25^, ^32^, ^41^, ^42^ ^]^ and the localization of the predicted functional homolog *At*YUCCA10 is presumably cytosolic.^[^ ^32^ ^]^ Although *Vv*YUCCA10 showed closest relationship to *At*YUCCA10 in a phylogenetic tree generated with all 11 variants of YUCCA from *A*. *thaliana* (Table S2, Figure S5), the TMHMM tool predicted a transmembrane helix for *Vv*YUCCA10 (Table S3) for which vacuolar localization has been confirmed in berry flesh.^[^ ^43^ ^]^

### *Vv*YUCCA10 Acts as FPMO and Deformylates PleB to DPleB

**Figure 1:**
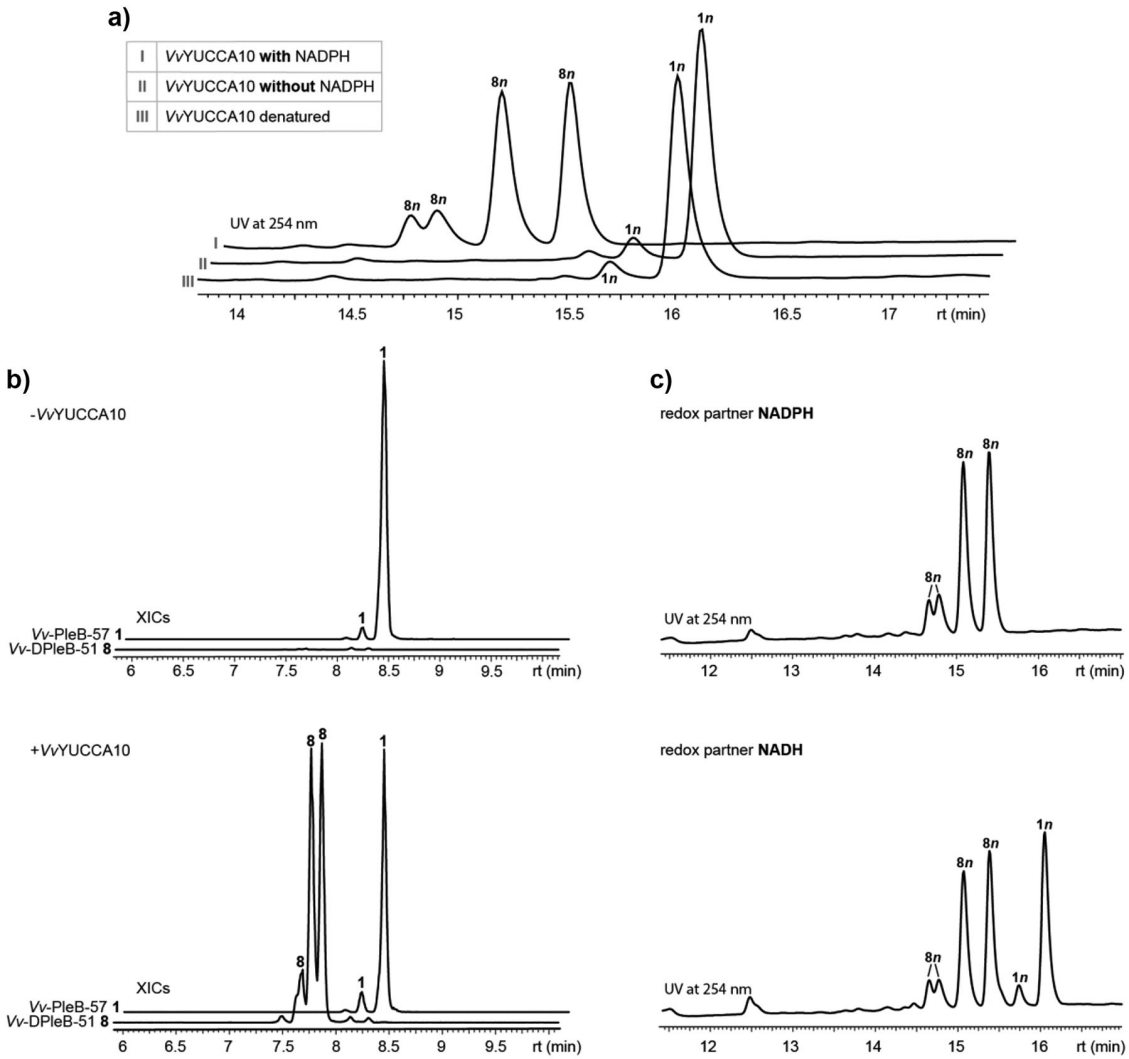
FAD and NADPH‐dependent conversion of *Vv*‐PleB‐57 **1** and *Po*‐PleB **1*n*** to *Vv*‐DPleB‐51 **8** and *Po*‐DPleB **8*n*** catalyzed by *Vv*YUCCA10‐MBP. All peaks indicated for **8** and **8*n*** share the same mass and fragments and are assumed stereoisomers. The epimerization of **1** and **1*n*** is commonly observed with acetonitrile as a LC solvent. The reaction to **8**/**8*n*** creates a new chiral center that leads to further isomerization of the two epimers, resulting in four peaks. **a)** HPLC‐DAD chromatograms at 254 nm of the enzymatic *Vv*YUCCA10‐MBP assays with *Po*‐PleB **1*n*** producing **8*n*** in presence of NADPH; controls are shown that contain denatured enzyme or lack NADPH. **b)** XIC traces of HR‐MS measurements for *Vv*YUCCA10‐MBP assays with *Vv*‐PleB‐57 **1** (*m/z* = 645.29249 [M + H]^+^) producing **8** (*m/z* = 633.29249 [M + H]^+^) (bottom) and control reaction lacking enzyme (top). The *y*‐axis scale was equalized by setting the highest peak to 100% for both measurements. **c)** Comparison of *Po*‐DPleB **8*n*** production with NADPH (top) versus NADH (bottom) as redox partner.

To establish an activity assay, *Po‐*PleB **1*n***, the “*n*” epimer of *Vv*‐PleB‐57 **1**, which also has a characteristic absorption maximum at 315 nm due to the formyl group, was extracted from plane tree leaves (*Platanus occidentalis*) due to limited access to the *Vitis* substrate (Figure S1B, Table S4).^[^ ^11^ ^]^ Gratifyingly, **1*n*** was also readily converted by purified *Vv*YUCCA10‐MBP in the presence of NADPH (NADH was also accepted but resulted in lower product formation) into four new peaks that lacked the distinct UV absorption at 315 nm, while controls with denatured *Vv*YUCCA10‐MBP or without NADPH showed no substrate turnover (Figure 1). HR‐Orbitrap analysis of these enzymatic products revealed a pseudo molecular ion with *m/z* = 633.292 [M + H]^+^, indicating a molecular formula of C_34_H_40_N_4_O_8_ and a formal loss of CH_2_ compared to the substrate (Figures S6 and S7). Substrate **1*n*** showed typical epimerization behavior in LC solvents containing acetonitrile,^[^ ^44^ ^]^ forming a minor and a major peak and the observed enzymatic product peaks therefore correspond to two sets of stereoisomers originating from a new chiral center at C4 of **8*n*** at ring A. Comparison to an isolated standard by UV‐vis, HR‐MS, and MS^2^ fragmentation confirmed that PleB **1** indeed had been deformylated to DPleB **8** (Figures S7 and S8). To confirm the expected FPMO functionality, the reaction was then performed under ^18^O_2_ atmosphere, which resulted in the incorporation of one ^18^O atom into *Po*‐DPleB **8*n*** (Figure S9). Moreover, MS^2^ fragmentation corroborated the newly formed keto group of ring A as site of oxygen incorporation (Figure S10). The discovered *Vv*YUCCA10 functionality raised the question if *V. vinifera* also possesses functional homologs of CYP89A9, which catalyse similar deformylations in *A. thaliana*. Blast search with the sequence of CYP89A9 against the *V. vinifera* proteome resulted in 44 hits with >40% sequence identity, indicating 44 candidates of the same CYP family.^[^ ^45^ ^]^ One of these hits shared a sequence similarity greater 55% (indicating the same subfamily), but was absent in the co‐expression networks created with ATTED fed with all known Chl catabolic enzymes of the early steps. As none of the 44 candidates was pulled down with the *Vv*‐DPleB‐51‐biotin‐probe in the grapevine lysate, we assume there is no functional equivalent to CYP89A9 present in *Vitis* based on our approach. However, as a glycosylated version of deformylated PluBs, *Vv*‐DPluB‐53, is present in *Vitis* extracts, another undiscovered enzymatic transformation present on the PluB level may be required to produce deformylated PluBs, which may possibly involve such a CYP89A9 homologue.^[^ ^16^ ^]^

### Confirmation and Proposed Mechanism of the *Vv*YUCCA10‐catalyzed Formation of Auxin IAA and Chlorophyll Catabolite DPleB

**Figure 2:**
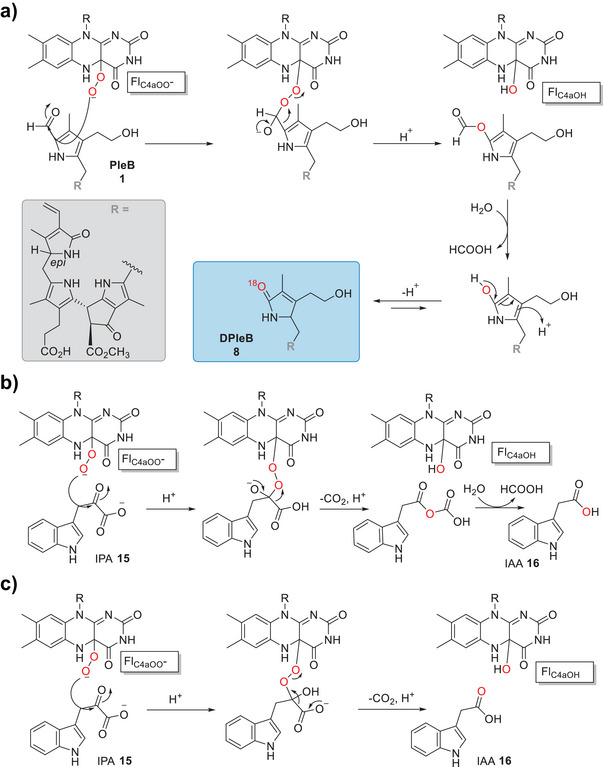
Postulated reaction mechanism for YUCCA enzymes. The Fl_C4aOO_ intermediate is formed by reduction of the FAD cofactor with NADPH and subsequent reaction with O_2_ (not shown). a) Proposed *Vv*YUCCA10‐catalyzed Baeyer–Villiger oxygenation of substrate **1** and subsequent hydrolytic deformylation and tautomerization leading to formation of product **8** (highlighted in blue). ^18^O_2_ Isotope labelling experiments support the proposed mechanism and the incorporated oxygen atom derived from ^18^O_2_ is shown in red. b) Revised BVMO‐type YUCCA mechanism for the transformation of **15** into **16**, analogous to the conversion **1** into **8**. c) Previously postulated mechanism for YUCCA6 from *A. thaliana*, involving an oxidative decarboxylation of **15** into **16**.^[^ ^23^ ^].^

**Figure 3:**
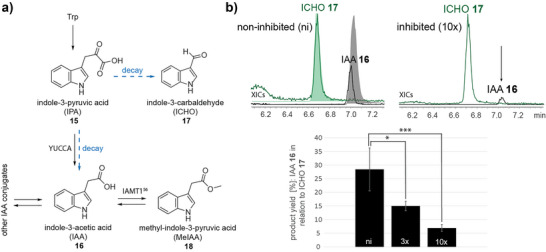
Feedback inhibition of the *Vv*YUCCA10‐mediated formation of IAA **16** by *Vv*‐DPleB‐51 **8**. a) Overview on **16** biosynthesis in plants involving YUCCAs. The substrate IPA **15** decays in buffer into ICHO **17** and **16**. Compound **16** can be partially further converted in the cell, for example, into methyl‐indole‐3‐pyruvic acid (MeIAA) **18** as shown for *A. thaliana* and poplar (*Populus* spp.).^[^ ^55^ ^]^ b) *Vv*YUCCA10‐MBP pre‐incubation with *Vv*‐DPleB‐51 **8** impairs conversion of **15** into **16**. **Left**: XIC traces of **17** and **16** after *Vv*YUCCA10‐MBP was incubated with IPA **15**; non‐inhibited (ni). Additionally, the respective XICs for commercial standards of **16** and **17** are shown as their shadows. **Right**: XIC traces of **17** and **16** after *Vv*YUCCA10‐MBP was pre‐incubated with a tenfold (10×) excess of **8** compared to added substrate **15**; inhibited. The *y*‐axis of the chromatograms was set to equal. The bar chart shows the mean product yield of in vitro production of **16** in relation to **17** in the absence (ni) and the presence of 3× and 10× excess of **8** over substrate **15** for *n* = 3. The product yields were determined by area under the curves (AUC) of XIC traces generated for **16** and **17** and subsequent calculation of product formation in percent as ratio of **16** to overall **16** and **17** formation. The error bars are shown as calculated standard deviations from the mean of 3 replicates. Statistics were assessed by a two‐sample *t*‐test with equal variance assumption for triplicates, resulting in statistically significant 38% (3×, *P* = 0.012) and 76% (10×, *P* = 0.00083) lowered product formation due to DPleB **8** inhibition. Values that were significantly different were marked by bars with ****: *P* < 0.0001.***: *P* < 0.0001–0.001, **:*P* < 0.001–0.01 and *:*P* < 0.01–0.05.

The well‐investigated role of YUCCAs described in literature raised the question whether *Vv*YUCCA10 would also act as an auxin biosynthetic enzyme (Figure 3a). Indeed, when *Vv*YUCCA10‐MBP was incubated with NADPH in vitro, it readily converted **15** into **16** (Figure S11). Next to the **16** peak with a pseudo molecular ion of *m*/*z* = 176.0706 [M + H]^+^ and characteristic UV absorption at 274, 282, and 288 nm, another major peak was detected by HR‐MS with a *m/z* = 146.0600 [M + H]^+^, which was identified as indole‐3‐carbaldehyde (ICHO; **17**). Compound **17** is frequently found as degradation product of highly instable **15** in aqueous buffer solution and has a characteristic absorption at 297 nm.^[^ ^46^ ^]^ Compound **16** is also the degradation product of **15**, though **16** levels were shown to increase significantly in the presence of the enzyme, while control reactions with denatured *Vv*YUCCA10‐MBP or without NADPH did not result in a detectable increase of **16** compared to **17** (Figure S12). Notably, our findings on the YUCCA10‐mediated turnover of Chl catabolite **1** suggests that the previously postulated mechanism for **16** production by YUCCAs may require revision. We propose a unifying reaction mechanism for both types of substrates that proceeds via initial flavin reduction by NADPH and subsequent reaction with O_2_ to the Fl_C4aOO_ species. This is followed by a BVMO mechanism involving the nucleophilic attack of the anionic Fl_C4aOO_ on the carbonyl functionalities of **1** (Figure 2a) or **15** (Figure 2b) and formation of the corresponding Criegee intermediates, facilitating the corresponding carbon bond migrations. The resulting formate ester intermediates could not be observed and are likely rapidly hydrolyzed, leading to the final products **8** or **16**, respectively (Figures 2a,b). Rather than the previously postulated oxidative α‐ketoacid decarboxylation (typically a hallmark reaction of thiamine diphosphate (TPP)‐dependent enzymes) (Figure 2c),^[^ ^23^ ^]^ YUCCAs may thus catalyze *bona fide* Baeyer–Villiger oxygenations analogous to many closely related group B FPMOs, which have significant potential for industrial applications.^[^ ^30^, ^47^, ^48^ ^]^

So far, plant FMOs have only been reported to transform small compounds and most of the authentic biological substrates remain unknown. To our knowledge, *Vv*YUCCA10 is the first plant FMOs that can act on high molecular weight substrates, thereby closing substantial gaps in our understanding of both FMO substrate specificity and catalysis as well as Chl catabolism. Deformylation by a flavoenzyme is rare in nature and to the best of our knowledge has only been described for fungal FMOs converting aliphatic aldehydes^[^ ^47^ ^]^ rather than the complex Chl catabolites processed by YUCCA10.

### Feedback Inhibition by Deformylated Chl Catabolites Leads to Decreased IAA Levels

To investigate physiological levels of auxin and Chl catabolites in different plants, we investigated ChI catabolite and auxin levels in *V. vinifera* leaves. Additionally, *A. thaliana* siliques at different stages of senescence were examined, where YUCCA10 was reported to be highly expressed.^[^ ^42^ ^]^ In literature, concentrations of Chl catabolites and auxin differ depending on the investigated plant part and the detection method used, but generally, Chl catabolite levels appear to be substantially higher than auxin levels per fresh weight gram (gfw) of plant material. Levels of **16** are usually detected in a double digit pg/gfw range, whereas physiological levels of ChI catabolites have been reported to be in the triple digit ng/gfw and even µg/gfw level, thus exceeding **16** levels at least 1000‐fold.^[^ ^13^, ^20^, ^26^, ^37^, ^49^, ^50^, ^51^, ^52^, ^53^ ^]^ When we extracted yellow–green grapevine leaves used for affinity chromatography, we could detect HR‐MS masses and fragments for **8** (*m/z* = 633.290 [M + H]^+^) and **9** (*m/z* = 631.275 [M + H]^+^) as reported in the literature (Figures S13 and S14).^[^ ^16^ ^]^ Compounds **16** or **15** were not found under the applied HR‐MS conditions with a detection limit of 10 pg/mL for **16** spiked into the vine leaf lysate (Figure S15). Similarly, **16** and its precursors were not detectable in *A. thaliana* siliques in contrast to the Chl catabolites and the Trp degradation product kynurenic acid (KynA) of the kynurenine pathway, which increased in aging siliques (Figures S16). Therefore, we assume physiological levels of Chl catabolites in yellow–green grapevine leaves are indeed vastly higher than free **16** or **15** levels, fully consistent with previous reports. Due to the confirmed dual catalytic role of *Vv*YUCCA10, we concluded that *Vv*YUCCA10 might play an important role in auxin homeostasis in the plant cells via feedback regulation during leaf senescence. To test this hypothesis, we incubated *Vv*YUCCA10 with moderate threefold (3×) and tenfold (10×) excess of *Vv*‐DPleB‐51 **8** over **15**. After an incubation time of 20 min, the formation of **16** was impaired by 38% for 3× and 76% for 10× in contrast to the enzyme assay lacking **8** (Figure 3b). In contrast, when *Vv*YUCCA10 was incubated with a tenfold excess of **16** over PleB **1*n***, formation of product **8*n*** was not impaired (Figure S17). These results indicate that large excess of Chl catabolites under physiological conditions may almost entirely suppress auxin biosynthesis by YUCCA10, implying a role in delaying fruit ripening and abscission by production of IAA **16**.^[^ ^35^, ^36^, ^54^ ^]^

Beyond the Chl–auxin connection, detected increasing levels of kynurenic acid (KynA) in *A. thaliana* siliques could indicate a second, self‐reinforcing feedback loop that promotes senescence progression. Kynurenine (Kyn)—a precursor of KynA—was previously shown to decrease auxin biosynthesis in roots of *A. thaliana*.^[^ ^56^ ^]^ The key enzymes in the first step of the major IPA pathway for auxin biosynthesis from Trp, tryptophan aminotransferase of Arabidopsis 1 (TAA1/TARs) and its homologs, were identified as molecular targets of Kyn. As auxin biosynthesis via the TAA1/TAR–YUCCA (IPA) pathway diminishes during senescence, Trp may be increasingly diverted through the Kyn branch, leading to the accumulation of KynA. Elevated Kyn metabolites in turn further suppress auxin biosynthesis by inhibiting TAA1/TAR enzymes, thereby establishing a metabolic feedback circuit that reinforces senescence‐associated hormonal decline.

### Substrate Tolerance Expands to Other Chl Catabolites

**Figure 4:**
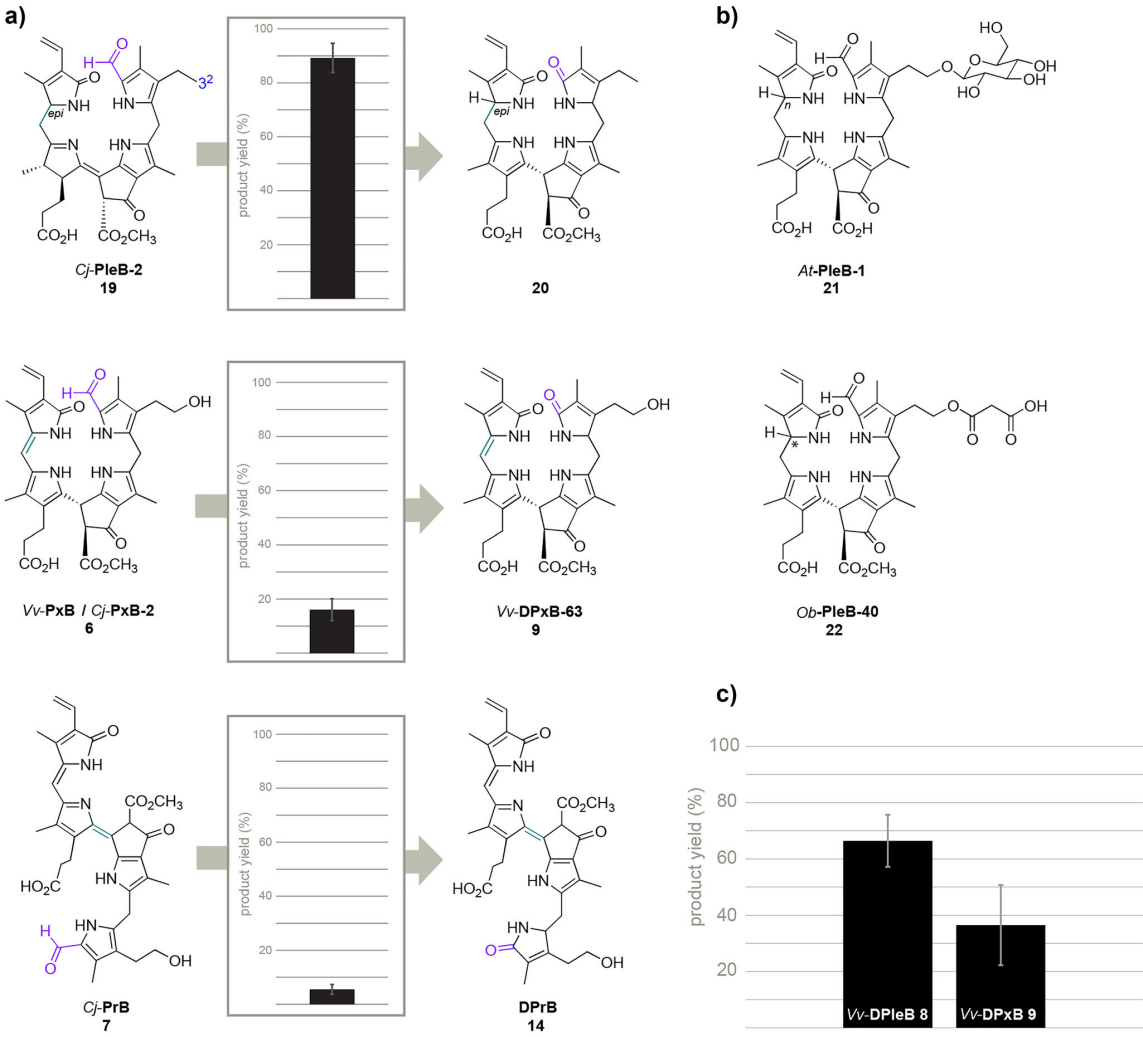
*Vv*YUCCA10 substrate tolerance for deformylation reaction and competition experiments. a) Deformylation of Chl catabolites **19**, **6**, and **7** after 20 min incubation with *Vv*YUCCA10 and their product yields. The product yields were determined by area under the curves (AUC) of UV‐HPLC traces of substrate and product peaks and subsequent calculation of product formation in percent as ratio of product to substrate AUC. The error bars are shown as calculated standard deviations from the mean of 3 replicates. b) Structures of Chl catabolites *At*‐PleB‐1 **21** and *Ob*‐PleB‐40 **22** from *Arabidopsis thaliana* (*At*) and *Ocimum basilicum* (*Ob*).* = stereochemistry not determined. c) Product yields for deformylated Chl catabolites observed for a 1:1 incubation of *Vv*‐PleB‐57 **1** and *Vv*‐PxB **6** after 20 min incubation time with *Vv*YUCCA10. Product yields and error bars were determined as described for a).

Next, the substrate tolerance of *Vv*YUCCA10 was investigated in enzymatic assays using previously characterized Chl catabolites (see Table S4 for an overview) as substrates, showing that non‐hydroxylated *Cj*‐PleB‐2 **19**, *Vv*‐PxB‐57 **6** and *Cj*‐PrB **7** were also readily deformylated into **20**, *Vv*‐DPxB‐63 **9** and DPrB **14**, respectively (Figures 4a and S18–S23). Compound **9** is a common constituent of the *V. vinifera* lysate of yellow‐green extracts (Figure S13).^[^ ^16^ ^]^ *Cj*‐PrB **7** was more slowly converted into DPrB **14** by the enzyme than **6** and **9** resulting in lower product yields after 20‐min incubation time. However, neither **7** nor **14** have so far been described in grapevine leaf lysate and were also not detected in our samples.^[^ ^16^ ^]^ Compound **7** occurs naturally in senescent leaves of the katsura tree (*Cercidiphyllum japonicum*), whereas DPrB **14** has been produced only via chemical synthesis to date.^[^ ^57^, ^58^ ^]^ *Vv*YUCCA10 thus enables the first enzymatic route to access DPrBs. Other related ChI catabolites from savoy cabbage (*Brassica oleracea var sabauda*) / *A. thaliana Bos*‐PleB‐1/*At*‐PleB‐1 **21** and basil (*Ocimum basilicum*) *Ob*‐PleB‐40 **22**, which were either glycosylated or malonylated at position 3^2^, were not or only in traces deformylated by *Vv*YUCCA10 (Figure 4b), suggesting a restricted substrate tolerance toward 3^2^‐substituted Chl catabolites. Moreover, neither cyclohexanone (a common BVMO substrate) nor phenylpyruvate (a confirmed substrate of YUCCA6 from *A. thaliana*) were converted by *Vv*YUCCA10 (Figure S24).^[^ ^23^, ^28^ ^]^

It is noteworthy that several attempts to determine reliable enzyme kinetics and K_M_ values for the conversion for PleB **1** by *Vv*YUCCA10 were unsuccessful, presumably because purified and tag‐cleaved enzyme was largely aggregated when analyzed by size exclusion chromatography (SEC) and furthermore due to inhibitory effects, likely caused by both the substrate and the product (Figure S25). Still, we concluded that PrB **7** is most likely a non‐native substrate, whereas *Vv*‐PleB‐57 **1** and *Vv*‐PxB/*Cj*‐PxB‐2 **6** seem plausible substrates as both their deformylated products have been identified in grapevine leaf lysates.^[^ ^16^ ^]^ When we performed competitive conversion experiments with a 1:1 ratio of *Vv*‐PleB‐57 **1** and *Vv*‐PxB **6**, a slightly higher product to substrate ratio was observed for **8**: **1** than **9**: **6** (Figure 4c).

### The YUCCA10 Dual Role Spans Other Clades of the Plant kingdom

The connection of ChI catabolism and auxin homeostasis on an enzymatic level sparked the question whether this reaction is observed for YUCCA10 from other plants and across other clades of the plant kingdom. When we conducted pBLAST search with *Vv*YUCCA10 to identify closely related enzymes, we found that one out of four variants of YUCCA10 from *Coffea arabica* (*Ca*YUCCA10) shares a close sequence similarity of 68% (99% query coverage). Additionally, we chose the only variant of YUCCA10 reported in NCBI from *A. thaliana* (*At*YUCCA10) with a sequence similarity of only 49% (99% query coverage) and expressed all enzymes heterologously in *E. coli* BL21 (Figures S26 and S27). *Ca*YUCCA10 readily deformylated both *Vv*‐PleB‐57 **1** and *Po*‐PleB **1*n*** to *Vv*‐DPleB‐51 **8** and *Po*‐DPleB **8*n***, respectively, when overexpressed in *E. coli* lysate and as purified enzyme (Figure S28). Additionally, it converted **15** into **16** (Figure S29A). In contrast, *At*YUCCA10 was only able to convert **15** into **16**, but did not accept *Vv*‐PleB‐57 **1** or **1*n*** as substrate (Figure S29B). Notably, *At*YUCCA10 produced **16** significantly faster than the other tested enzymes, as indicated by an about 1.5‐fold higher **16** to **17** ratio and almost complete substrate conversion (Figure S30). The deformylation reaction catalyzed by *Ca*YUCCA10 is noteworthy, as *Coffea* belongs to the asterid clade within pentapentaloes, whereas *Vitis* belong to rosids. Therefore, we investigated if *C. arabica* leaves also contain deformylated phyllobilins like *Vitis*. Indeed, the lysate of coffee leaves contained the deformylated phyllobilins with *m/z* = 633.2925 [M + H]^+^ and *m/z* = 631.2768 [M + H]^+^ (Figures S31 and S32). Again, no IPA **15** or IAA **16** was detected. Alpha fold models of *Vv*YUCCA10 and *Ca*YUCCA10 revealed similar active site architectures and a conserved tyrosine (Tyr) as putative catalytic residue, which requires further investigation (Figures S33 and S34). Interestingly, *At*YUCCA10 lacked this residue and featured an active site distinct from the YUCCAs of the perennial plants (Figure S35). Overall, these results confirm likely widespread dual roles of YUCCA10s in Chl‐catabolism and auxin biosynthesis in perennial higher land plants.^[^ ^59^ ^]^ The proposed dual roles for *Vv*YUCCA10 and *Ca*YUCCA10 require further investigation, for example, by in vivo experiments when genetic manipulations become more accessible in these plants.^[^ ^60^ ^]^ It is noteworthy that the recent discovery of hypermodified PluBs from banana peel containing abscisic acid‐like substituents may suggest an additional link between Chl catabolism and plant hormones.^[^ ^61^ ^]^

## Conclusion

In this work, we aimed to identify novel enzymes involved in Chl degradation by integrating transcriptomic data with a Chl catabolite pull‐down assay of leaf extracts from grapevine, combined with the plants’ well‐characterized proteome. The comparison of transcriptomic co‐expression networks and the pulled‐down proteins led to the unexpected identification of YUCCA10, an FMO normally implicated in the biosynthesis of auxin **16**, a central pant hormone that controls plant growth and, for example, delays senescence in leaves. Although plants contain a large number of FMOs compared to the other kingdoms of life, mostly YUCCA enzymes have previously been functionally investigated.^[^ ^62^ ^]^ In vitro assays now confirmed that YUCCA10 homologs from different plants across *Pentapetalae* are able to deformylate several high molecular weight ChI catabolites (producing, e.g., DPleBs) with species‐specific preferences. As DPleBs are the major ChI catabolites in extracts of *Vitis vinifera* leaf, the discovered transformation explains the branching of the PaO/phyllobilin pathway in this plant. Thus, previously postulated Chl catabolic steps could now be confirmed and shown to involve a BVMO‐type reaction catalyzed by YUCCA10 affording a formate ester intermediate, which undergoes rapid hydrolysis and tautomerization to a distinct γ‐lactam A ring. Up until now, an enzymatic reaction on a Chl catabolite on the level of PluBs had only been described for CYP89A9 in *A. thaliana*, the primary model plant for studying enzymatic transformations in chlorophyll degradation.^[^ ^9^ ^]^ However, CYP89A9 acted specifically on PluBs, whereas PleBs such as **1** investigated in this work, were not converted. It is remarkable that YUCCA10 and CYP89A9 rely on different cofactors—FAD and heme—to catalyze these distinct and mechanistically challenging deformylation reactions. Notably, the observed inhibition of YUCCA10 by Chl catabolites (DPleB **8**) already at sub‐physiological concentrations during the in vitro conversion of precursor **15** into auxin **16** provides a mechanistic basis of how Chl degradation may drive a positive feedback loop that reduces auxin levels, further emphasizing the role of YUCCAs in leaf senescence.^[^ ^20^ ^]^

Taken together, the observed feedback inhibition of *Vv*YUCCA10 by Chl catabolites is consistent with the long‐proposed auxin gradient theory, potentially explaining how the onset of senescence in *Vitis* leaves and berries causes the local reduction of auxin levels. Our findings furthermore reveal the first evidence of a YUCCA enzyme involved in a plant metabolic pathway beyond auxin biosynthesis, underscoring the notion that Chl catabolites play more significant roles in plants than mere degradation byproducts. These hormone–Chl catabolite connections may reflect a long‐established evolutionary process in plants, and enable in particular perennial plants to efficiently regulate the relocation of nutrients from senescing leaves to storage organs (e.g., roots, stems) for use in future growth cycles.

## Supporting Information

The authors have cited additional references within the Supporting Information.^[^ ^63^, ^64^, ^65^, ^66^, ^67^, ^68^, ^69^, ^70^ ^]^

## Conflict of Interests

The authors declare no conflicts of interest.

## Supporting information



Supporting Information

## Data Availability

The data that support the findings of this study are available from the corresponding author upon reasonable request.
