# Supplementary material for: Unexpected Dual Function of Plant YUCCA Enzymes Links Chlorophyll Catabolism to Auxin Homeostasis
Source: Angew Chem Int Ed Engl. 2026 Jan 21;65(9):e25568. doi: 10.1002/anie.202525568 (PMC12930011; doi:10.1002/anie.202525568)
Supplement: Supplementary file 1 — Supporting Information [file ANIE-65-e25568-s001.docx]

Supporting Information

Unexpected Dual Function of Plant YUCCA Enzymes Links Chlorophyll Catabolism to Auxin Homeostasis

Sina Rütschlin^[a]^, Lei Zhang^[a]^, Cornelia A. Karg^[b]^, Michael Zwerger^[c]^, Johanna Gostner^[c, d]^,Simone Moser^[b]^, and Robin Teufel*^[a]^

[a] Dr. S. Rütschlin, Dr. L. Zhang, Prof. Dr. R. Teufel
Department of Pharmaceutical Sciences, Pharmaceutical Biology
University of Basel
Klingelbergstrasse 50, 4056 Basel (Switzerland)
E-mail: robin.teufel@unibas.ch

[b] Dr. C. A. Karg, Prof. Dr. S. Moser
Department of Pharmacognosy, Institute of Pharmacy
University of Innsbruck
Innrain 80/82, A-6020 Innsbruck (Austria)

[c] Dr. M. Zwerger, Assoc.-Prof. Dr. Mag. J. Gostner
Institute of Medical Biochemistry
Medical University of Innsbruck
Innrain 80/82, A-6020 Innsbruck (Austria)

[d] Core Facility Metabolomics II
Institute of Medical Biochemistry
Medical University of Innsbruck
Innrain 80/82, A-6020 Innsbruck (Austria)

**Supporting Information Contents**

[**Materials and Methods** 3](#_Toc219707591)

[**Table S1** **Chlorophyll degradation genes and corresponding enzymes of *Vitis vinifera*.** 14](#_Toc219707592)

[**Table S2 Enzymes used for calculation of phylogeny with MEGA11.^67^** 15](#_Toc219707593)

[**Table S3 Localization prediction by TMHMM and Target P.2.0.** 16](#_Toc219707594)

[**Table S4 Overview on used chlorophyll catabolites in this manuscript and their published NMR data.** 17](#_Toc219707595)

[**Figure S1 Plant Material used for this study.** 18](#_Toc219707596)

[**Figure S2** **Co-expression network generated by the ATTED network drawer.** 19](#_Toc219707597)

[**Figure S3 Co-expression network generated by the ATTED network drawer with the *vvyucca10* gene as bait.** 20](#_Toc219707598)

[**Figure S4 SDS-Page of purified *Vv*YUCCA10-MBP and cofactor determination.** 21](#_Toc219707599)

[**Figure S5 Unrooted phylogenetic tree based on the maximum likelihood model with LG and G.** 22](#_Toc219707600)

[**Figure S6 Identification of *Po*-PleB 1*n* by HR-MS^2^ and UV.** 23](#_Toc219707601)

[**Figure S7** **Identification of *Po*-DPleB 8*n* by HR-MS^2^ and UV.** 24](#_Toc219707602)

[**Figure S8 Comparison of *Po*-DPleB 8*n* and *Vv*-DPleB-51 8 by HR-MS in positive ion mode to an isolated standard of *Vv*-DPleB-51 8.** 25](#_Toc219707603)

[**Figure S9** **Incorporation of ^18^O-labelled oxygen into *Po*-PleB 8*n*.** 26](#_Toc219707604)

[**Figure S10** **Fragments of ^16^O-*Po*-DPleB and ^18^O-*Po*-DPleB.** 27](#_Toc219707605)

[**Figure S11** **IPA 15 conversion to IAA 16 by *Vv*YUCCA10-MBP.** 28](#_Toc219707606)

[**Figure S12** **Enzymatic conversion of IPA 15 to IAA 16 catalyzed by *Vv*YUCCA10 and area under the curves to confirm enzymatic activity.** 29](#_Toc219707607)

[**Figure S13** **HPLC-DAD chromatogram shown at 254 nm of lysate of yellow-green grapevine leaves and extracted ion chromatograms (XIC) shown for important chlorophyll (Chl) catabolites and auxin metabolites IPA 15 and IAA 16.** 30](#_Toc219707608)

[**Figure S14** **XIC traces for *Vv*-DPleB-51 8 and *Vv*-DPxB-63 9 detected in lysate of yellow-green grapevine leaves by HR-MS^2^.** 31](#_Toc219707609)

[**Figure S15 IAA 16 spiked into grapevine leaf lysate to determine detection limit with HR-MS Orbitrap.** 32](#_Toc219707610)

[**Figure S16 Relative phyllobilin and tryptophan metabolite profiles in green, yellow, and brown *A. thaliana* siliques.** 33](#_Toc219707611)

[**Figure S17** **Inhibition test of *Po*-PleB 1*n* conversion with a 10-fold excess of IAA 16 towards 1*n*.** 34](#_Toc219707612)

[**Figure S18** **HPLC-DAD chromatogram shown at 254 nm of control reactions for conversion reaction of *Cj*-PleB-2 19 to its deformylated version 20 with denatured enzyme *Vv*YUCCA10-MBP.** 35](#_Toc219707613)

[**Figure S19 *Cj*-PleB-2 19 conversion assays with NADPH and *Vv*YUCCA10-MBP.** 36](#_Toc219707614)

[**Figure S20** **HPLC-DAD chromatogram shown at 254 nm of control reactions for conversion reaction of *Cj*-PrB 7 to deformylated DPrB 14 with denatured enzyme *Vv*YUCCA10-MBP shown at 254 nm.** 37](#_Toc219707615)

[**Figure S21** ***Cj*-PrB 7 conversion assays with NADPH and *Vv*YUCCA10-MBP.** 38](#_Toc219707616)

[**Figure S22** **HPLC-DAD chromatogram shown at 254 nm of control reactions for conversion reaction of *Cj*-PxB 6 to deformylated *Vv*-DPxB-63 9 conversion assay with denatured enzyme *Vv*YUCCA10-MBP shown at 254nm.** 39](#_Toc219707617)

[**Figure S23** ***Cj*-PxB-2 6 conversion assays with NADPH and *Vv*YUCCA10-MBP.** 40](#_Toc219707618)

[**Figure S24 Results of conversion assays with cyclohexanone and phenyl pyruvate as substrate for *Vv*YUCCA10.** 41](#_Toc219707619)

[**Figure S25 Chromatogram and SDS-gel analysis of size exclusion chromatography (SEC) of MBP-tagged *Vv*YUCCA10.** 42](#_Toc219707620)

[**Figure S26 SDS-PAGE analysis of different fractions collected during affinity purification (IMAC) of MBP-tagged *At*YUCCA10.** 43](#_Toc219707621)

[**Figure S27 SDS-PAGE analysis of different fractions collected during affinity purification (IMAC) of MBP-tagged *Ca*YUCCA10.** 44](#_Toc219707622)

[**Figure S28** **Control reactions for *Vv*-PleB-57 1 and *Po*-PleB 1*n* conversion to *Vv*-DPleB-51 8 and *Po*-DPleB 8*n* catalyzed by *Ca*YUCCA10-MBP.** 45](#_Toc219707623)

[**Figure S29** **IPA 15 conversion to IAA 16 catalyzed by *Ca*YUCCA10-MBP and *At*YUCCA10-MBP.** 46](#_Toc219707624)

[**Figure S30 Bar chart comparing IPA 15 decay to enzymatic reaction catalyzed by *At*YUCCA10-MBP and *Ca*YUCCA10-MBP.** 47](#_Toc219707625)

[**Figure S31** **HPLC-DAD chromatogram shown at 254 nm of lysate of coffee leaves and extracted ion chromatograms (XIC) shown for chlorophyll (Chl) catabolites detected.** 48](#_Toc219707626)

[**Figure S32** **Relative phyllobilin profiles in green, yellow, and brown *Coffea arabica* leaves.** 49](#_Toc219707627)

[**Figure S33** **Alphafold model prediction of *Vv*YUCCA10 and *Ca*YUCCA10 with co-factor FAD (blue).** 50](#_Toc219707628)

[**Figure S34** **Alphafold model prediction of *Vv*YUCCA10 with putative Tyr as catalytic residue.** 51](#_Toc219707629)

[**Figure S35** **Alphafold model prediction of *Vv*YUCCA10 and *At*YUCCA10 with co-factor FAD (blue).** 52](#_Toc219707630)

[**SI References** 53](#_Toc219707631)

# **Materials and Methods**

**Chemicals and solvents**

Chemicals and reagents were purchased from Sigma Aldrich if not indicated otherwise. Solvents for HPLC and UHPLC analysis were used at LC-MS grade. Enzymes and additional material used for molecular cloning were purchased either from New England Biolabs (NEB), Thermo Fisher Scientific or Sigma-Aldrich. Custom synthesized DNA was obtained from IDT technologies (Integrated DNA Technologies, Inc., USA). DNA sequencing was carried out by Microsynth (Microsynth AG, Switzerland). For protein purification, equipment (Ni^2+^-IMAC column, MBP-Trap columns and gel filtration columns) from Cytiva (Cytiva™, USA) was used. Concentration of proteins was carried out in centrifugal devices from Thermo Fisher Scientific and PALL (Pall Corporation, USA). SDS-PAGE and Agarose gel electrophoresis were carried out in equipment from Cleaver Scientific (UK).

**LC-MS and HPLC analysis**

For HPLC-DAD analysis, a Shimadzu chromatographic system 20A, equipped with a DAD module (SPD-M20A DAD, Shimadzu) and an analytical Sunfire® C18 column (150 x 3 mm ID, 3.5 µm, Waters, USA) without guard column was used. Pre-equilibration of the column was conducted with A = 85% H_2_O + 0.1 % formic acid (FA) and B = 15% acetonitrile (ACN) + 0.1 % FA at a flow rate of 0.4 mL/min. Eluents A = H_2_O + 0.1 % FA and B = ACN + 0.1 % FA were used with the same flow rate for the following gradient: T_0 min_ = 15 % B, T_5 min_ = 15 % B, T_20 min_ = 65 % B, T_21 min_ = 100 % B, T_31 min_ = 100 % B, T_32 min_ = 15 % B (oven temperature 25°C, total running time 37 min). Absorption was monitored at from 100 to 800 nm.

For LC-MS analysis a Shimadzu LCMS-8030 Triple Quad Mass Spectrometer was used, equipped with DAD module (SPD-M20A DAD, Shimadzu, Japan) and a CAD module (Dionex Corona Veo RS, ThermoScientific, USA). An analytical SunFire® C18 column (150 x 3 mm ID, 3.5 µm, Waters, USA) without guard column was used. Pre-equilibration of the column was conducted with A = 85% H_2_O + 0.1 % formic acid (FA) and B = 15% acetonitrile (ACN) + 0.1 % FA at a flow rate of 0.4 mL/min. Absorption was monitored from 190 – 800 nm. Samples were analysed in MS ESI positive and negative mode with a capillary voltage of 3 kV, 250°C DL temperature, 400 °C heat block temperature and 3 L/min nebulizing gas flow. Eluents A = H_2_O + 0.1 % FA and B = ACN + 0.1 % FA were used with the same flow rate as described above for the following gradient: T_0 min_ = 15 % B, T_5 min_ = 15 % B, T_20 min_ = 65 % B, T_21 min_ = 100 % B, T_31 min_ = 100 % B, T_32 min_ = 15 % B (oven temperature 25°C, total running time 37 min). MS data (range 100-1000 m/z) was recorded from 0.002 to 37 min.

HR-UHPLC-Orbitrap measurements of grapevine lysates were performed with an Agilent 1290 Infinity chromatographic system (Agilent Technologies, USA) equipped with an Aquity UHPLC® BEH C18 column (2.1 x 150 mm ID, 1.7 µm, Waters, USA) with a guard column. Pre-equilibration of the column was conducted using A = 95% H_2_O + 0.1 % FA and B = 5% ACN + 0.1 % FA at a flow rate of 0.35 mL/min. The same flow rate was used for the measurements with the following gradient: T_0 min_ = 5 % B, T_0.5 min_ = 5 % B, T_15.5 min_ = 100 % B T_17.3 min_ = 100 % B, T_18 min_ = 5 % B (oven temperature 40°C, total running time 21 min). The flow was flushed into the waste for 3 min at the beginning of the method. MS data (range 100-1500 m/z) was recorded from 3 to 21 min.

HR-UHPLC-Orbitrap measurements of *A. thaliana* siliques and coffee leaves were performed on a Vanquish system (Thermo Scientific, Waltham, Massachusetts, USA) consisting of a quaternary pump, an auto-sampler, a column oven, and a diode-array detector connected to a Thermo Scientific Exploris 120 Orbitrap HRMS unit. Separation was carried out on a Waters ACQUITY UHPLC BEH C18 column (2.1 mm x 50 mm, particle size = 1.7 µm, Waters, USA) protected by a Phenomenex SecurityGuard ULTRA guard cartridge system (i.e., a UHPLC C18 pre-column) (Phenomenex, Aschaffenburg, Germany). The mobile phase comprised ammonium formate (c = 10 mM) in water with 0.1% formic acid (A) and acetonitrile (B). The flow rate, column oven temperature, auto-sampler temperature, and injection volume were adjusted to 0.3 mL/min, 35 °C, 20 °C, and 0.5 µL for *A. thaliana* and 5 µL for coffee leaves, respectively, and the following gradient was used: T_0 min_ = 10 % B, T_3 min_ = 20 % B, T_10 min_ = 20 % B, T_11 min_ = 25 % B, T _17 min_ = 30 % B, T _20 min_ = 95 % B, T _26 min_ = 95 % B. Finally, the column was re-equilibrated with the original solvent composition (i.e., 10 % B) for 12 minutes, which corresponds to a total run time of 38 minutes. MS data (range 100-1500 m/z) was recorded from 1 to 26 min with a resolution of 60 000 FWHM for MS1.

For HPLC-DAD-MS analysis of the pull-down probe, an Agilent 1260 Infinity II HPLC system coupled with a G7115A 1260 DAD WR detector as well as an Agilent MS Single Quad detector with ESI (Agilent Technologies, USA) was used. Separation was performed on a LiChrospher 100 RP-18 column (125 x 4 mm ID, 5 µm, Merck, Germany) with guard column. The mobile phase comprised ammonium formate (c = 10 mM) in water with 0.1% formic acid (A) and acetonitrile (B). The flow rate, column oven temperature, and injection volume were adjusted to 0.5 mL/min, 25 °C, and 10 µL, respectively, and the following gradient was used: T_0 min_ = 5 % B, T_5 min_ = 5 % B, T_20 min_ = 95 % B, T_25 min_ = 95 % B, T _27 min_ = 5 % B. Absorption was monitored at from 100 to 800 nm.

UHPLC-MS analysis for measuring Trp metabolites in *A. thaliana* was performed on an Agilent 1290 UHPLC system (Agilent, Santa Clara, CA, USA) comprising binary pump, thermostated autosampler, and column oven. Separation was performed on a Poroshell 120 EC-C18 column from Agilent (50 mm x 2.1 mm, 2.7 µm particle size) with a C18 guard column. The mobile phase consisted of water (A) and acetonitrile (B), both containing 0.35% formic acid. An elution gradient was chosen going from 0% B to 35% B in 3 min, rising to 98% B in 1 min, holding this concentration for 0.5 min and returning to the initial conditions for 2 min for column re-equilibration. The injection volume, column temperature and flow rate were 3 µL, 30°C, and 0.6 mL/min, respectively. During analysis the samples were kept at 20°C. Detection was carried out on an AB Sciex QTRAP 6500+ mass spectrometer (AB Sciex Pte. Ltd., Framingham, MA, USA) in ESI positive polarity. Target analytes (Trp and metabolites: anthranilic acid, quinolinic acid, kynurenic acid (KynA), kynurenine (Kyn), tryptophan (Trp), neopterin (Neo), IPA, IAA) were quantitated via multiple reaction monitoring (MRM). The source parameters were set as follows: Ion spray voltage (IS) 5200 V, ion source temperature (TEM) 650°C, curtain gas (CUR) 38 psig, collision gas (CAD) 9 psig, Ion source gases (IS) 1 and 2 were at 80 and 65 psig, respectively.

Semi-preparative HPLC was used to purify *Vv*-DPleB-51 on an Agilent chromatographic HP1100 system, equipped with a DAD module (DAD 996, Waters), degassing unit and a semi-preparative Xbridge® BEH C18 column (150 x 10 mm ID, 5 µm, Waters) with a guard column. Pre-equilibration of the column was conducted with A = 88% 10 mM NH_4_Ac (adjusted with ammonia and FA), pH = 7 and B = 12% acetonitrile (ACN) at a flow rate of 2.5 mL/min. Eluents A = 10 mM NH_4_Ac, pH = 7 and B = ACN were used with the same flow rate for the following gradient: T_0 min_ = 12% B, T_2 min_ = 12% B, T_12 min_ = 20% B, T_30 min_ = 80% B, T_40 min_ = 100 % B (oven temperature 30°C, total run time: 45 min). Absorption was monitored from 180 to 400 nm.

Gradient changes for individual measurements are indicated in the respective section. Otherwise, the described gradients were used for the instruments.

**GC-MS analysis**

GC-MS analysis was used to study possible conversion products of cyclohexanone with *Vv*YUCCA10-MBP. Samples were measured on a Hewlett-Packard GC/MS system (Agilent G1503A- 6890 Plus GC) with a mass selective detector (5973, MSD) and a 59864B ionization gauge controller (Agilent Technologies) equipped with a J&W DB-225ms GC column (30 m; 0.25 mm i.d.; film thickness 0.25 μm; Agilent Technologies). The following method was used with a helium flow of 0.7 mL/min and an injector temperature of 240°C: Oven: 60°C hold for 1 min, increase to 240°C at 10 °C/min followed by 5 min at 240°C (total run time: 24 min). The solvent-cut time was 3 min, transfer line temperature: 230°C, Ion source: 230°C, Quadrupole: 150°C, EI: 70 eV, the scan range was 50-700 amu. Samples were injected in hexane with an injection volume of 1 µL and a split mode of 1:10.

**Nomenclature of Chl catabolites**

Phyllobilin structures identified from different plant species are usually named with the initials of the botanical name of the plant source as a prefix, the type of the phyllobilin core structure, and a number indicating the polarity in reversed-phase chromatography or a consecutive number, e.g., Ep-PleB-1. In line with the recently revised nomenclature,^5^ previously described Chl catabolites, such as NCCs and YCCs, are now referred to as PleBs and PxBs, respectively.

**Collection of plant materials**

Yellow-green grapevine leaves were collected from a private garden in Basel, Switzerland in October 2024 (47.56284°N, 7.57842°E). Siliques of *A. thaliana* were provided by Erwann Arc and Ilse Kranner, Institute of Botany, University of Innsbruck. *Coffea arabica* leaves were obtained from a commercially purchased plant*.* Plane tree leaves (*Platanus* *occidentalis*) were collected in Basel, Switzerland in November 2024. If not used for extraction, all leaves were frozen in liquid nitrogen and stored at -80°C. Pictures of the used plant material are shown in Figure S1.

**Co-expression data analysis with ATTED-II**

For determination of co-expressed genes with the known Chl catabolite enzymes from Chl catabolism steps, the ATTED-II^40^ network drawer was used and fed with the following genes from *Vitis vinifera:* pheophytinase NCBI# 100265442, pheophorbide a oxygenase #100256831, red chlorophyll catabolite reductase #100252439, ethylene-responsive transcription factor ERF017 #100247370^63^, protein STAY-GREEN homolog #100265112, magnesium dechelatase SGRL #100252353, TIC 55 #100259354, chlorophyll(ide) b reductase NOL #100265035. For drawing of the co-expression network, platform was set to automatic, Dis. type to Graphviz, Coex. option to add many genes and PPI option to add a few genes. Figure S2 and S3 show the generated co-expression network and the networks centered around *Vv*YUCCA10 (Uniprot: F6HQ23), respectively. Further details for the sequences used are listed in the Supporting Information Table S1.

**Extraction of *Po*-PleB 1*n* from plane tree leaves**

For extraction of *Po*-PleB **1*n***, the leaves were ground in liquid nitrogen and stirred in 3 L acetone. The leaf debris was separated by filtration with a Büchner funnel and the washed with acetone two times. The acetone was removed on a rotary evaporator in the dark until only some liquid was left. Dichloromethane (DCM) was used to extract the residual liquid until it was almost colorless. Then, the DCM was removed with a rotary evaporator and the residue stored at -20°C until further use. The process of debris extraction was repeated until all *Po*-PleB **1*n*** was successfully removed. For control of removal, LC-MS analysis was performed as indicated in the LC-MS and HPLC section. Flash chromatography (Puriflash X, Advion Interchim scientific®, USA) was performed for a first separation of the acetone extract using an Interchim PF-15SIHP-F0040 column. 5% MeOH was used as starting condition and then increased to 20% MeOH. For the dry sample load, 2 g acetone extract was dissolved in MeOH and supplemented with 1.5x volumes of Celite® 545 (3 g, Merck, Germany). A rotary evaporator was used to remove all solvents, the residual material was grinded to a fine powder and the powder was loaded into a BGB EZPak empty cartridge (BGB analytik, Switzerland), covered with sand and installed onto the flash chromatography system. *Po*-PleB **1*n*** was eluted with a flow rate of 22 ml/min at T_5 min_ = 5% MeOH up to T_100min_ = 20% MeOH. Each fraction contained 22 ml and was checked by TLC for *Po*-PleB **1*n***. *Po*-PleB **1*n*** was further purified by semi-prep HPLC on a Sunfire C18 column (Waters™, USA) with A = 10 mM NH_4_OAc, pH 7 and B = ACN. The following gradient was used: T_0-2 min_: 25% A, T_2-12 min_: 25-41% A, T_12-12.1 min_: 41-100% A, T_12.1-17 min_: 100% A. The flow rate was 3 mL/min. *Po*-PleB **1*n*** eluted roughly at 7.9 min and the fraction was lyophilized to result in around 2 mg of pure *Po*-PleB **1*n***. *Po*-PleB **1*n*** was dissolved in 2 ml MeOH and then evenly divided into 20 dark Eppendorf tubes to create 20 aliquots which were dried under nitrogen flow and flash frozen in liquid nitrogen. The aliquots were stored at -20°C until further use. Purity and identity of *Po*-PleB **1*n*** was checked by LC-MS analysis with a Triple Quad and Orbitrap mass spectrometer described in the LC-MS and HPLC section. The *Po*-PleB Chl catabolite has been fully characterized by NMR and has been assigned to either the ‘*epi*’ or ‘*n*’ series of catabolites by comparison of retention times and standards.^11^

**Lysate preparation of vine leaves for pull-down assay**

To prepare a grapevine leaf lysate, 1 g of fresh or frozen leaves (Figure S1A) were ground in liquid nitrogen in a mortar with a small amount of sea sand. The resulting fine powder was transferred to a chilled mortar on ice. Then, 10 – 15 mL of 4 mM NH_4_OAc buffer (pH 7.0) containing 100 mM NaCl, 10% glycerol and 1mM PMSF were added and the mixture was ground thoroughly. The slurry was transferred to a cold beaker on ice and subjected to ultrasonication (1s pulse, 3s pause; 3min pulse time, 2 repetitions). Afterwards, the slurry was centrifuged at 18000xg at 4°C for 30 minutes. The supernatant was collected for affinity chromatography and metabolomic studies described later. Note: if particles remained in the supernatant after centrifugation, it was filtered before use.

**Synthesis of *Vv*-DPleB-51-biotin probe**

*Vv-*DPleB-51 (4.09 mg, 6.47 µmole) was dissolved in 250 µl of DMSO in an Eppendorf tube. 2.5 equivalents each of HOBt (2.04 mg) and EDCl (3.08 mg) were dissolved in 100 µl of DMSO and added to the starting material. After incubation at room temperature on a shaker (800 rpm) for 1h, 3 equivalents of Et_3_N (1.5 µl) were added. Biotin-polyethylene glycol (PEG)_7_-amine (4.08 mg, 1.1 equivalents) was dissolved in 100 µl of DMSO and added slowly to the mixture in the Eppendorf tube and incubation was continued at room temperature (shaker set to 700 rpm) for 24 h. The reaction progress was monitored by analytical HPLC-MS. The reaction mixture was purified by flash chromatography (Waters SepPak column 5 g) with increasing concentrations of MeOH in water (10, 20, 30, 40 and 60% MeOH). The product eluted with 60% MeOH, and the fraction was lyophilized to yield 3.2 mg of *Vv*-DPleB-51-biotin probe (2.65 µmol, 41,0 %). The probe was characterized by HR-ESI-MS: m/z_calculated_ (C_60_H_89_O_16_N_8_S) = 1209.6112 [M+H]^+^; m/z_found_ = 1209.6099 [M+H]^+^ (Δ = - 1.07 ppm).

**Mass spectrometry-based affinity chromatography employing the *Vv*-DPleB-51-biotin probe and grapevine leaf lysate**

25 μL of streptavidin agarose bead suspension was transferred to an Eppendorf tubes and washed with washing buffer (10 mM HEPES-NaOH, 50 mM KCl, 1 mM EDTA-disodium salt, 10% glycerol, pH 7.9) by centrifugation at 1000-3000 rpm for 1 min in a table top centrifuge. The washed beads were resuspended in 100 μl of washing buffer. 5 μL of 2 mM *Vv*-DPleB-51-biotin conjugate was added to the beads. The *Vv*-DPleB-51-biotin conjugate stock was prepared by dissolving synthesized *Vv*-DPleB-51-biotin - conjugate in 10 µL of DMSO to a concentration of 40 mM and subsequent dilution with pull-down buffer. The mixture was incubated at rt for 20 min. After washing three times with 1 mL of washing buffer, grapevine leaf lysate (total 100 μg of protein as determined by Bradford assay) was added and the mixture incubated at 4°C for 30 min. The beads were washed two times with 1 mL of washing buffer. For each washing step, the Eppendorf tube was changed to make sure no unspecific protein residues remained. The mixture was centrifuged at 4000 rpm for 3 min at 4°C and the supernatant discarded. 50 μL of elution buffer (50 mM Tris-HCl, 5% SDS, pH 7.5) was added and the mixture denatured at 95°C for 5 min. After centrifugation at 3000 rpm for 3 min at 4°C, the supernatant was brought to the Proteomics core facility (Biozentrum Basel, Switzerland) for further processing and analysis. In short, in the protein core facility, Tris(2-carboxyethyl)phosphine (TCEP) and triethylammonium bicarbonate (TEAB) were added to a final concentration of 10 mM and 100 mM, respectively to 50 µL of eluate sample. Then, the samples were sonicated with a PIXUL® Multi-Sample Sonicator (30 sec on, 30 sec off, 10 cycles; 10 min). To 5 µL sample, 195 µL urea solution (1 M) was added to perform fluorescence protein concentration measurement with an Infinite® M-Plex multimode microplate reader (Tecan Group AG, Switzerland). Iodoacetamide solution was added to the rest of the solution to a final concentration of 20 mM and kept in the dark at 25°C at 500 rpm for 30 minutes. The incubated protein solution was filled to 90 uL and an SP3 procedure performed. In total, 300 ng trypsin were added to the sample and it was incubated at 37°C overnight. After elution and SpeedVac concentration of the peptides, 50 ng of peptide were injected into a timsTOF Ultra platform in DDA mode. After data processing, the Proteomics core facility provided a list with 873 detected proteins annotated against the Uniprot proteome of *Vitis vinifera* (https://www.uniprot.org/proteomes/UP000009183).

**Gene cloning, heterologous protein expression, and purification procedures**

The *vvyucca10* gene sequence without introns was retrieved from NCBI accession # 100245859 and its associated mRNA transcript ID XM_002269808.5. The sequence was optimized for expression in *E. coli* with the IDT technologies Gene Optimization Tool and subsequently ordered as gBlocks™ gene fragment for SLIC Cloning into pNIC vectors from IDT technologies (Integrated DNA Technologies, Inc., USA). To insert the gene fragment into pNIC vectors carrying a kanamycin resistance, the gene was flanked with the respective overhangs for T4 DNA Polymerase (GGT ACC GAG AAC CTG TAC TTC CAA TCC in front of the removed start codon and TAA TAA GAC GGT CTC CAG TAA AGG TGG ATA CGG ATC after the removed stop codon). Linearized pNIC-MBP for maltose binding protein (MBP)-tagged enzyme was prepared by PCR. For SLIC ligation, the published protocol was used^64^ with the following modifications: 4-fold molar excess of the DNA insert was used over the vector. For transformation, competent E. *coli* XL1blue cells were thawn on ice, the SLIC mixture was added, incubated for 30 min on ice, treated by heat shock for 30-45 sec at 42 °C, placed back on ice and incubated for 5 min. The cells were plated on LB agar plates containing 50 µg/mL kanamycin and 5% (w/v) sucrose and incubated overnight at 37°C. The next day, a clone was picked, inoculated into 5 mL LB broth containing 50 µg/mL kanamycin and grown overnight in a shaking incubator at 37°C, 120 rpm. The plasmid was extracted with a FastPrep Mini Kit (Promega, USA) and confirmed by sequencing (Microsynth AG, Switzerland). 10 ng of vector were used for transformation into *E. coli* BL21 cells with the protocol described above for *E. coli* XL1blue. After a clone was picked and grown overnight in 5 mL LB broth containing 50 µg/mL kanamycin, a cryo culture with glycerol was prepared and stored at -80°C.

The *atyucca10* gene sequence without introns was retrieved from NCBI accession AT1G48910 and its associated mRNA transcript ID NM_103784.2. The *cayucca10* gene sequence without introns was retrieved from NCBI accession XP_027116742.1 and its associated mRNA transcript ID XM_027260941.2. All sequences were optimized for expression in *E. coli* with the IDT technologies Gene Optimization Tool and subsequently ordered as gBlocks™ gene fragment for SLIC Cloning into pNIC vectors from IDT technologies (Integrated DNA Technologies, Inc., USA). Localization prediction by TMHMM and Target P.2.0 can be found in the Table S3.

Heterologous protein expression of the cloned and transformed enzyme was performed according to published protocol^65^ with the following modifications: TB media supplemented with 50 µg/mL kanamycin was used and induction was started with 1 mM IPTG.

For protein purification, the cell pellet was resuspended in 2.5 mL/g buffer A (50 mM HEPES, pH 7.5, 300 mM NaCl, 10% glycerol), protease inhibitor cOmplete (Roche, Switzerland) and a spatula tip of FAD was added. Cells were lysed with a sonicator (2 s pulse, 6 s pause; 2 min total pulse time, amplitude: 40 %), centrifuged and the supernatant sterile filtered through a PVDF filter with pore size of 0.45 μm. MBPTrap purification (both Cytiva™, USA) was performed according to the protocol of the producer with an ÄKTA start system (Cytiva, USA). Fractions containing the desired protein were investigated by SDS-page, pooled together and concentrated in an Amicon® centrifugal filter (Sigma Aldrich, USA). The purified enzyme was frozen in liquid nitrogen before being stored at -80°C. Enzyme concentration was determined by its molecular weight and extinction coefficient using a Nanodrop system (Implen, Germany). Size exclusion chromatography (SEC) was carried out to estimate the amount of aggregated enzyme using a Superdex 200 GL 10/300 column (Cytiva). The column was equilibrated with 150 mM NaCl, 20 mM HEPES, 5% glycerol at pH 7.5. Proteins were run at 1 mL/min and molecular weights of the eluting main peaks were estimated from a previously generated calibration curve with proteins of known molecular weight. Figure S25 shows the chromatogram and SDS-gel analysis of VvYUCCA10-MBP. Aggregated enzyme eluted from 40 to 60 min and did not fit the pre-calculated retention time for the enzyme.

For truncation of the *vvyucca10* gene, a new gene insert was ordered sequence optimized for expression in *E. coli* and subsequently ordered as gBlocks™ gene fragment for SLIC Cloning into pNIC vectors from IDT technologies. Note: it was not possible to truncate the N-terminus of the gene insert by PCR amplification. Two experiments resulted in an amplificated product, but subsequent cloning into *E. coli* XL1blue cells and sequencing resulted for both experiments in base deletions spanning 5 to 30 bases in the middle of the gene. The two primers for truncation of the gene insert at the N-terminus were designed as such, that the forward primer was missing 11 base triplets from the N-terminus, but would still carry the SLIC overhang: *vvyucca10*_fw 5’ GGTACCGAGAACCTGTACTTCCAATCCAGCTTCAAAAAAATGCAGGAGACAGTTGTC 3’, *vvyucca10*_rev 5’ GATCCGTATCCACCTTTACTGGAGACCGTC 3’. For crystallization trials of the truncated version of *Vv*YUCCA10 (*Vv*YUCCA10_trunc-MBP) or its cleaved version, the enzyme was expressed as MBP-tagged protein as described above and purified according to protocol of the producer with the ÄKTA start system (Cytiva, USA). To cleave of the MBP-tag, TEV cleavage was performed with His-Super TEV enzyme in an 1:50 ratio at rt overnight. MBP-tag and *Vv*YUCCA10_trunc were separated with a reverse IMAC run on a with an ÄKTA start system connected to an 1mL Ni^2+^-IMAC column. Finally, size exclusion chromatography (SEC) was carried out as described above. Note: it was not possible to get pure or a batch of non-aggregated VvYUCCA10_trunc-MBP or VvYUCCA10_trunc. Five expressions and subsequent purification efforts resulted in only aggregated product based on SEC (Figure S25). However, SEC aggregates contained still active enzyme as these aliquots were tested for deformylating activity. Therefore, *Vv*YUCCA10_trunc-MBP or *Vv*YUCCA10_trunc are not suitable for crystallization.

The protein sequences were used to calculate Alphafold models using the Alphafold Server.^66^ Calculated models were visualized with PyMOL software.

**Co-factor determination of *Vv*YUCCA10-MBP**

LC-MS analysis was used for determination of the co-factor bound to *Vv*YUCCA10-MBP*.* 0.0025 µmol of enzyme were denatured at 98°C for 10 min and centrifuged for 5 min at full speed with a table top centrifuge at 4°C. For determination of the mass of the co-factor, a Triple Quad LC-MS was used and 30 µL of the supernatant injected. The gradient and settings of the instrument are described in the LC-MS and HPLC section. Figure S4B shows the LC-MS data for confirmation of the FAD co-factor.

**Phylogenetic tree**

Evolutionary analyses were conducted with MEGA11.^67^ The sequences were first curated from literature and subsequently prepared for MEGA11 by conversion to a MAFFT file (https://mafft.cbrc.jp/alignment/server/index.html, retrieved 01/2025). The analysis included 17 amino acid sequences (Table S2). Based on the generated alignment file, MEGA11 suggested the JTT matrix-based model using the model tool to find the right tree model. The final tree was constructed based on maximum likelihood with the model LG and rates among sites G mode. The phylogenetic tree is drawn to scale, with branch lengths measured in the number of substitutions per site (Figure S5).

**Enzyme assays**

**Redox partner of *Vv*YUCCA10-MBP**

For conversion reactions to determine the redox partner, fresh 10 mM stocks of NADH and NADPH in MilliQ were prepared and directly used for the assay. *Po*-PleB **1*n*** was prepared as 3.1 mM stock in 1:1 MilliQ:MeOH in a dark Eppendorf tube. 5 µM *Vv*YUCCA10-MBP was converted with 38.8 µM Po-PleB **1*n*** substrate either without, with 1 mM NADH or 1 mM NADPH in a total volume of 100 µL in 20 mM Tris-HCl buffer, pH 8 for 20 min at 28°C, 600 rpm in the dark. The reaction was quenched by adding 200 µL of MeOH and centrifugation at 4°C for 15 min at full speed with a table top centrifuge. 30 µL of supernatant was subjected to HPLC-DAD analysis. The experiment was carried out in one replicate.

***Vv*-PleB-57 1/*Po*-PleB 1*n*** **conversion to *Vv*-DPleB-51 8/*Po*-DPleB 8*n*** **with *Vv*YUCCA10-MBP and *Ca*YUCCA10-MBP**

For *Vv*-PleB-57 **1**/*Po*-PleB **1*n*** conversion reaction, a fresh 10 mM stock of NADPH was prepared in 25 mM HEPES, pH 7.5, 150 mM NaCl and 5% glycerol (buffer A). *Vv*-PleB-57 **1** or *Po*-PleB **1*n*** was prepared as 3 mM or 3.1 mM stock in 1:1 MilliQ:MeOH in a dark Eppendorf tube. 5 µM *Vv*YUCCA10-MBP were incubated with 1 mM NADPH and 100 µM phyllobilin substrate for 20 min at 30°C and 600 rpm in the dark in buffer A. For the reaction with *Ca*YUCCA10-MBP, FAD was added to a final concentration of 30 µM. The reaction was quenched with 200 µL MeOH, centrifuged for 5 min at full speed in a table top centrifuge and 30 µL of supernatant subjected to HPLC-DAD analysis. 2 µL of the sample were further analyzed by UHPLC-Orbitrap as described in the LC-MS and HPLC section. Control reactions were performed without *Vv*YUCCA10-MBP, *Vv*YUCCA10-MBP denatured at 98°C for 10 min or without redox partner NADPH to ensure conversion by enzymatic activity. Figure S6-S8 show HPLC-DAD and LC-MS data for confirmation of *Vv*-DPleB-51 **8** and *Po*-DPleB **8*n*** production. Figure S28 shows HPLC-DAD and LC-MS data for confirmation of *Vv*-DPleB-51 **8** and *Po*-DPleB **8*n*** production and control reactions for *Ca*YUCCA10-MBP. The experiments were carried out in triplicates.

**Indole-3-pyruvic acid (IPA) 15 conversion to indole-3-acetic acid (IAA) 16 with *Vv*YUCCA10-MBP, *At*YUCCA10-MBP and *Ca*YUCCA10-MBP**

For IPA **15** conversion reaction, a fresh 10 mM stock of NADPH was prepared in buffer A. IPA **15** was prepared as a fresh 10 mM stock in MeOH and subsequently diluted to 1 mM by 1:10 dilution in buffer A. 5 µM purified *Vv*YUCCA10-MBP, *At*YUCCA10-MBP or *Ca*YUCCA10-MBP were incubated with 1 mM NADPH and 100 µM IPA **15** for 20 min at 30°C and 600 rpm in buffer A. For *Ca*YUCCA10, FAD was added as co-factor to a final concentration of 30 µM. The reaction was quenched with 100 µL MeOH, centrifuged for 5 min at full speed in a table top centrifuge and 30 µL of supernatant were subjected to HPLC-DAD analysis. 2 µL of the sample were further analyzed by UHPLC-Orbitrap as described in the HPLC and LC-MS analysis section. Control reactions were performed with the enzymes denatured at 98°C for 10 min to ensure conversion by enzymatic activity. Figure S11 and S12 show the LC-MS data for confirmation of IAA **16** production and control reactions for *Vv*YUCCA10-MBP. Figure S29 and S30 shows HPLC-DAD data and calculated product yields for confirmation of IAA **16** production and control reactions for *At*YUCCA10-MBP and *Ca*YUCCA10-MBP. All experiments were carried out in triplicates. Area under the curves were calculated based on HPLC-DAD analysis with the LabSolutions software provided by Shimadzu.

**^18^O_2_ gas conversion experiment**

To study the incorporation of ^18^O-labelled oxygen into DPleBs, two stock solution were prepared separately and flushed for 5 min with argon gas. Stock solution 1 contained 1 mM NADPH in 25 mM buffer A. Stock solution 2 contained 2.56 µM *Vv*YUCCA10-MBP and 53 µM *Po*-PleB **1*n*** stock (*Po*-PleB **1*n*** dissolved in 1:1 MeOH:MilliQ at 3.1 mM) in buffer A in a total volume of 30 µL. After flushing both solutions in air-tight darkened vials with a septum with argon gas, 50 µL of stock solution 1 containing 1 mM NADPH was injected into stock solution 2 with a syringe and mixed. The reaction was allowed to react for 20 min at 28°C and 160 rpm. Then, 80 µL of MeOH were used to quench the reaction, the solution was centrifuged for 10 min at full speed with a table top centrifuge and 3 µL were injected into an UHPLC-Orbitrap system described in the HPLC and LC-MS analysis section. Figure S9 and Figure S10 show the mass distribution and MS^2^ fragments for ^18^O-labelled *Po*-PleB **1*n*** in positive and negative ion mode, respectively. The experiment was carried out in one replicate.

**Conversion of various substrate analogues with *Vv*YUCCA10-MBP**

For conversion reaction with other substrates than Vv-PleB-57 **1** and IPA **16**, a fresh 10 mM stock of NADPH was prepared in buffer A. The substrates *Cj*-PleB-2 **19**, *Cj*-PxB-2 **6**, *Cj*-PrB **7**, *At*-PleB-1 **21** and *Ob*-PleB-40 **22** were prepared as 3 mM stocks in 1:1 H_2_O:MeOH. Phenyl pyruvic acid (PPA) was prepared as 10 mM stock from sodium phenyl pyruvate (ThermoScientific, USA) in 1:4 MeOH:buffer A. 5 µM enzyme were incubated with 1 mM NADPH and 200 µM of substrate for 20 min at 30°C and 600 rpm in the dark in buffer A. The reaction was quenched with 10 µL MeOH, centrifuged for 5 min at full speed in a table top centrifuge and 30 µL of supernatant subjected to HPLC-DAD analysis. If the conversion reaction showed a new peak in the DAD analysis, 2 µL of the sample were further analyzed by UHPLC-Orbitrap as described in the LC-MS and HPLC section. Control reactions were performed with enzyme denatured at 98°C for 10 min to ensure conversion by enzymatic activity. Figure S18, S20, S22 show the HPLC traces for the conversion reactions and the control reactions at 254 nm for *Cj*-PleB-2 **19**, *Cj*-PxB-2 **6** and *Cj*-PrB **7** with *Vv*YUCCA10-MBP. Figure S19, S21 and S23 show the UHPLC-Orbitrap data and MS^2^ fragments for substrate and products of the conversion reactions and of *Cj*-PleB-2 **19**, *Cj*-PxB-2 **6** and *Cj*-PrB **7** with *Vv*YUCCA10-MBP. Figure S24 shows the result for the conversion experiment of PPA as a substrate with *Vv*YUCCA10. Area under the curves were calculated based on HPLC-DAD analysis with the LabSolutions software provided by Shimadzu. The experiment was carried out in triplicates.

**Inhibition assay of IPA 15 conversion with *Vv*-DPleB-51 8**

For confirmation of the inhibitory effect of *Vv*-DPleB-51 **8** on the conversion of IPA **15** to IAA **16** by *Vv*YUCCA10-MBP, a fresh 10 mM stock of NADPH was prepared in buffer A. A 3 mM stock of *Vv*-DPleB-51 **8** was prepared in 1:1 MilliQ:MeOH. IPA was prepared as a fresh 10 mM stock in MeOH and subsequently diluted to 1 mM by buffer A. For 3-fold excess of *Vv*-DPleB-51 **8** towards the IPA **15** substrate, 5 µM *Vv*YUCCA10-MBP was incubated with 1 mM NADPH and 300 µM of *Vv*-DPleB-51 **8** for 5 min at 30°C and 600 rpm in the dark in buffer A. Then, IPA was added to give a final concentration of 100 µM in a total volume of 50 µL and the incubation proceeded for 20 min at 30°C and 600 rpm in the dark. The reaction was quenched with 10 µL MeOH, centrifuged for 5 min at full speed in a table top centrifuge and 30 µL of supernatant subjected to LC-MS or 2 µL to HR-UHPLC-Orbitrap analysis. For 10-fold excess of *Vv*-DPleB-51 **8** towards the IPA **15** substrate, 5 µM *Vv*YUCCA10-MBP was incubated with 1 mM NADPH and 1 mM *Vv*-DPleB-51 **8** for 5 min at 30°C and 600 rpm in the dark in buffer A. Then, IPA was added to give a final concentration of 100 µM in a total volume of 10 µL and the incubation proceeded for 20 min at 30°C and 600 rpm in the dark. The reaction was quenched with 10 µL MeOH, centrifuged for 5 min at full speed in a table top centrifuge and 2 µL to HR-UHPLC-Orbitrap analysis. Area under the curves were calculated based on XICs created with ACD/Labs software. The experiment was carried out in triplicates.

**Competitive assay with *Vv*-PleB-57 1 and *Vv*-PxB (*Cj*-PxB-2) 6**

For competition experiment with 1:1 *Vv*-PleB-57 **1** and *Vv*-PxB (*Cj*-PxB-2) **6**, a fresh 10 mM stock of NADPH was prepared in buffer A. A 3 mM stock of Vv-PleB-57 **1** or Vv-PxB **6** was prepared in 1:1 MilliQ:MeOH. 5 µM *Vv*YUCCA10-MBP was incubated with 1 mM NADPH, 50 µM *Vv*-PleB-57 **1** and 50 µM *Vv*-PxB (*Cj*-PxB-2) **6** for 20 min at 30°C and 600 rpm in a total volume of 50 µL in the dark. The reaction was quenched with 50 µL of MeOH, centrifuged for 5 min at full speed in a table top centrifuge and 30 µL subjected to HPLC-DAD analysis. The experiment was carried out in four replicates. Area under the curves were determined at 218 nm for the reaction of **1** to **8** and at 425 nm for **6** to **9** with LabSolutions software provided by Shimadzu.

***Po*-PleB 1*n* conversion with 10-fold excess of IAA 16**

For determine, if IAA **16** has an inhibitory effect on the deformylation reaction catalyzed by *Vv*YUCCA10-MBP*,* a fresh 10 mM stock of NADPH was prepared in buffer A. A 1 mM stock of IAA **16** was prepared in buffer A. *Po*-PleB **1*n*** was prepared as 3.1 mM stock in 1:1 MilliQ:MeOH in a dark Eppendorf tube. 5 µM *Vv*YUCCA10-MBP were incubated with 1 mM NADPH and 500 µM of IAA **16** for 5 min at 30°C and 600 rpm in the dark in buffer A. Then, *Po*-PleB **1*n*** was added to give a final concentration of 50 µM and the incubation proceeded for 20 min at 30°C and 600 rpm in the dark to a total volume of 50 µL. The reaction was quenched with 50 µL of MeOH, centrifuged for 5 min at full speed in a table top centrifuge, and a 2 µL aliquot was used for HR-UHPLC-Orbitrap analysis. The experiment was carried out in two replicates. Figure S17 shows XIC traces for IAA **16**, *Po*-PleB **1*n*** and *Po*-DPleB **8*n*** exemplified by one replicate.

**Conversion assay of cyclohexanone** **with *Vv*YUCCA10-MBP**

To test potential conversion of cyclohexanone to ε-caprolactone by *Vv*YUCCA10-MBP, the conversion reaction was upscaled to 30 µM *Vv*YUCCA10-MBP which was incubated with 60 mM cyclohexanone and 80 mM NADPH in buffer A in an Eppendorf tube in a total volume of 25 µL. NADPH was prepared as 1M stock in buffer A and cyclohexanone was added directly without any stock preparation. The reaction was started by the addition of the substrate cyclohexanone and incubated for 1h at 30°C and 600 rpm. The reaction was quenched by the addition of 100 µL of hexane, vigorous shaking and centrifugation for 5 min at full speed in a table top centrifuge. 25 µL were transferred to a GC-vial and 1 µL injected into the GC-MS system described in the GC-MS analysis section. Figure S24 shows the XIC traces for substrate cyclohexanone and product m/z of the conversion experiment.

**Identification and quantification of *in vivo* amounts of Chl catabolites and auxin metabolites in grapevine leaves**

For metabolomic studies on Chl catabolites and auxin metabolites in grapevine leaves, yellow-green grapevine leaves stored at -80°C were ground in liquid nitrogen and 500µL of MeOH and 300 µL of 50 mM sodium phosphate buffer, pH 5.6 were added. The resulting slurry was shaken gently for 15 min on ice and 400 µL of 0.1 N HCl was added to change the pH to around 2. The slurry was centrifuged for 10 min at top speed with a table top centrifuge and the supernatant sterile filtered with a 0.45 µM syringe filter. 10 µL of the supernatant was diluted with 20 µL of MeOH.

In an initial experiment, the IAA **16** detection limit was determined by spiking of IAA as a freshly prepared stock into grapevine leave lysates and measurement with 2 µL injection volume with an HR-MS Orbitrap as described in the LC-MS and HPLC section. For the spiked concentrations, a range between 100 pg/mL down to 0.1 pg/ml was chosen under the assumption that an extract of 1 g of fresh plant material was solubilized in 50 µL of solvent at an IAA concentration as determined by Yamamoto *et al.* of 20 pg/gfw IAA **16** in rice.^49^ The *in vivo* concentration of IAA would correspond based on these calculations to around 0.4 pg/mL of IAA. A 1 mg/mL stock of IAA was prepared in MeOH and diluted to 10 µg/mL with MilliQ. This stock was used to add a final concentration of 100 pg/mL into 30 µL of grapevine lysate. For smaller concentrations like 50 pg/mL and 10 pg/mL, a stock concentration of 1 µg/mL was used to add IAA **16** into 30 µL of grapevine lysate. For 5 pg/m and 1 pg/mL end concentration, a stock concentration of 500 pg/mL and 100 pg/mL was used. IAA **16** eluted at 6.89 min with the HR-UHPLC-Orbitrap method described in the LC-MS and HPLC section. XICs were generated with ACD/Labs software and evaluated by their counts of the area under the curves. The experiment was conducted as one replicate and is shown in Figure S15.

For determination of Chl catabolites and auxin metabolites, 2 µL of grapevine lysate were injected into the HR-UHPLC-Orbitrap as described above. Chl catabolites were identified by generation of their respective XICs. The experiment was conducted in triplicates with different leaf batches. Figure S13 shows the HPLC-DAD chromatogram at 254 nm for the vine leaf extract as well as XICs of detected Chl catabolites and standards auf IPA **15** and IAA **16**. MS^2^ data for the detected Chl catabolites *Vv*-DPleB-57 **8** and *Vv*-DPxB-63 **9** is shown in Figure S14.

**Identification and quantification of *in vivo* amounts of Chl catabolites and auxin metabolites in *A. thaliana* siliques and *Coffea arabica* leaves**

Siliques of *A. thaliana* were separated into green, yellow, and brown developmental stages. Samples of 18.76 mg (green), 15.34 mg (yellow), and 10.76 mg (brown) were weighed and homogenized in a mortar with 700 µL of MeOH/H₂O (80:20, v/v). The resulting extracts were centrifuged at 13,000 rpm, and the supernatants were filtered through 0.2 µm PTFE membrane filters (Phenomenex, Aschaffenburg, Germany). Aliquots of each extract were used for the analysis of phyllobilins and Trp metabolites with HR-UHPLC-Orbitrap as described in the LC-MS and HPLC section. Figure S16 shows relative peak areas of phyllobilins and Trp metabolites.

Coffee leaves at different stages of senescence were collected from a commercially purchased plant. The leaves were lyophilized and ground to a fine powder. A 10 mg portion of the powder was extracted with 1 mL of MeOH/H₂O (80:20, v/v) by sonication for 15 minutes. The resulting extracts were centrifuged at 13,000 rpm, the supernatants were filtered through 0.2 µm PTFE membrane filters, and stored at -80°C if not analyzed immediately. Aliquots of each extract were used for the analysis of phyllobilins and tryptophan metabolites with HR-UHPLC-Orbitrap as described in the LC-MS and HPLC section. Figure S31 shows the HPLC-DAD chromatogram at 254 nm for the coffee leaf extract as well as XICs of detected Chl catabolites, UV spectra, chemical structures and their MS^2^ fragments. Figure S32 shows relative peak areas of the detected phyllobilins.

**Extraction of *Vv*-DPleB-51 and other chlorophyll catabolites**

*Vv*-DPleB-51 was kindly provided by Bernhard Kräutler (University of Innsbruck). *Ob*-PleB-40 was isolated from senescent basil (*Ocimum basilicum*) leaves, *At*-PleB-1 from senescent *Arabidopsis thaliana* leaves, while *Cj*-PleB-2, *Cj*-PxB-2, and *Cj*-PrB were obtained from senescent *Cercidiphyllum japonicum* leaves following published protocols.^7,11,17,68-70^ All used Chl catabolites have been fully characterized by NMR and clearly assigned to either the ‘*epi*’ or ‘*n*’ series of catabolites by comparison of retention times and standards (Table S4). The *Vv*-DPleB-51 stock was further purified on semi-preparative HPLC as stated in the LC-MS and HPLC section and lyophilized. With the described method, *Vv*-DPleB-51 eluted at 18.6 min. Purity of the compound was confirmed by UHPLC-Orbitrap analysis. Note: all Chl catabolites were instable and purification to 100% purity could not be achieved for all stocks.

**Statistics**

Statistics were assessed with Origin2023 software and a two-sample t-test for all replicates under the assumption of equal variance. Values that were significantly different were marked by bars and asterisks according to the GraphPad Prism 6 statistics guidelines with ****: P<0.0001.***: P<0.0001-0.001, **:P<0.001-0.01 and *:P<0.01-0.05.

# **Table S1** **Chlorophyll degradation genes and corresponding enzymes of *Vitis vinifera*.**

For determination of co-expressed genes with the known catabolite enzymes from chlorophyll catabolism steps, the ATTED network drawer (https://atted.jp/top_draw/#CoexViewer, retrieved on 02.01.2024) was used and fed with the listed genes from *Vitis vinifera.*

| Gene (NCBI accession #) | gene name (NCBI) | Protein UniProt ID |
| --- | --- | --- |
| 100265442 | LOC100265442 pheophytinase, chloroplastic [ Vitis vinifera (grapevine)] | F6HHX5 |
| 100256831 | LOC100256831 pheophorbide a oxygenase, chloroplastic [ Vitis vinifera (grapevine)] | C1K6H0 |
| 100252439 | LOC100252439 red chlorophyll catabolite reductase [ Vitis vinifera (grapevine)] | D7SVY1 |
| 100247370 | LOC100247370 ethylene-responsive transcription factor ERF017 [ Vitis vinifera (grapevine)] | F6HGN0 |
| 100265112 | LOC100265112 protein STAY-GREEN homolog, chloroplastic [ Vitis vinifera (grapevine)] | F6HUJ1 |
| 100252353 | LOC100252353 magnesium dechelatase SGRL, chloroplastic [ Vitis vinifera (grapevine)] | E0CQY6 |
| 100259354 | LOC100259354 protein TIC 55, chloroplastic [ Vitis vinifera (grapevine)] | D7SV72 |
| 100265035 | LOC100265035 chlorophyll(ide) b reductase NOL, chloroplastic [ Vitis vinifera (grapevine)] | D7TA80 |

# **Table S2 Enzymes used for calculation of phylogeny with MEGA11.^67^**

| Gene (NCBI accession #) | gene name (tree) | Protein (NCBI accession #) | Organism |
| --- | --- | --- | --- |
| AT4G32540 | AtYUCCA1 | Q9SZY8.1 | *A. thaliana* |
| AT4G13260 | AtYUCCA2 | Q9SVQ1.1 | *A. thaliana* |
| AT1G04610 | AtYUCCA3 | NP_171955.1 | *A. thaliana* |
| AT5G11320 | AtYUCCA4 | Q9LFM5.1 | *A. thaliana* |
| AT5G43890 | AtYUCCA5 | ABM06013.1 | *A. thaliana* |
| AT5G25620 | AtYUCCA6 | Q8VZ59.1 | *A. thaliana* |
| AT2G33230 | AtYUCCA7 | O49312.1 | *A. thaliana* |
| AT4G28720 | AtYUCCA8 | ABJ98570.1 | *A. thaliana* |
| AT1G04180 | AtYUCCA9 | O64489.1 | *A. thaliana* |
| AT1G48910 | AtYUCCA10 | NP_175321.1 | *A. thaliana* |
| AT1G21430 | AtYUCCA11 | Q9LPL3.1 | *A. thaliana* |
| Z47553.1 (mRNA) | flavin-containing monooxygenase 5 (FMO5) (liver FMO) | CAA87633.1 | *Homo sapiens* |
| AT1G19250 | FMO1 flavin-dependent monooxygenase 1 (At FMO1) | NP_173359.3 | *A. thaliana* |
| 882167 | L-ornithine N5-oxygenase  (Pa SID OXY) | NP_251076.1 | *Pseudomonas aeruginosa* |
| no id | Chain A, Dimethylaniline monooxygenase, putative (FUNGALFORMATE) | KAF7625214.1 | *Aspergillus flavus* NRRL3357 |
| 113734406 | CaYUCCA10 | XP_027116742.1 | *Coffea arabica* |
| 100245859 | VvYUCCA10 | XP_002269844.1 | *Vitis vinifera* |

# **Table S3 Localization prediction by TMHMM and Target P.2.0.**

| Enzyme | TMD predicted by TMHMM 2.0 (position; N-terminus) | Targeting sequences by TargetP 2.0 | Localization | Gene ID, mRNA transcript, length, localization |
| --- | --- | --- | --- | --- |
| *Vv*YUCCA10  (VIT_00011006001) | 22-42 | None  Other | Vacuole^43^ | 100245859, XM_002269808.5,  393 aa,  Berries^43^ |
| *At*YUCCA10 | 0 | None  Cytosol | Cytosol^32^ | AT1G48910.1,  NM_103784.2,  383 aa,  Pollen^32^ |
| *Ca*YUCCA10 | 7-29 | None | unknown | 113734406, XM_027260941.2, 385 aa |

# **Table S4 Overview on used chlorophyll catabolites in this manuscript and their published NMR data.**

| Chlorophyll catabolite | Plant species | Reference for NMR data |
| --- | --- | --- |
| *Po*-PleB 1*n* | *Platanus occidentalis* | 11 |
| *Cj*-PleB (*Vv*-PleB-57) 1 | *Cercidiphyllum japonicum* | 53 |
| *Cj*-PxB-2 (*Vv*-PxB) 6  *Cj*-PrB 7 | *Cercidiphyllum japonicum* | 57 |
| *Cj*-PleB-2 19 | *Cercidiphyllum japonicum* | 7 |
| *Vv*-DPxB-63 9 | *Vitis vinifera* | 16 |
| *Vv*-DPleB-51 8 | *Vitis vinifera* | 70 |
| *At*-PleB-1 21 | *Arabidopsis thaliana* | 69 |
| *Ob*-PleB-40 22 | *Ocimum basilicum* | 68 |


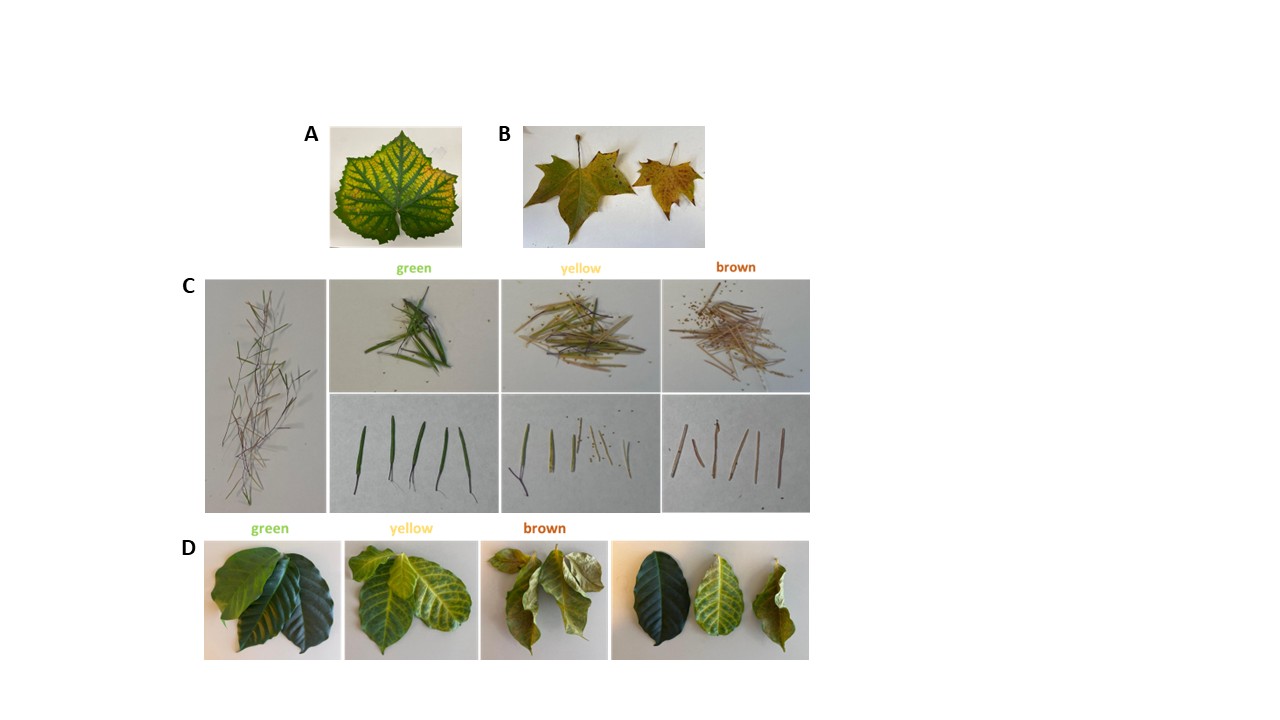


# **Figure S1 Plant Material used for this study.**

**A** Yellow-green grapevine leaves (*Vitis vinifera*) collected for pull down assay and metabolomics studies. **B** Plane tree leaves (*Platanus* *occidentalis*) were collected to extract *Po*-PleB. **C** *Arabidopsis thaliana* siliques to study Chl catabolites and Trp metabolites were collected from plants grown in the Botanical Garden of Innsbruck, Austria. **D** *Coffea arabica* leaves to study Chl catabolites were obtained from a commercially purchased plant


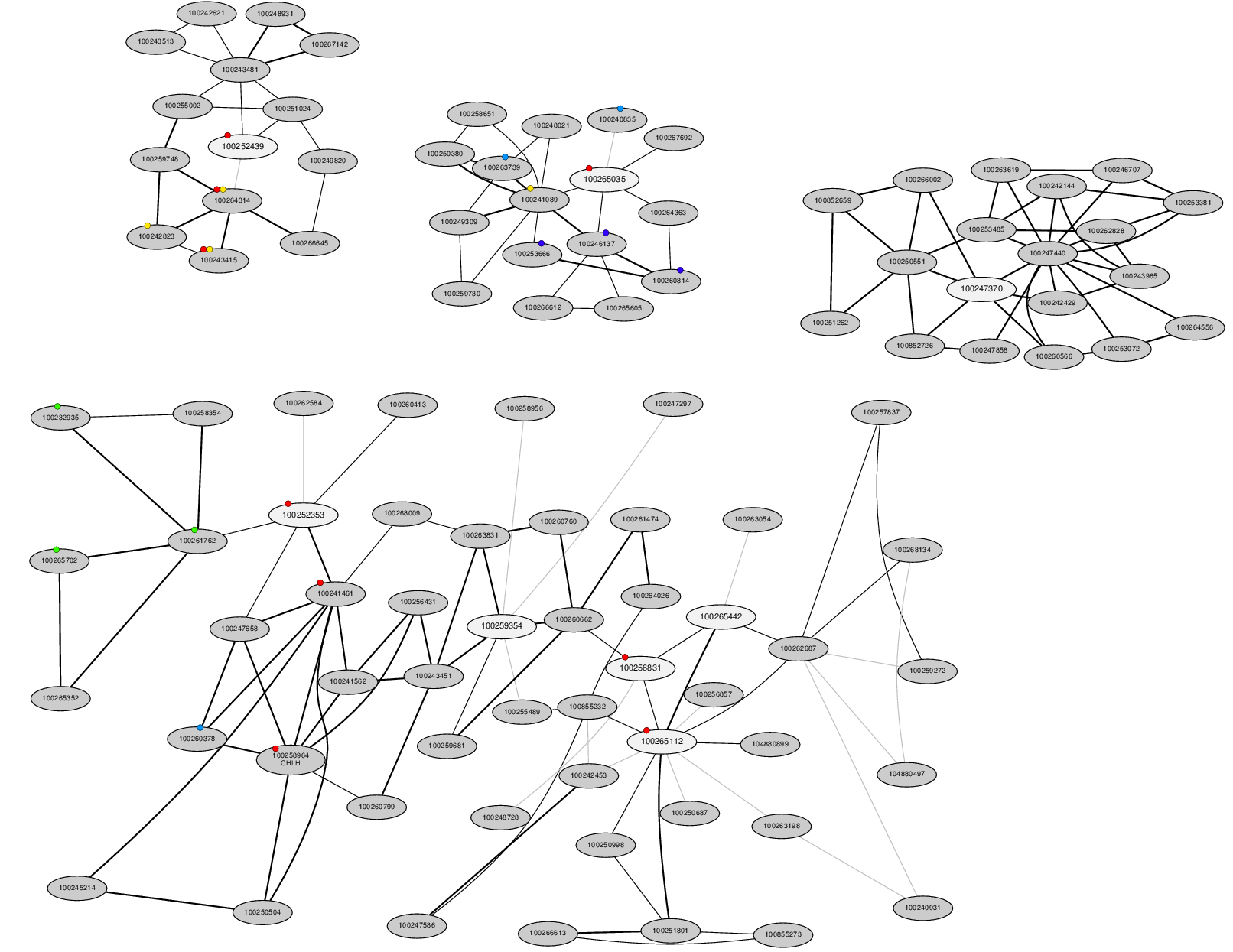


# **Figure S2** **Co-expression network generated by the ATTED network drawer.**

The ATTED network drawer was fed with the *Vitis vinifera* genes involved in chlorophyll catabolism (retrieved in January 2025). Network was drawn as GraphVix version.


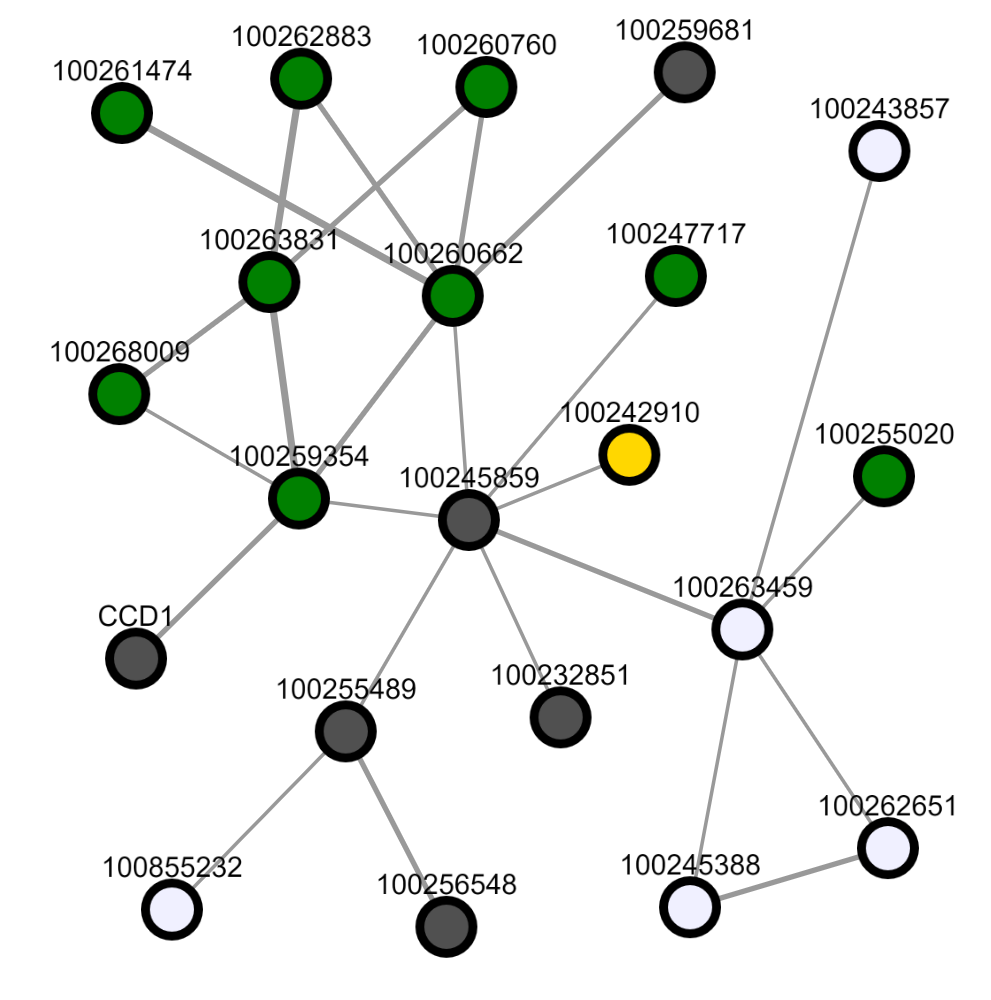


# **Figure S3 Co-expression network generated by the ATTED network drawer with the *vvyucca10* gene as bait.**

*vvyucca10* (100245859, black box) gene was used as a bait. The generated network (retrieved in January 2025) was drawn as Cytoscape version. 100259354 encodes the gene *tic55* (translocon at the inner chloroplast envelope) which is involved in chlorophyll catabolism. The colors indicate the sub-cellular location predicted by the TargetP tool integrated into ATTED-II: green: chloroplast, grey: nucleus, white: secretory, yellow: mitochondrial.

**
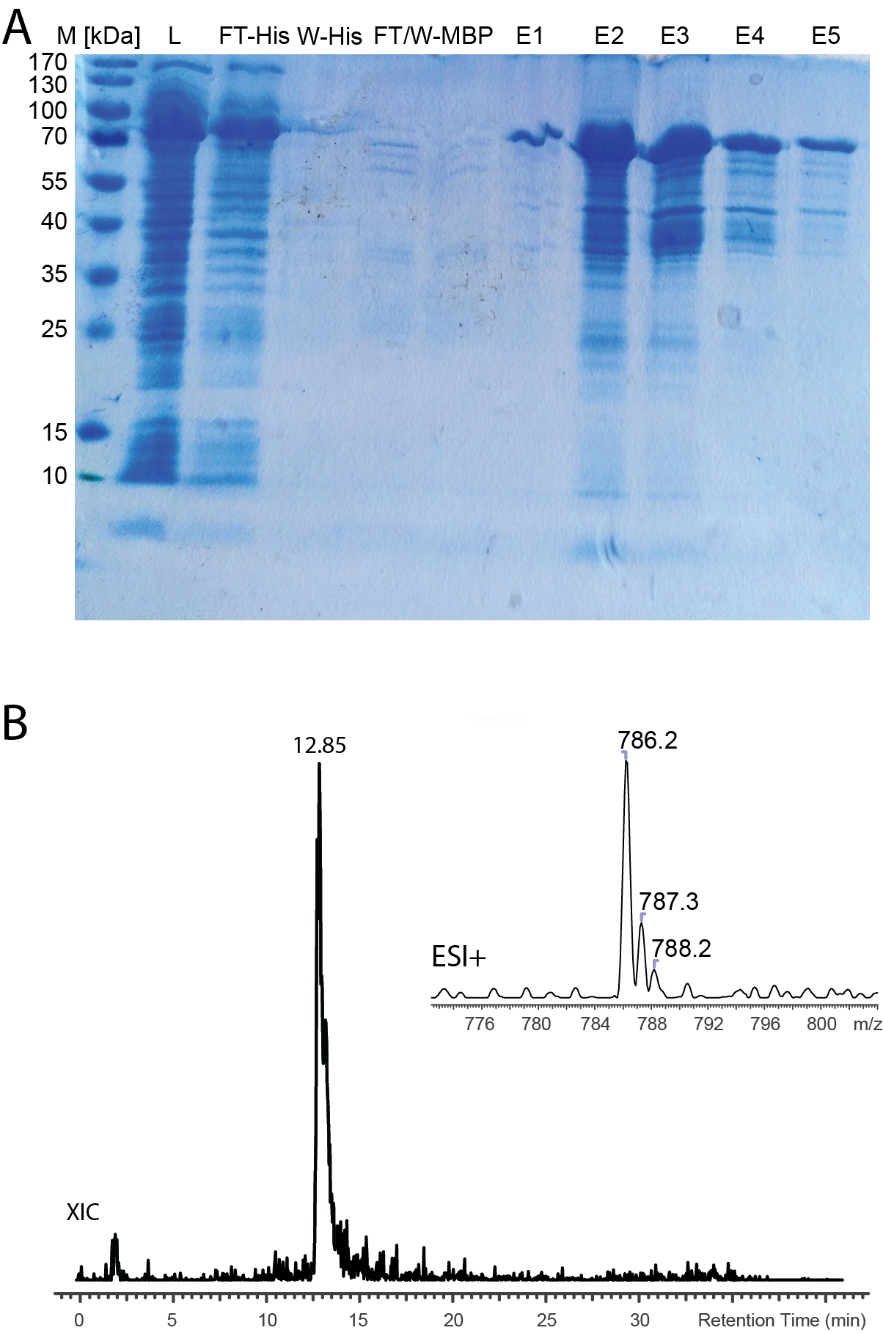
**

# **Figure S4 SDS-Page of purified *Vv*YUCCA10-MBP and cofactor determination.**

**A** SDS-PAGE analysis of different fractions collected during affinity purification (IMAC) of MBP-tagged *Vv*YUCCA10 with an expected molecular weight of 86.6 kDa. M: Marker, PageRuler (Prestained protein ladder, ThermoScientific), L: cleared *E. coli* lysate. FT-His and W-His: Flow-through and wash (20 mM imidazole) collected during the load of the IMAC His-column. FT/W-MBP: Flow-through and wash collected during the load of the MBP-column. E1-E5: Fraction containing *Vv*YUCCA10-MBP after MBP-trap purification. **B** Extracted FAD cofactor from *Vv*YUCCA10-MBP measured with low-resolution LC-MS in positive ion mode. XIC for FAD at m/z = 786.2 [M+H]^+^ is shown. **Insert:** Isotopic pattern of m/z = 786.2 [M+H]^+^ of FAD cofactor.


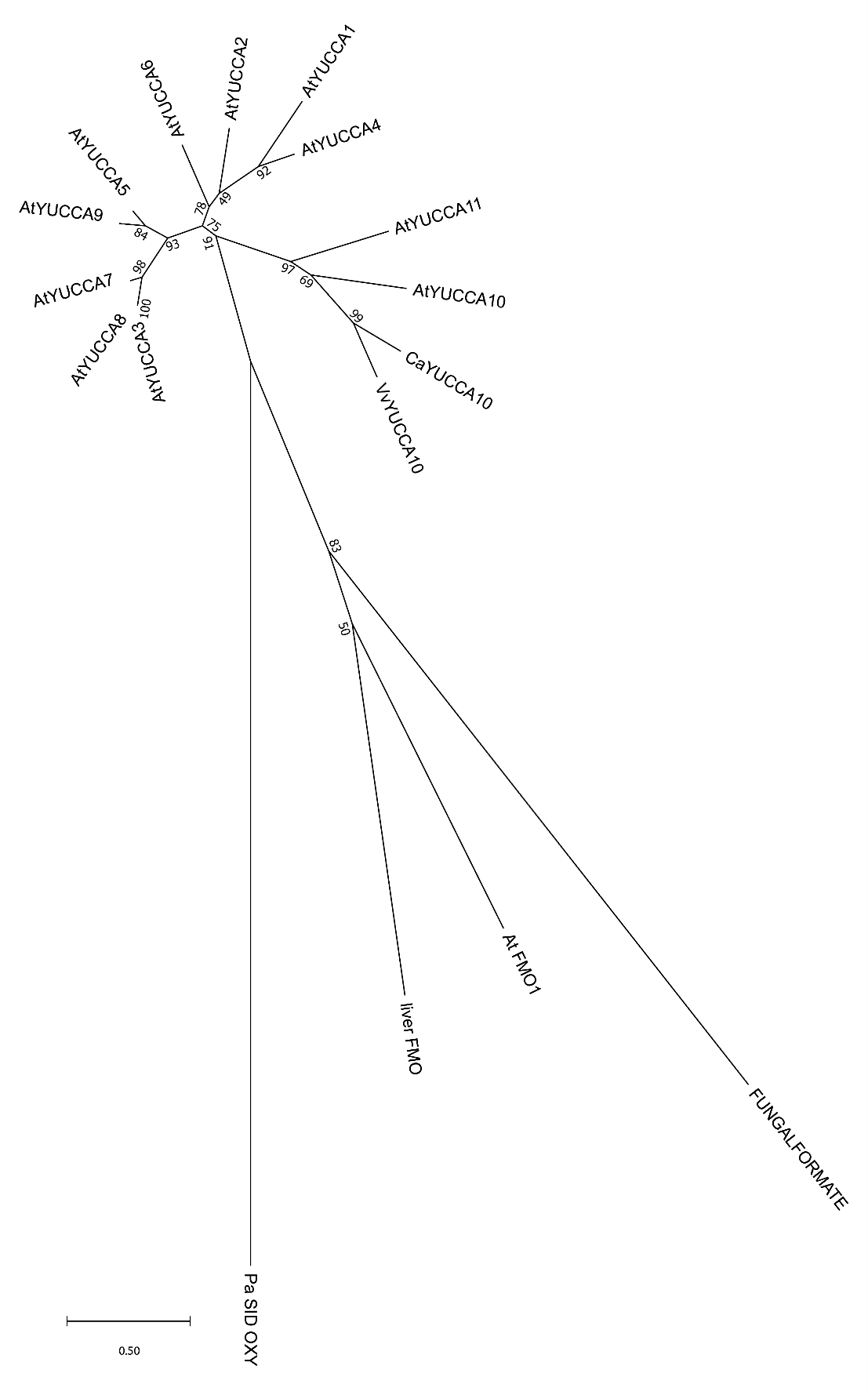


# **Figure S5 Unrooted phylogenetic tree based on the maximum likelihood model with LG and G.**

The tree was generated with MEGA11^67^ and Bootstrap frequencies (as percentage of 1000 iterations) are shown. AtFMO1: NCBI accession # NP_173359.3, liver FMO: NCBI accession # CAA87633.1, FUNGALFORMATE: NCBI accession # KAF7625214.1, Pa SID OXY: NCBI accession # NP_251076.1. Further information on protein sequences and origin is described in the phylogenetic tree section above.


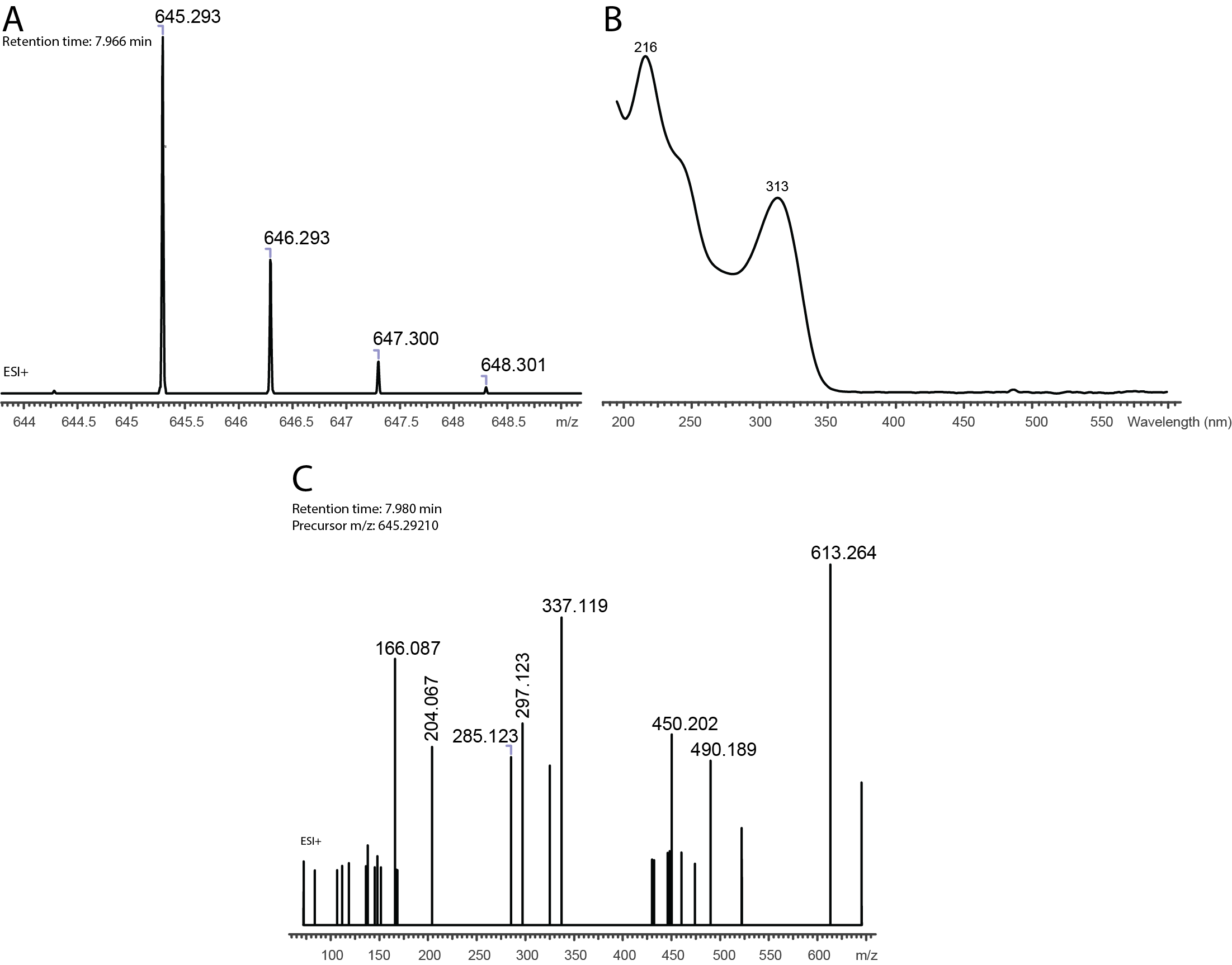


# **Figure S6 Identification of *Po*-PleB 1*n* by HR-MS^2^ and UV.**

**A** HR-MS of *Po*-PleB **1*n*** with C_35_H_40_N_4_O_8_ m/z = 645.293 [M+H]^+^ (calc. 645.29189, Δ 1.7 ppm). **B** UV-spectra of *Po*-PleB **1*n***. **C** MS^2^ HR fragments of *Po*-PleB **1*n***.


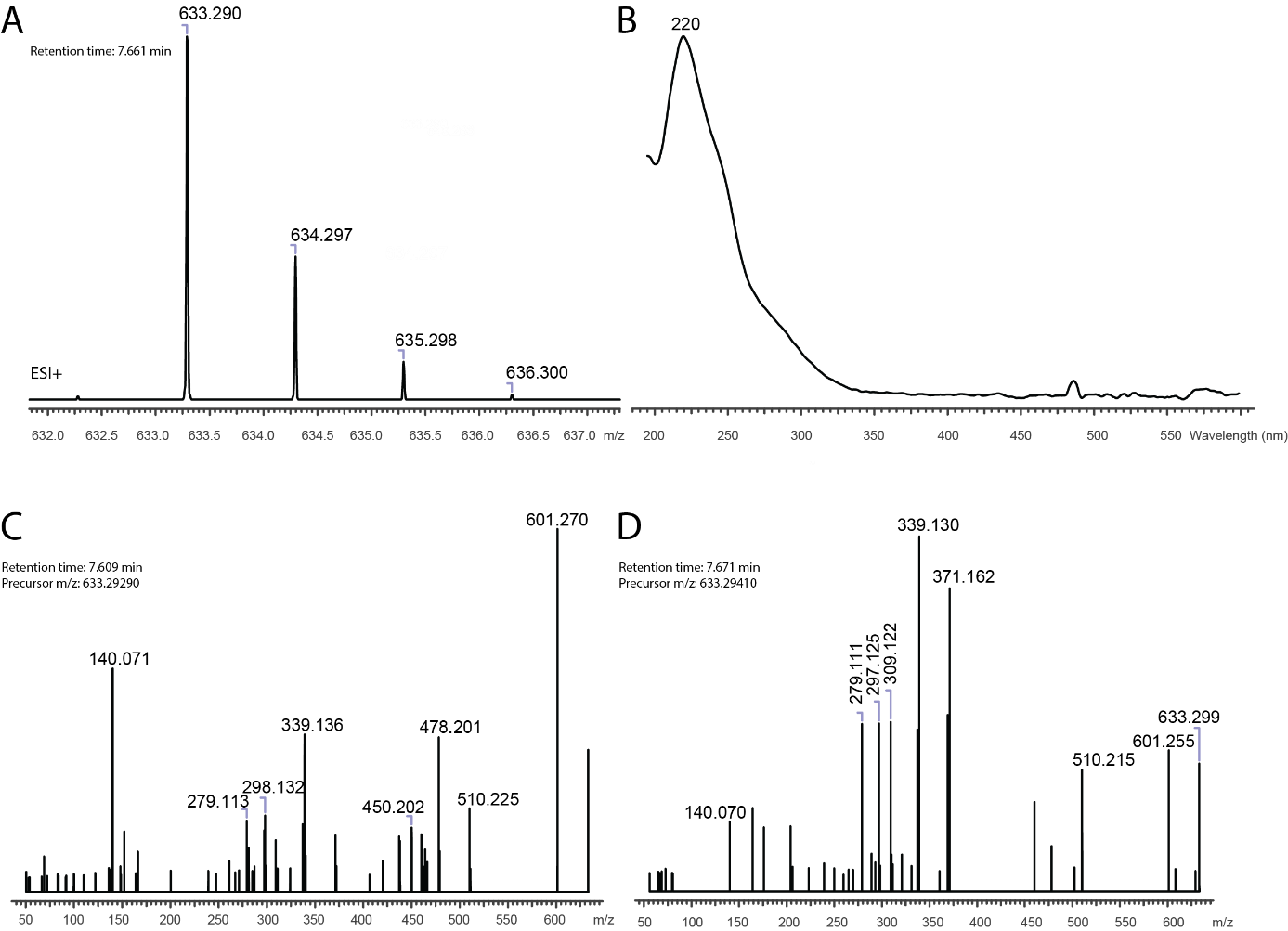


# **Figure S7** **Identification of *Po*-DPleB 8*n* by HR-MS^2^ and UV.**

**A** HR-MS of *Po*-DPleB **8*n*** with C_34_H_40_N_4_O_8_ m/z = 633.294 [M+H]^+^ (calc. 633.29189, Δ 3.3 ppm). **B** UV-spectra of *Po*-DPleB **8*n***. **C** MS^2^ HR fragments of *Po*-DPleB **8*n***. **D** MS^2^ HR fragments of *Vv*-DPleB-51 **8** standard.


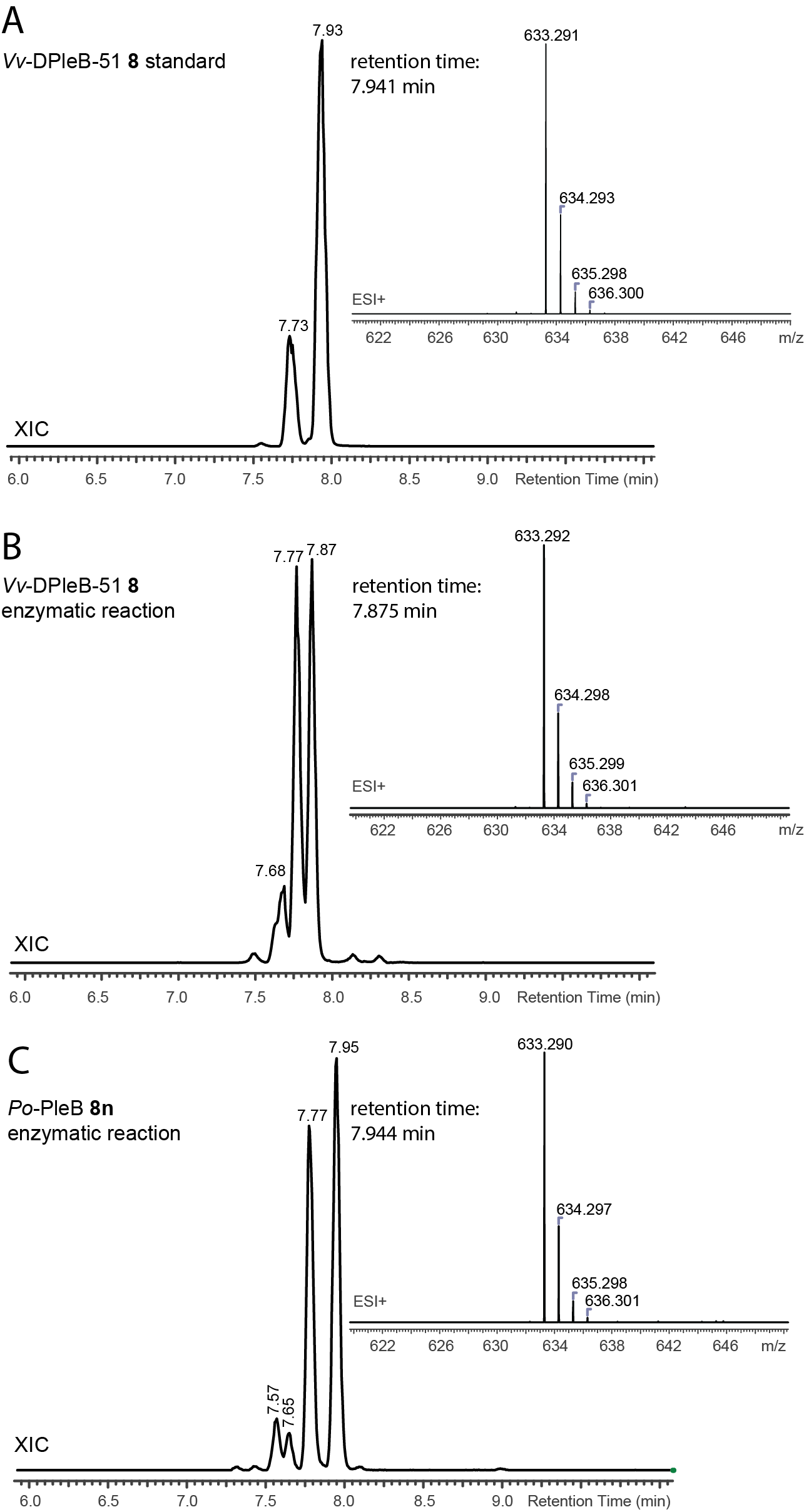


# **Figure S8 Comparison of *Po*-DPleB 8*n* and *Vv*-DPleB-51 8 by HR-MS in positive ion mode to an isolated standard of *Vv*-DPleB-51 8.**

**A** XIC of *Vv*-DPleB-51 **8** with C_34_H_40_N_4_O_8_ m/z = 633.291 [M+H]^+^ isolated from grapevine leaves (*Vitis vinifera*). **B** XIC of product *Vv*-DPleB-51 **8** with C_34_H_40_N_4_O_8_ m/z = 633.292 [M+H]^+^ from enzymatic conversion of *Vv*-PleB-57 **1** to **8** catalyzed by *Vv*YUCCA10-MBP. **C** XIC of *Po*-DPleB **8*n*** with C_34_H_40_N_4_O_8_ m/z = 633.290 [M+H]^+^ from enzymatic conversion of *Po*-PleB **1*n*** to **8*n*** catalyzed by *Vv*YUCCA10-MBP.


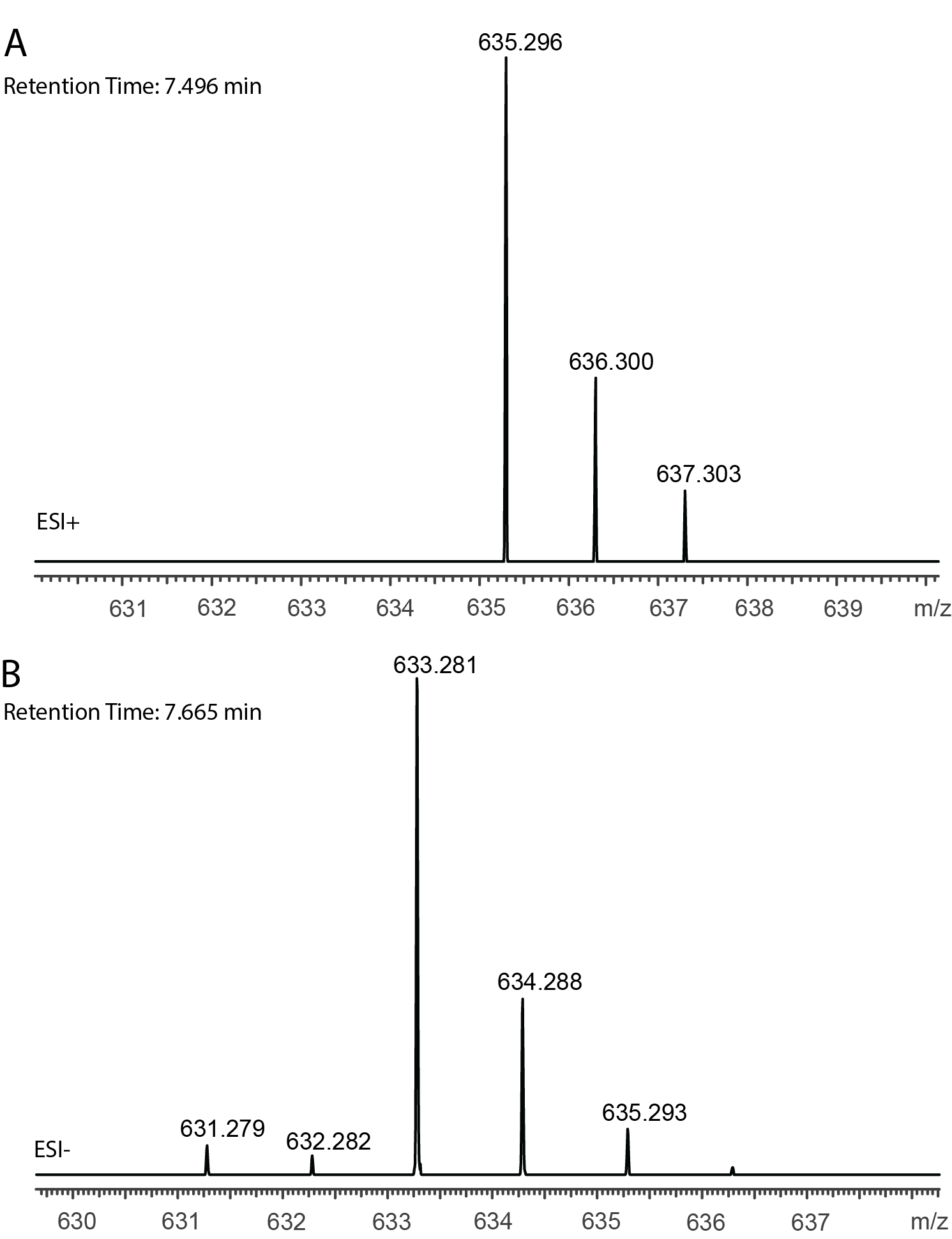


# **Figure S9** **Incorporation of ^18^O-labelled oxygen into *Po*-PleB 8*n*.**

**A** Distribution of masses for ^18^O-labelled *Po*-PleB **8*n*** in positive ion mode: m/z = 635.296 [M+H]^+^ (calc. 635.296137 [M+H]^+^, Δ 0 ppm). **B** Distribution of masses for ^18^O-labelled *Po*-PleB **8*n*** in negative ion mode. m/z = 633.281 [M-H]^-^ (calc. 633.281585 [M-H]^-^, Δ 0 ppm. The amount of labelled product was determined in negative ion mode to be 93%. In positive ion mode, no mass was detected for unlabeled **8**.


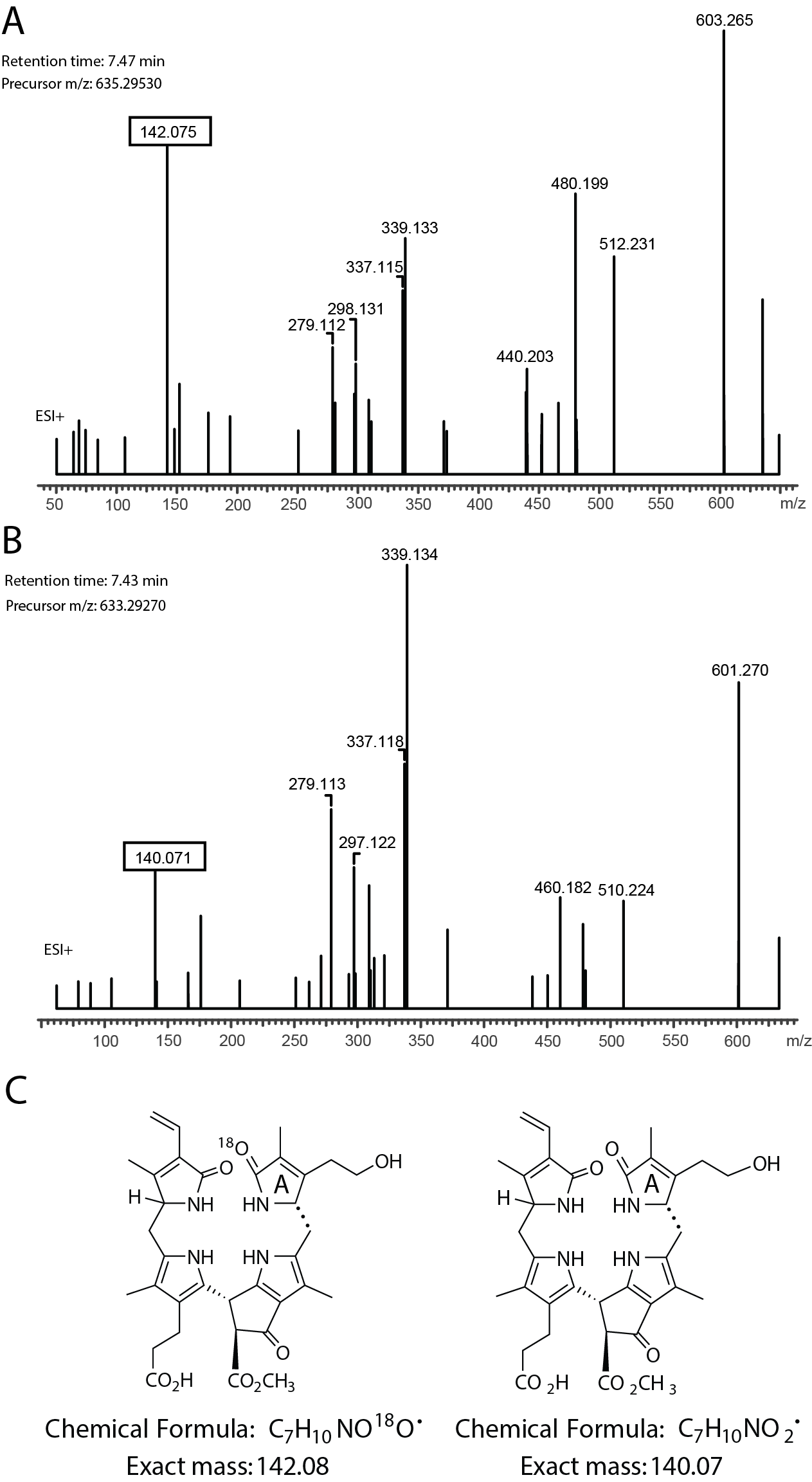


# **Figure S10** **Fragments of ^16^O-*Po*-DPleB and ^18^O-*Po*-DPleB.**

**A** MS^2^ HR fragments for ^18^O-labelled *Po*-DPleB in positive ion mode. The A-ring fragment at m/z = 142.075 (black box) is highlighted, which confirms incorporation of ^18^O into the A-ring of *Po*-DPleB **8*n***. **B** MS^2^ HR fragments for ^16^O-*Po*-DPleB **8*n*** in positive ion mode. The A-ring fragment m/z = 140.071 (black box) is highlighted for comparison to ^18^O-labelled A ring of *Po*-PleB **8*n***. **C** Structure comparison of ^16^O-labelled *Po*-DPleB and ^18^O-labelled *Po*-DPleB. Fragmentation patterns predicted with ChemDraw software define the A-ring fragment as site of incorporation of ^18^O into *Po*-PleB **8*n***.


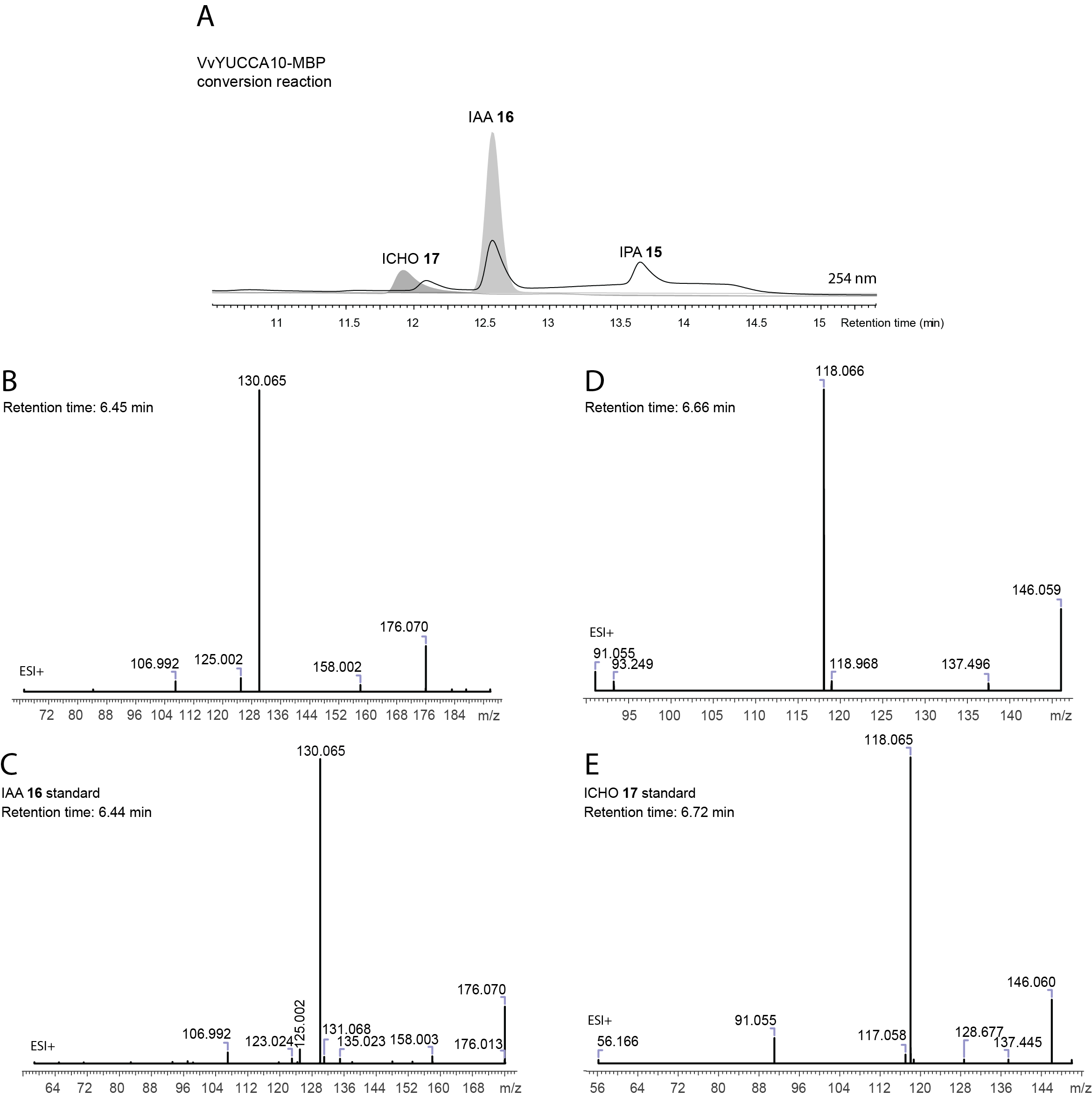


# **Figure S11** **IPA 15 conversion to IAA 16 by *Vv*YUCCA10-MBP.**

**A** HPLC-DAD chromatogram at 254 nm of the conversion reaction after 20 min at 30°C. IAA **16** elutes at 12.58 min. An IAA **16** and ICHO **17** commercial standard showed the same retention time (grey shadows). **B** MS^2^ fragments generated with an UHPLC-Orbitrap for IAA **16** produced by *Vv*YUCCA10-MBP. **C** MS^2^ fragments generated with an UHPLC-Orbitrap for a commercial IAA **16** standard. **D** MS^2^ fragments generated with an UHPLC-Orbitrap for ICHO **17** produced by *Vv*YUCCA10-MBP. **C** MS^2^ fragments generated with an UHPLC-Orbitrap for commercial ICHO **17** standard.


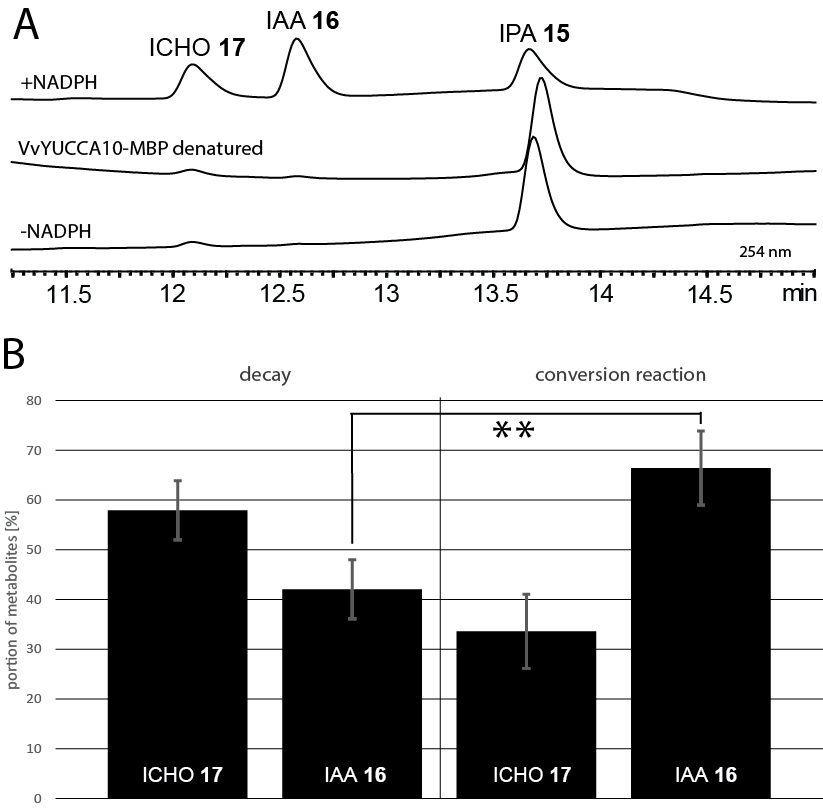


# **Figure S12** **Enzymatic conversion of IPA 15 to IAA 16 catalyzed by *Vv*YUCCA10 and area under the curves to confirm enzymatic activity.**

**A** Enzymatic IPA **15** conversion assays and control reactions without redox partner and with denatured enzyme *Vv*YUCCA10-MBP shown at 254 nm. **B** The bar chart shows the mean product yields of triplicates given in percent for indole-3-carbaldehyde (ICHO) **17** and indole-3-acetic acid (IAA) **16** for decayed IPA **15** and the enzymatic reaction catalyzed by *Vv*YUCCA10. Statistics were assessed by a two-sample t-test with equal variance assumption for triplicates. The product yields were determined by area under the curves (AUC) of XIC traces generated for **16** and **17** and subsequent calculation of product formation in percent as ratio of **16** to overall **16** and **17** formation. The error bars are shown as calculated standard deviations from the mean of 3 replicates. The statistical analysis resulted in a significant increase (P = 0.00944) of IAA **16** production for *Vv*YUCCA10. Values that were significantly different were marked by bars with ****: P<0.0001.***: P<0.0001-0.001, **:P<0.001-0.01 and *:P<0.01-0.05.


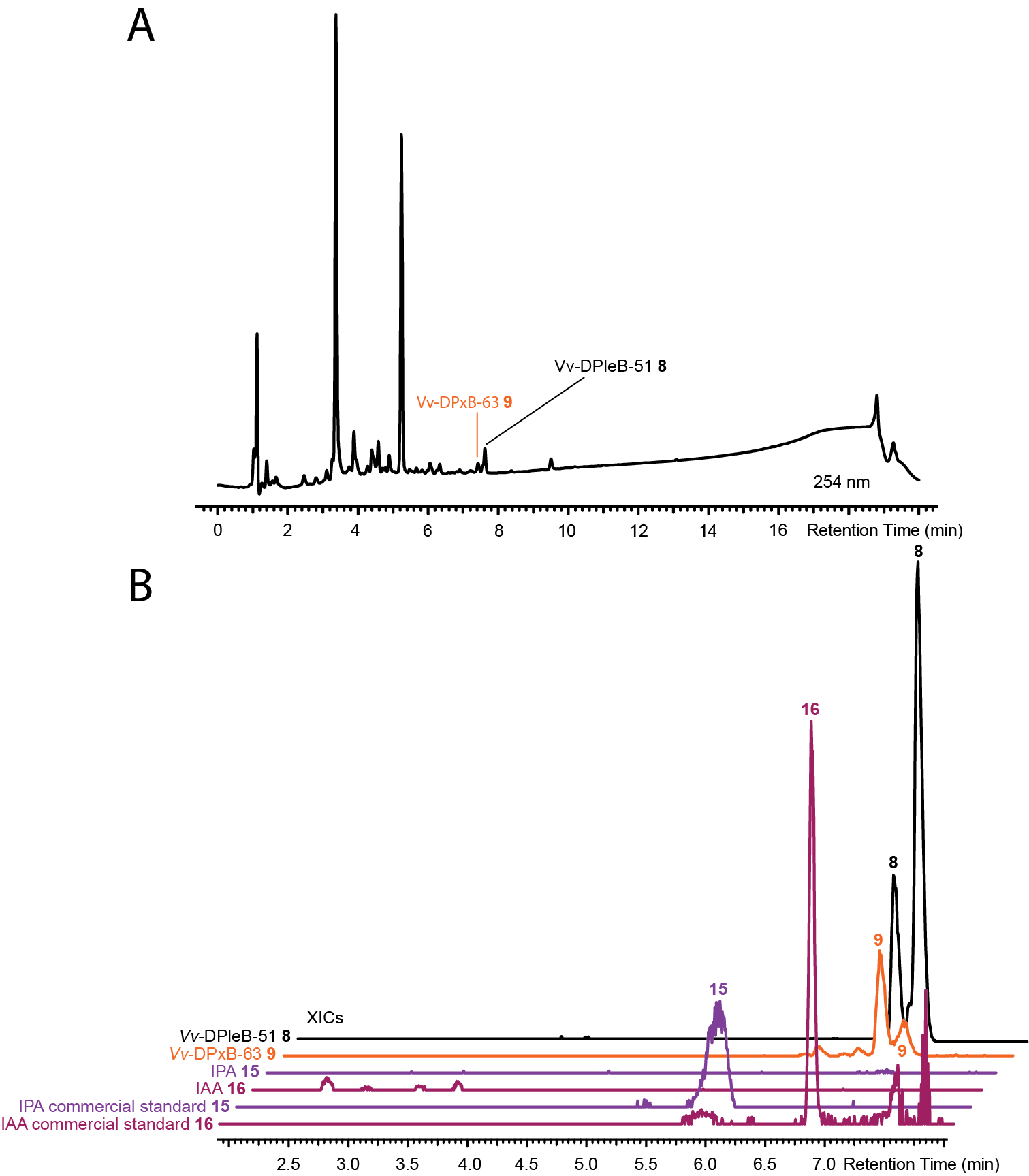


# **Figure S13** **HPLC-DAD chromatogram shown at 254 nm of lysate of yellow-green grapevine leaves and extracted ion chromatograms (XIC) shown for important chlorophyll (Chl) catabolites and auxin metabolites IPA 15 and IAA 16.**

**A** Overview of HPLC-DAD chromatogram of whole vine leaf lysate at 254 nm. **B** XIC traces for *Vv*-DPleB-51 **8** (black) at m/z = 633.29249 [M+H]^+^ and *Vv*-DPxB-63 **9** at m/z = 633.29249 [M+H]^+^ (orange)**.** Both Chl catabolites were unambiguously identified with HR-MS and HR-MS^2^ (Figure S14). XICs for IPA **15** at m/z = 204.0655 [M+H]^+^ (violet) and IAA **16** at m/z = 176.0706 [M+H]^+^ (magenta) and their XICs of commercials standards are also shown, but no peaks corresponding to retention times of standard **15** or **16** were detected.


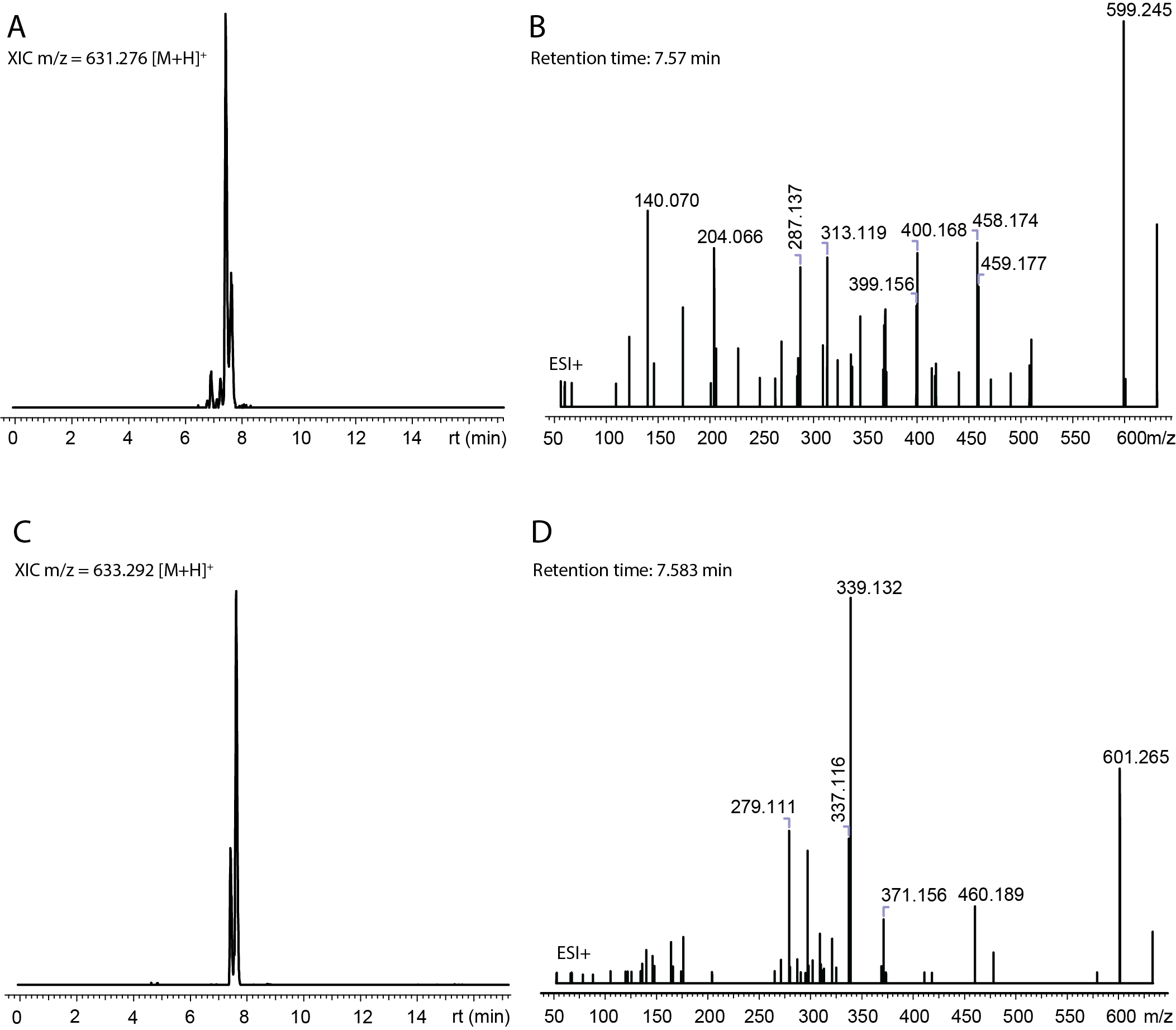


# **Figure S14** **XIC traces for *Vv*-DPleB-51 8 and *Vv*-DPxB-63 9 detected in lysate of yellow-green grapevine leaves by HR-MS^2^.**

**A** XIC trace for Vv-DPxB-63 **9** at m/z = 631.276 [M+H]^+^. **B** HR-MS^2^ for Vv-DPxB-63 **9**. **C** XIC trace for detected *Vv*-DPleB-51 **8** at m/z = 633.292 [M+H]^+^. **D** HR-MS^2^ for detected *V*v-DPleB-51 **8**.


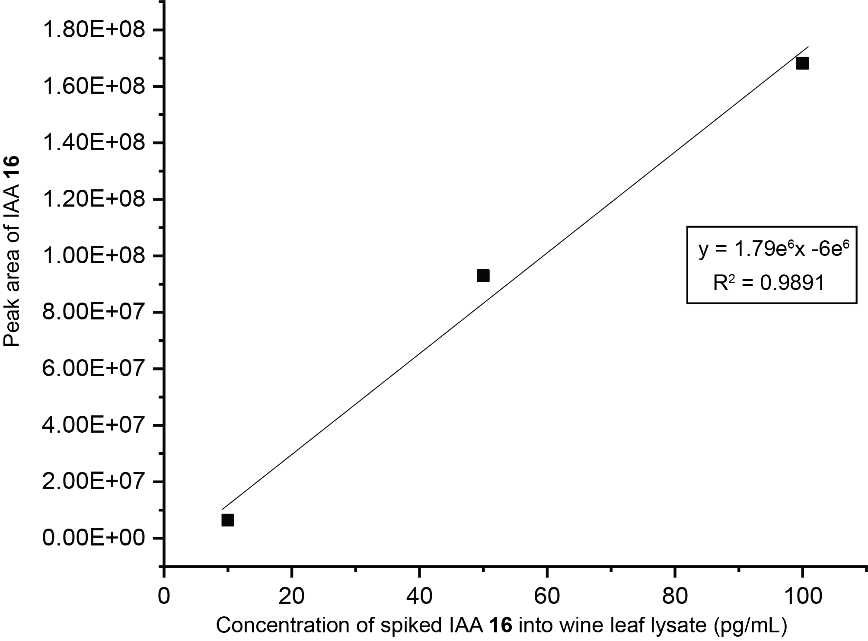


# **Figure S15 IAA 16 spiked into grapevine leaf lysate to determine detection limit with HR-MS Orbitrap.**

10 pg/mL was the lowest concentration to detect a peak and calculate an area under the curve (AUC) by XIC. **Box:** Linear fitted curve for the measured concentrations of IAA spiked into grapevine leaf lysate.


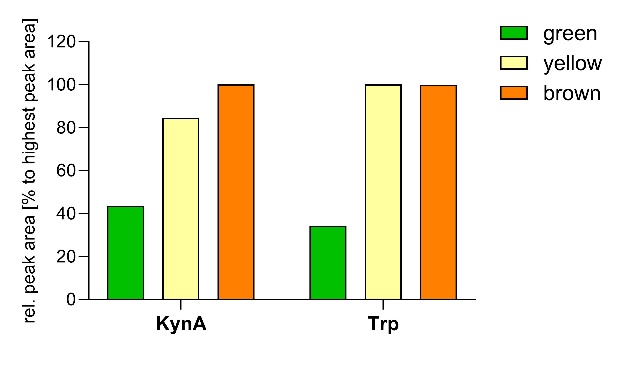

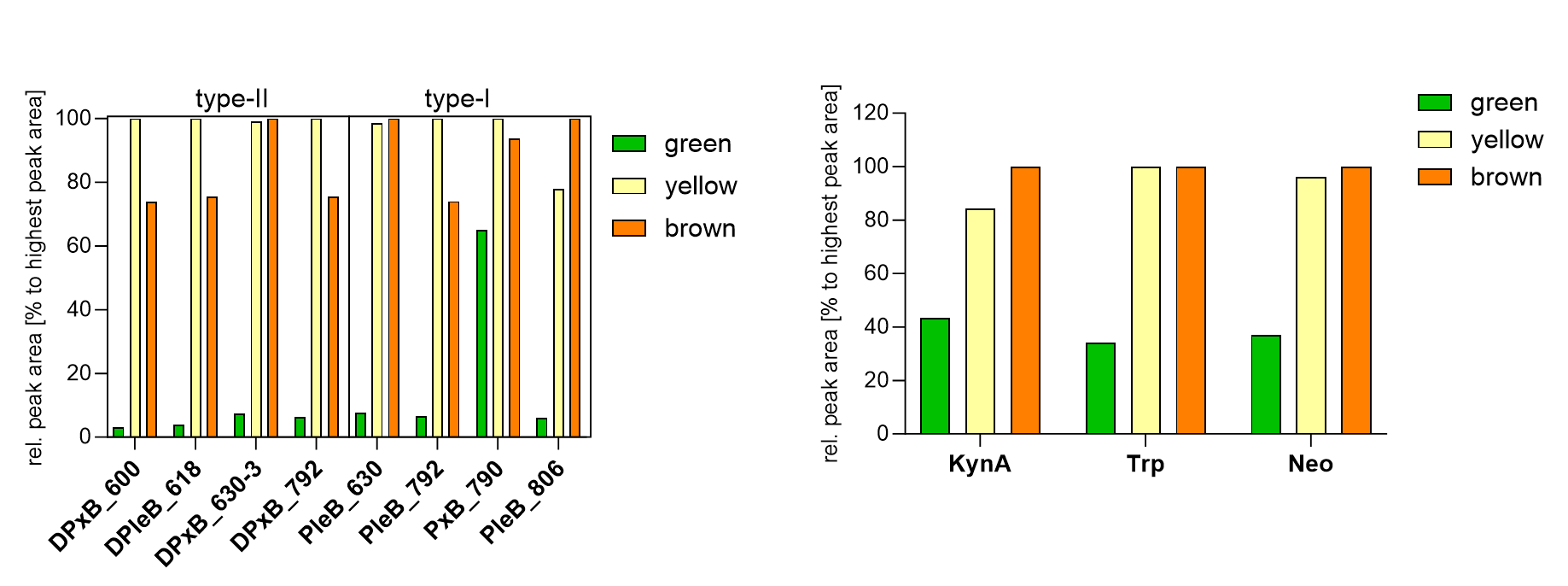


# **Figure S16 Relative phyllobilin and tryptophan metabolite profiles in green, yellow, and brown *A. thaliana* siliques.**

**Left**: Relative peak areas of type-I and type-II phyllobilins detected in extracts from green, yellow, and brown siliques. Each bar represents the normalized peak area (percentage of the highest peak within each compound type). Phyllobilin structures were assigned based on literature data and confirmed by MS² fragmentation patterns.^69^ **Right**: Relative abundances of kynurenic acid (KynA) and tryptophan (Trp), in the same color groups.

**
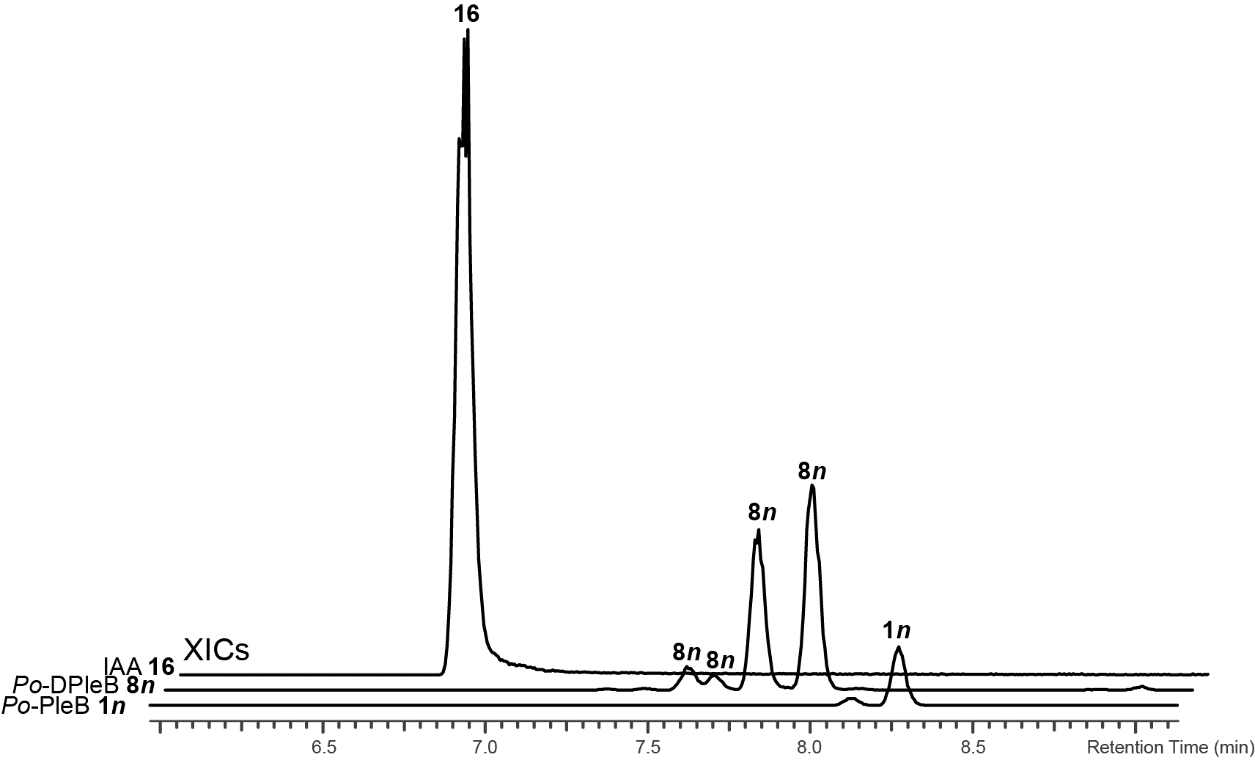
**

# **Figure S17** **Inhibition test of *Po*-PleB 1*n* conversion with a 10-fold excess of IAA 16 towards 1*n*.**

HR-MS XIC traces for *Po*-DPleB **8*n*** formation by *Vv*YUCCA10-MBP incubated with a 10-fold excess of IAA **16** towards the substrate **1*n*** are shown.

**
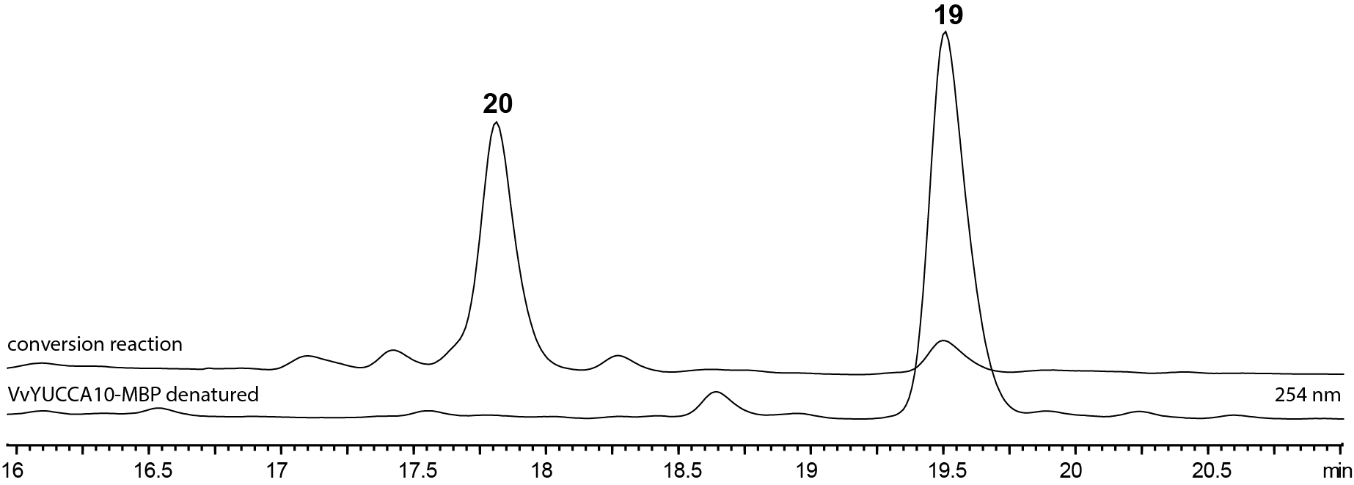
**

# **Figure S18** **HPLC-DAD chromatogram shown at 254 nm of control reactions for conversion reaction of *Cj*-PleB-2 19 to its deformylated version 20 with denatured enzyme *Vv*YUCCA10-MBP.**

**
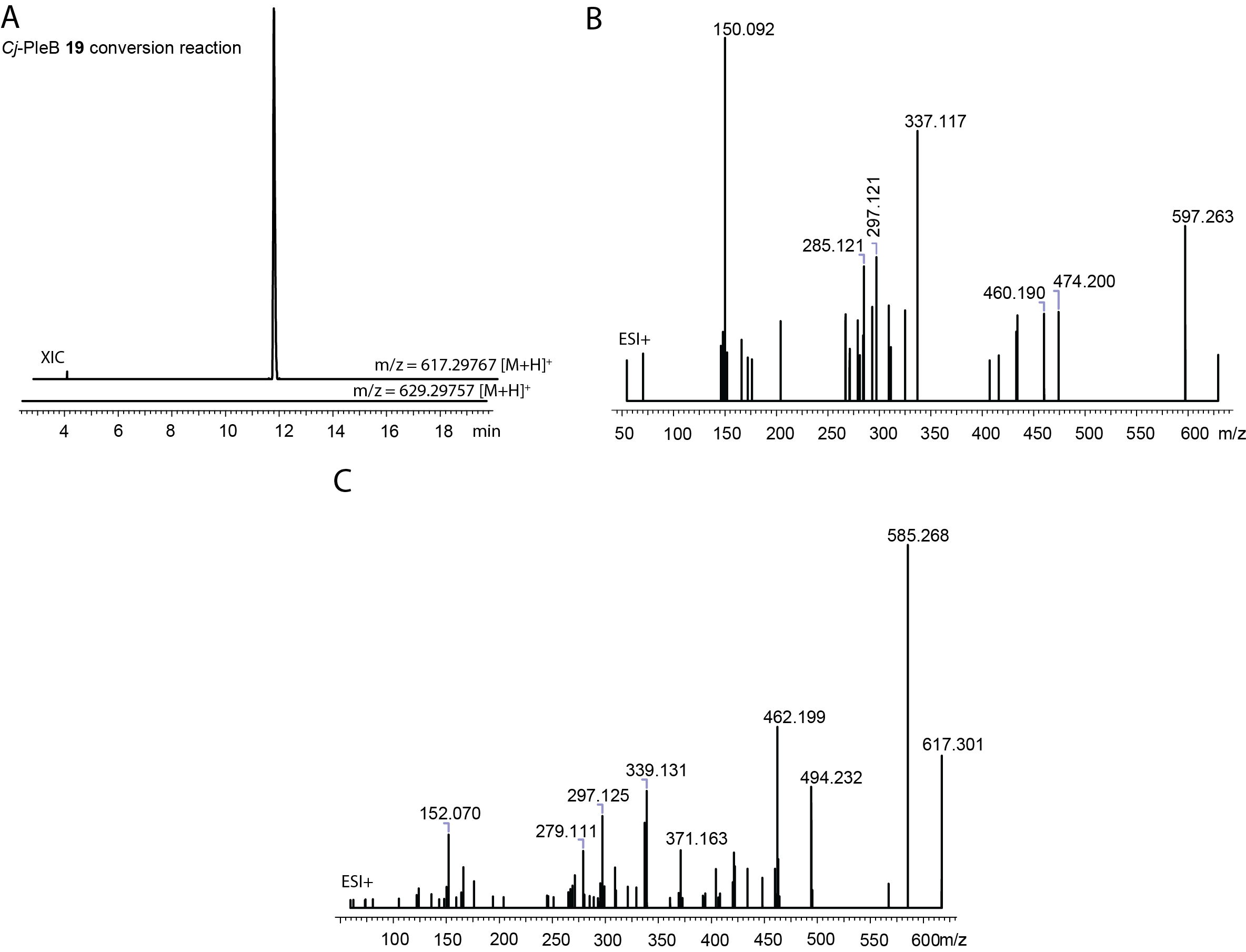
**

# **Figure S19 *Cj*-PleB-2 19 conversion assays with NADPH and *Vv*YUCCA10-MBP.**

**A** XIC traces for substrate *Cj*-PleB-2 **19** (m/z = 629.29757 [M+H]^+^) and product **20** (m/z = 617.29757 [M+H]^+^) are shown from UHPLC-HR-MS measurement in positive ion mode. **B** MS^2^ HR fragments of *Cj*-PleB-2 **19**. **C** MS^2^ HR fragments of product **20**.

**
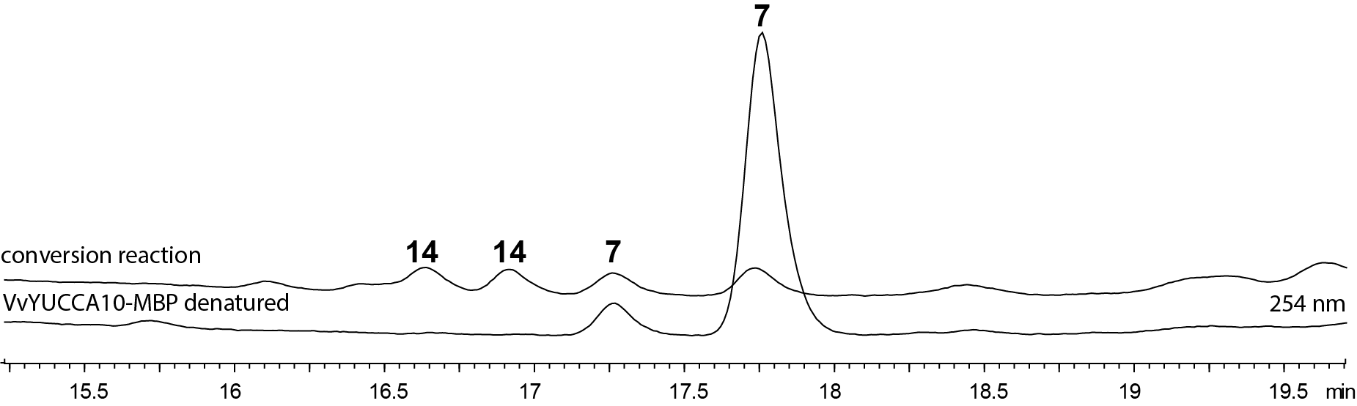
**

# **Figure S20** **HPLC-DAD chromatogram shown at 254 nm of control reactions for conversion reaction of *Cj*-PrB 7 to deformylated DPrB 14 with denatured enzyme *Vv*YUCCA10-MBP shown at 254 nm.**


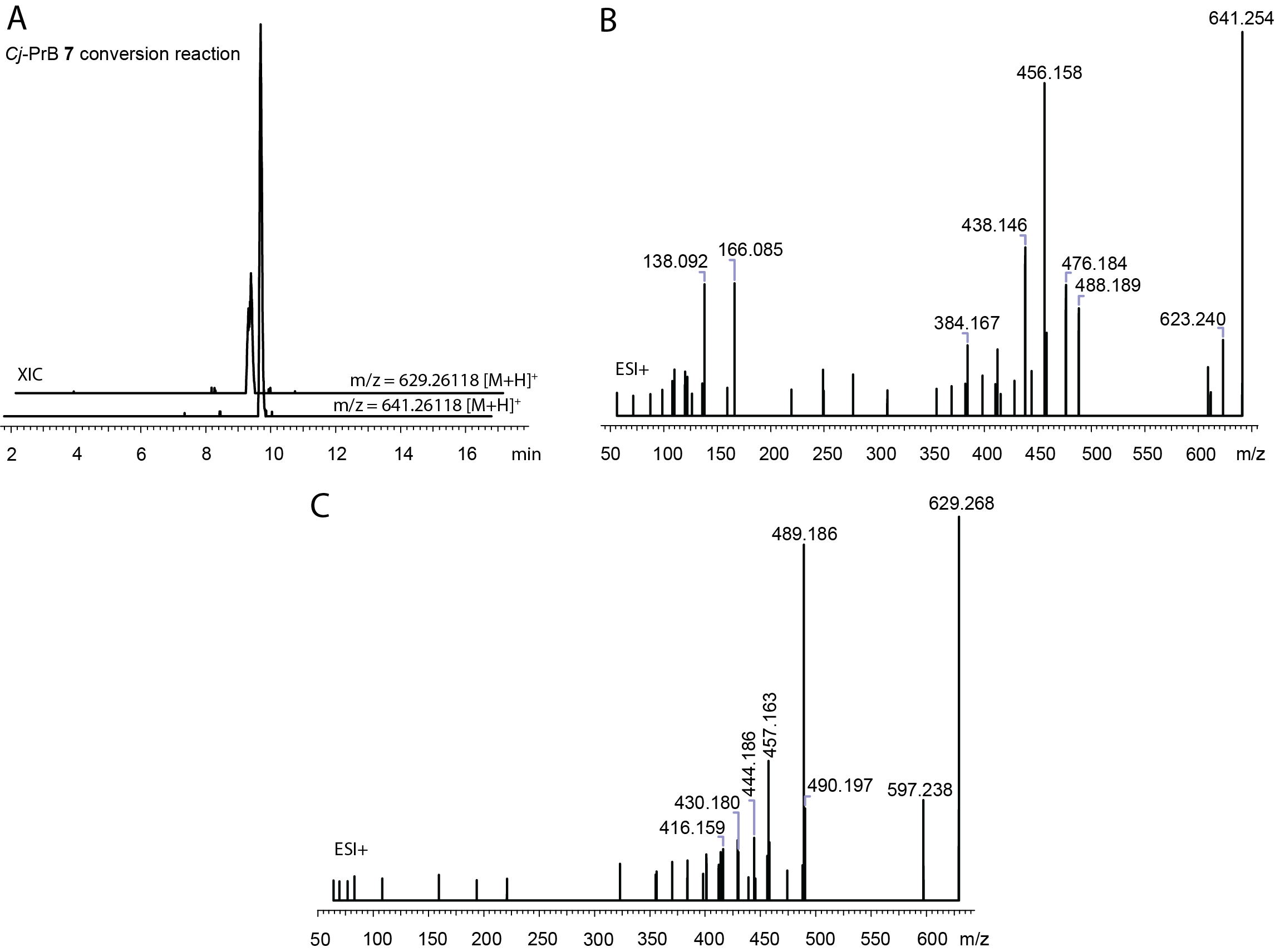


# **Figure S21** ***Cj*-PrB 7 conversion assays with NADPH and *Vv*YUCCA10-MBP.**

**A** XIC traces for *Cj*-PrB **7** (m/z = 641.26118 [M+H]^+^) and DPrB **14** (m/z = 629.26118 [M+H]^+^) are shown from UHPLC-HR-MS measurement in positive ion mode. **B** MS^2^ HR fragments of *Cj*-PrB **7**. **C** MS^2^ HR fragments of DPrB **14**.

**
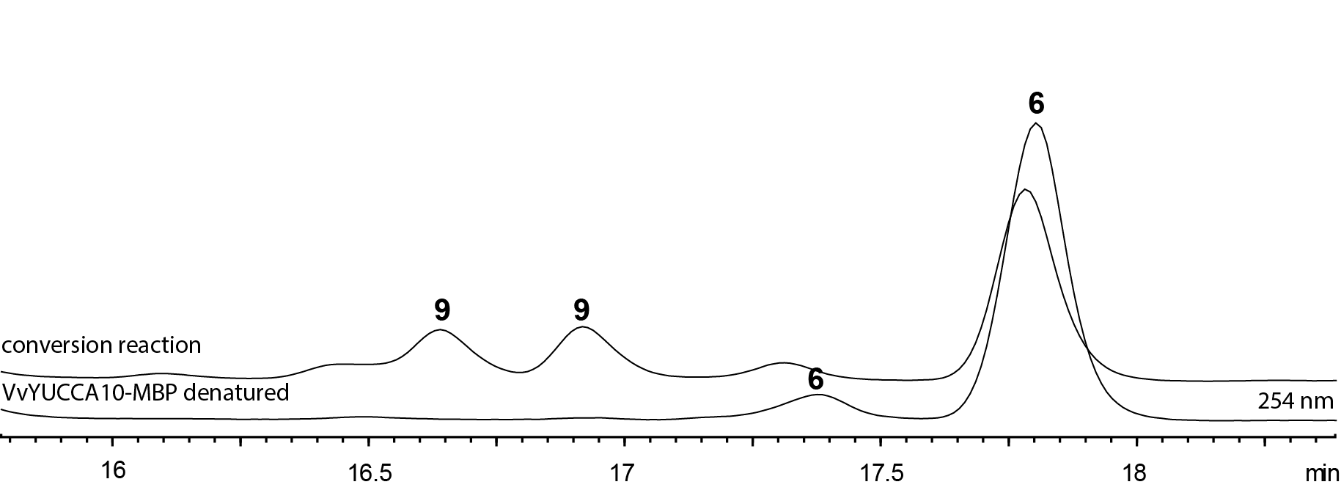
**

# **Figure S22** **HPLC-DAD chromatogram shown at 254 nm of control reactions for conversion reaction of *Cj*-PxB 6 to deformylated *Vv*-DPxB-63 9 conversion assay with denatured enzyme *Vv*YUCCA10-MBP shown at 254nm.**


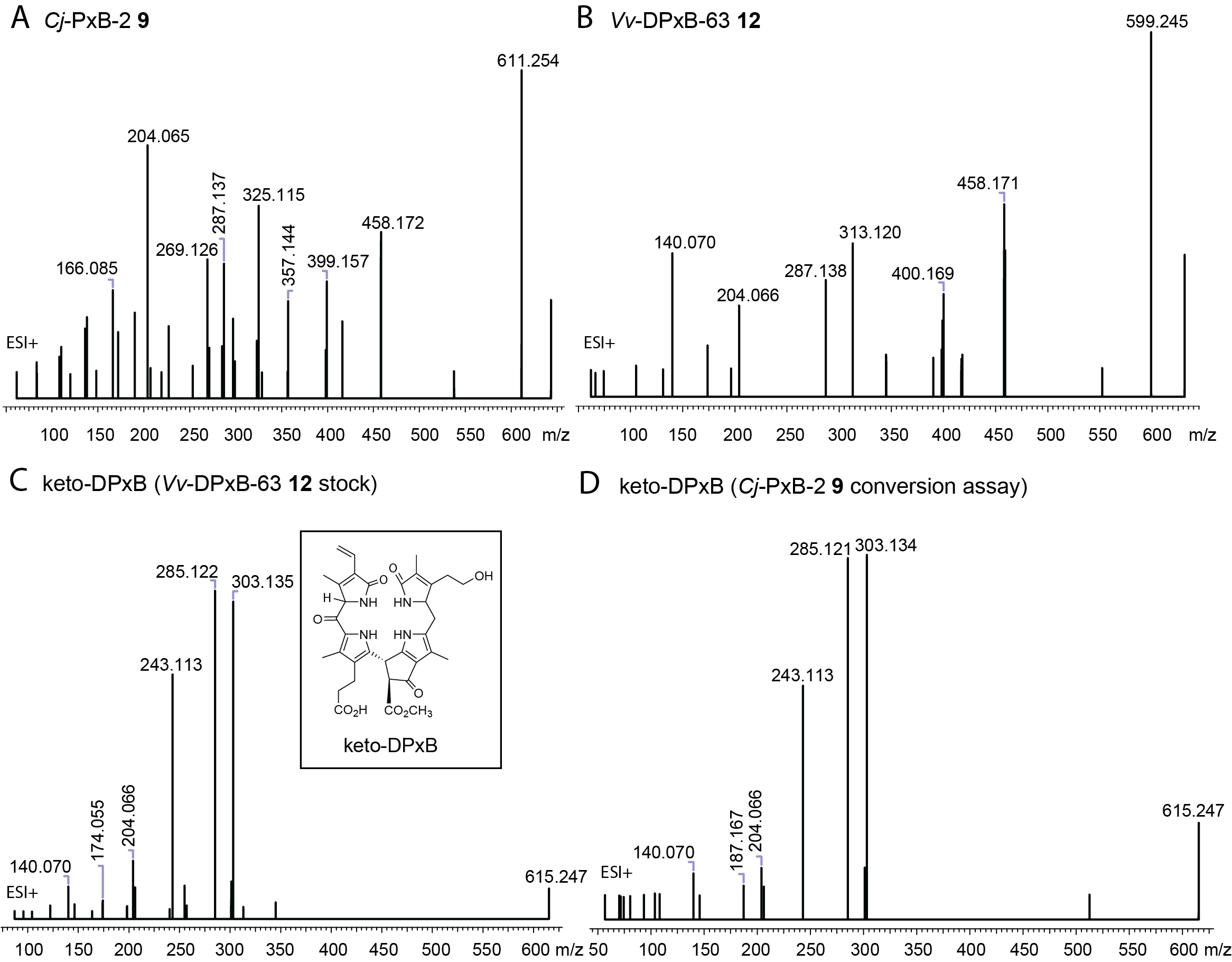


# **Figure S23** ***Cj*-PxB-2 6 conversion assays with NADPH and *Vv*YUCCA10-MBP.**

**A** MS^2^ HR fragments for *Cj*-PxB-2 **6** (m/z = 643.27684 [M+H]^+^). **B** MS^2^ HR fragments of *Vv*-DPxB-63 **9** (m/z = 631.27684 [M+H]^+^). **C** MS^2^ HR fragments of a presumed ‘keto*-*DPxB’ found in *Vv*-DPxB-63 **9** stock. **D** MS^2^ HR fragments of ‘keto*-*DPxB’ produced by *Vv*YUCCA10-MBP. All measurements were conducted in positive ion mode.


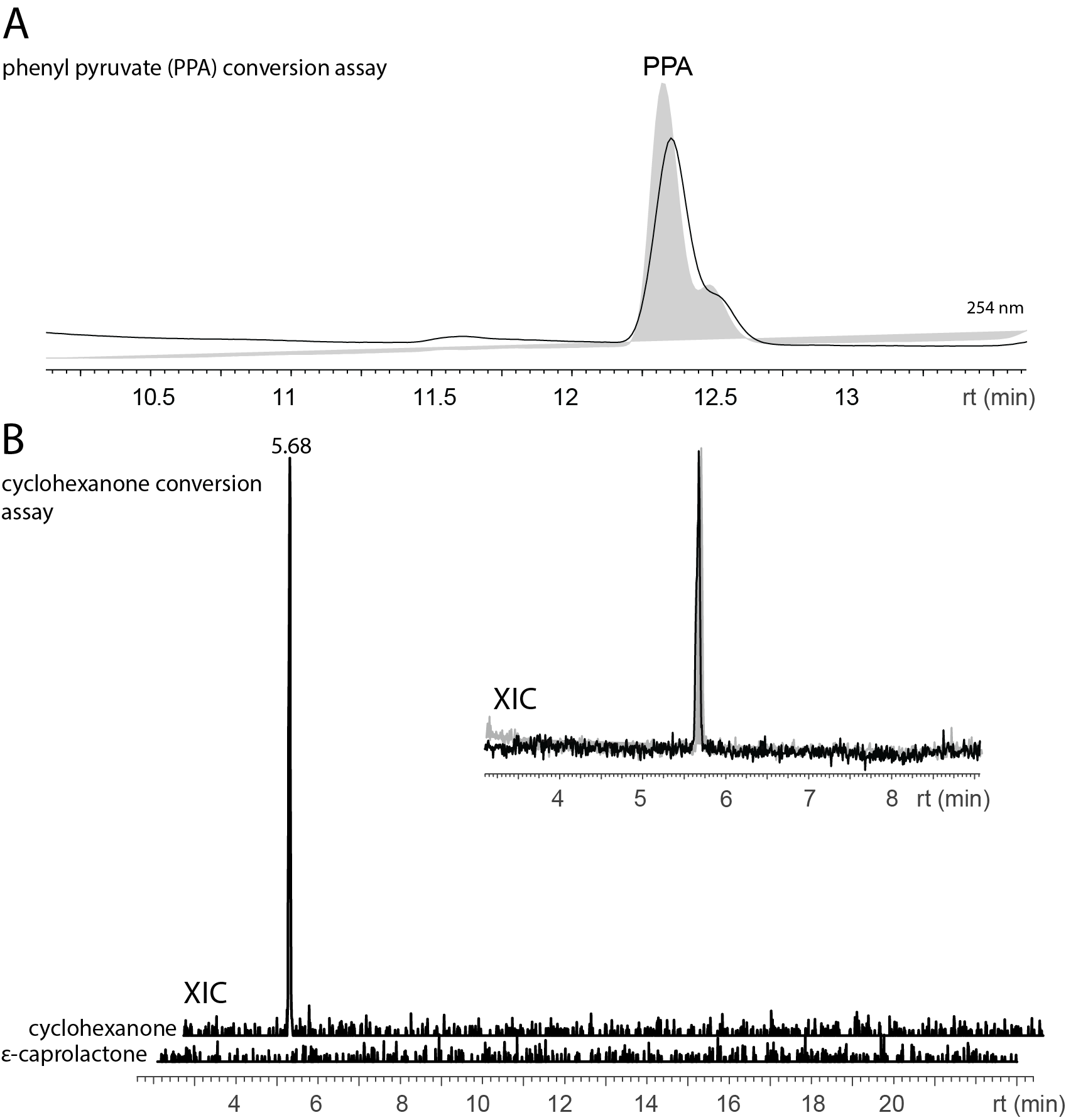


# **Figure S24 Results of conversion assays with cyclohexanone and phenyl pyruvate as substrate for *Vv*YUCCA10.**

**A** HPLC-DAD chromatogram recorded at 254 nm shown for the phenyl pyruvate (PPA) conversion assay with *Vv*YUCCA10-MBP. The grey shadow shows a PPA standard measured with the same method and equalized y-axis. **B** GC-MS chromatogram shown for the cyclohexanone conversion assay with *Vv*YUCCA10-MBP. XIC traces selected for cyclohexanone (rt 5.68 min, m/z = 98) and ε-caprolactone (m/z = 114) are shown for EI^+^ mode. The grey shadow in the zoomed inset figure shows a cyclohexanone standard measured with the same method and equalized y-axis.


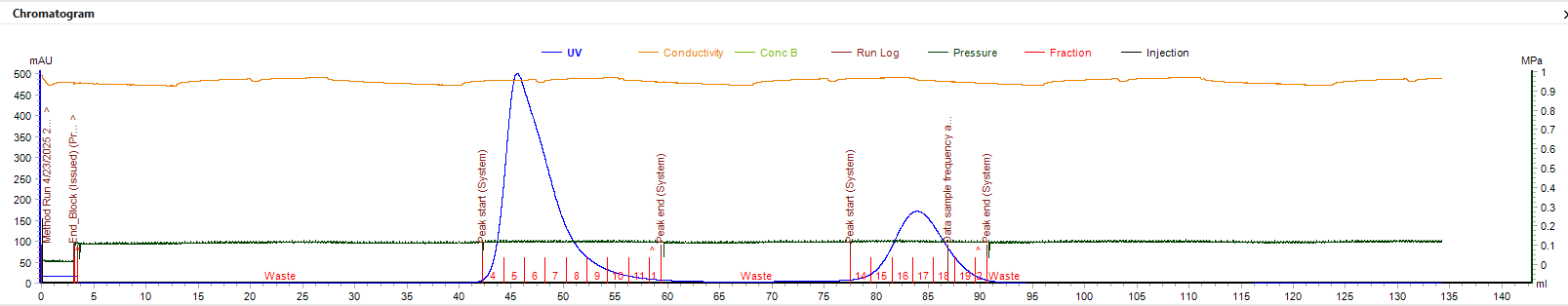
A

**
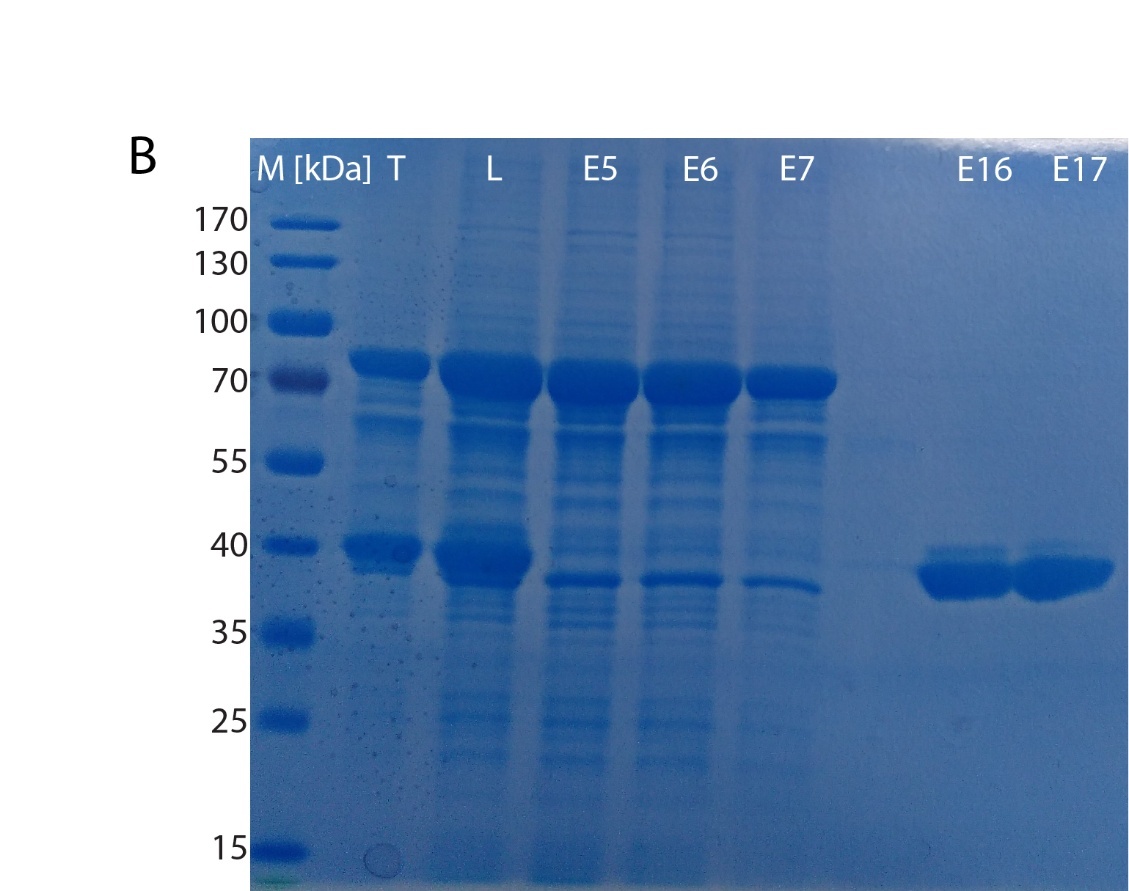
**

# **Figure S25 Chromatogram and SDS-gel analysis of size exclusion chromatography (SEC) of MBP-tagged *Vv*YUCCA10.**

*Vv*YUCCA10-MBP has a molecular weight of 86.5 kDa. **A** Size exclusion chromatography (SEC) chromatogram showing that *Vv*YUCCA10-MBP elutes as aggregate peak E4-E12. E14-E20 contain eluted MBP-tag. **B** SDS-PAGE analysis of different fractions collected during SEC of MBP-tagged *Vv*YUCCA10. M: Marker, PageRuler (Prestained protein ladder, ThermoScientific), T: Elution peak MBP-trap, L: Loaded protein onto SEC column, E5-7: Eluted fractions from SEC chromatogram containing VvYUCCA10-MBP inside, E16 and E17: Eluted fractions from SEC chromatogram with MBP-tag inside.


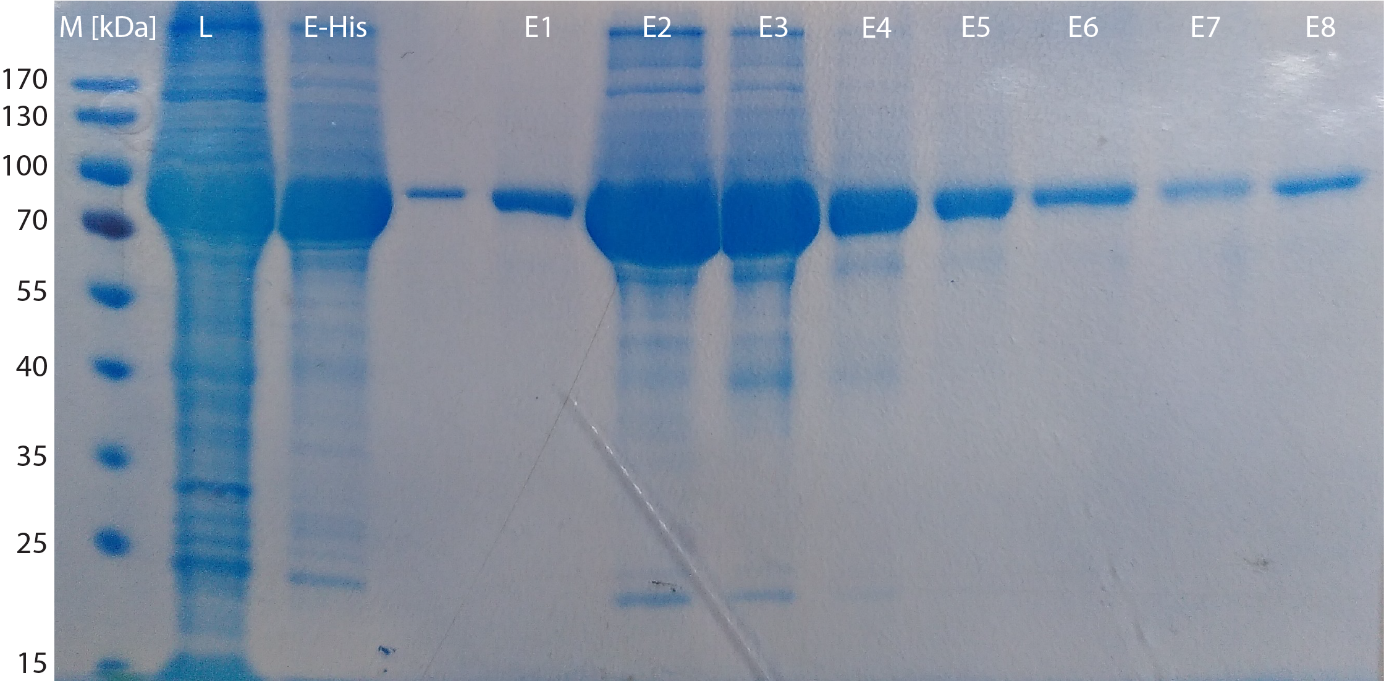


# **Figure S26 SDS-PAGE analysis of different fractions collected during affinity purification (IMAC) of MBP-tagged *At*YUCCA10.**

*At*YUCCA10-MBP has a molecular weight of 85 kDa. M: Marker, PageRuler (Prestained protein ladder, ThermoScientific), L: cleared *E. coli* lysate. E-His: Fraction containing *At*YUCCA10-MBP after His-trap purification. E1-E8: Elution fractions 1-8 during MBP-trap purification containing *At*YUCCA10-MBP.


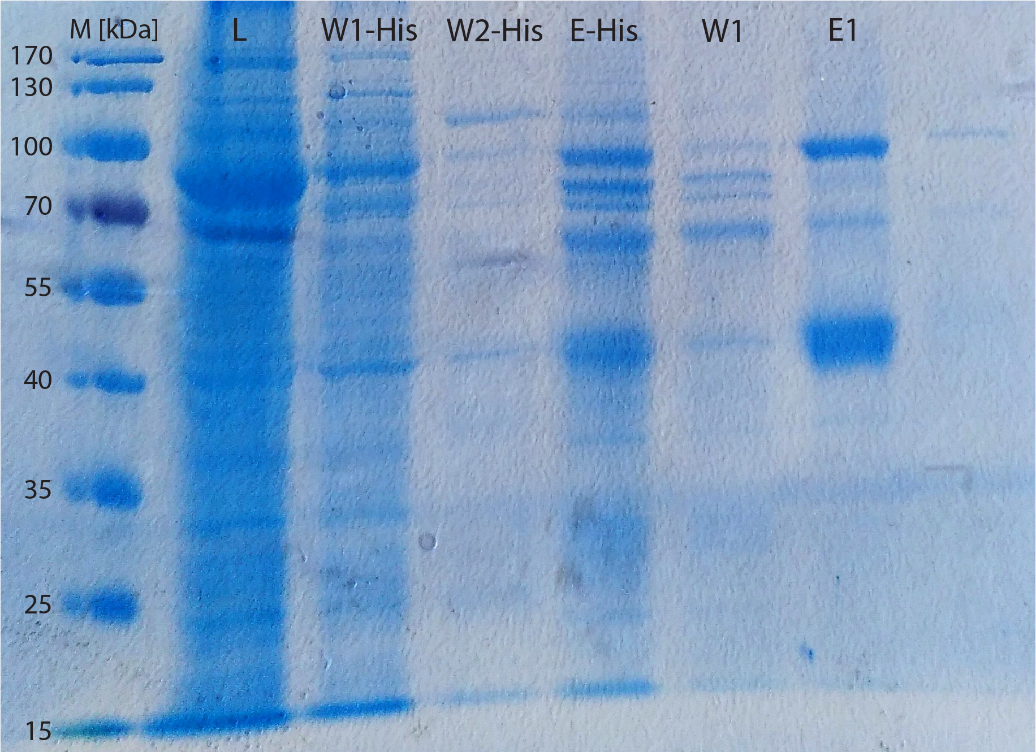


# **Figure S27 SDS-PAGE analysis of different fractions collected during affinity purification (IMAC) of MBP-tagged *Ca*YUCCA10.**

*Ca*YUCCA10-MBP has a molecular weight of 85.2 kDa. M: Marker, PageRuler (Prestained protein ladder, ThermoScientific), L: Cleared cell lysate, W1-His and W2-His: Wash fractions from His-trap purification, E-His: Fraction containing *Ca*YUCCA10-MBP after His-trap purification, W1: Wash fraction from MBP-trap purification, E1: Elution fraction from MBP-trap purification containing *Ca*YUCCA10-MBP.


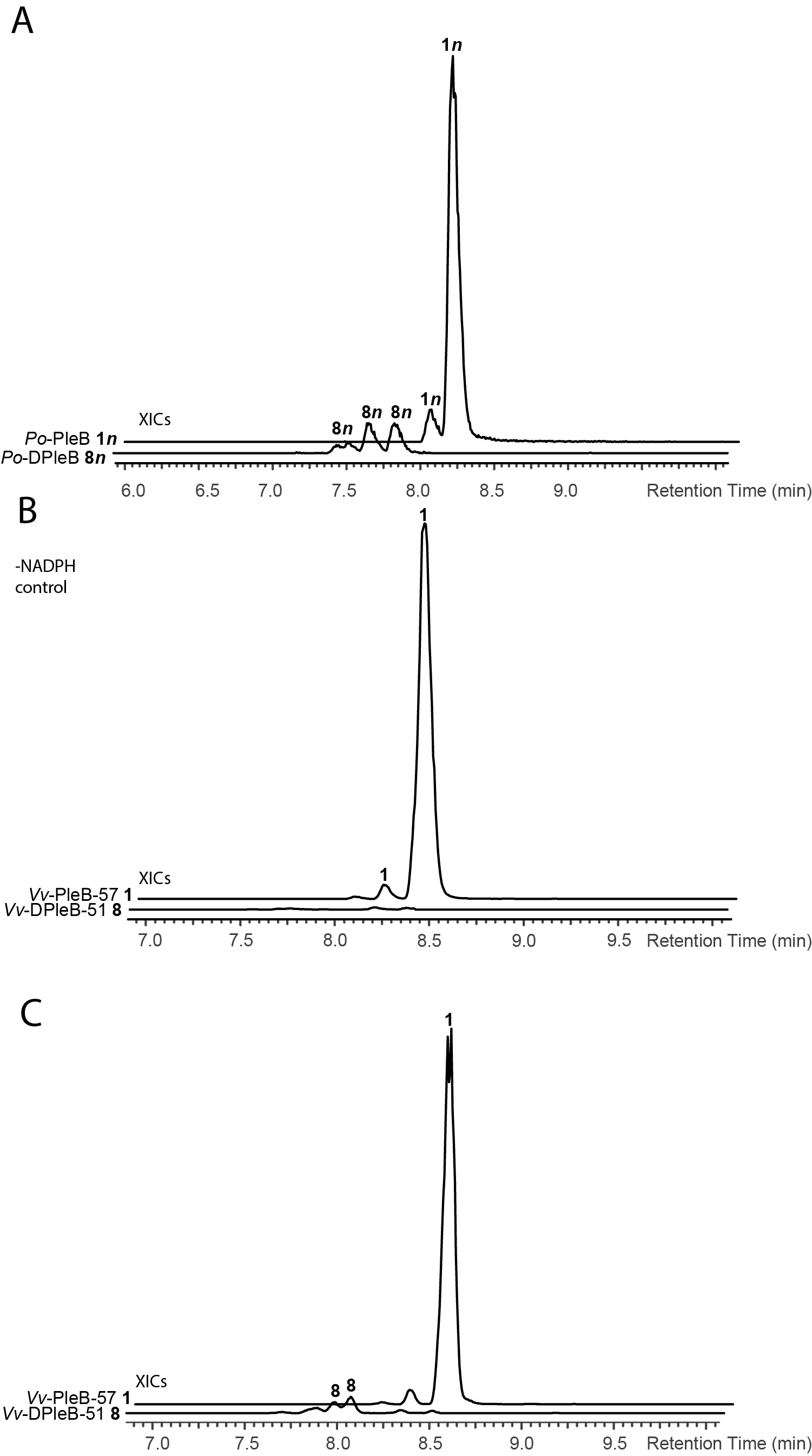


# **Figure S28** **Control reactions for *Vv*-PleB-57 1 and *Po*-PleB 1*n* conversion to *Vv*-DPleB-51 8 and *Po*-DPleB 8*n* catalyzed by *Ca*YUCCA10-MBP.**

All peaks indicated for **8** and **8*n*** share the same mass and fragments and are assumed stereoisomers of epimers **1** and **1*n***. The epimerization of **1** and **1*n*** is commonly observed with acetonitrile as a LC solvent. The reaction to **8**/**8*n*** creates a new chiral center that leads to further isomerization of the two epimers into four peaks. **A** Conversion reaction of *Po*-PleB **1*n*** (m/z = 645.29249 [M+H]^+^) to **8*n*** (m/z = 633.29249 [M+H]^+^) catalyzed by *Ca*YUCCA10-MBP shown as XICs. **B** XICs of conversion reaction of *Vv*-PleB-57 **1** to **8** catalyzed by *Ca*YUCCA10-MBP without added cofactor NADPH measured with HR-MS. **C** XICs of conversion reaction of *Vv*-PleB-57 **1** to **8** catalyzed by *Ca*YUCCA10-MBP measured with HR-MS. The y-axis scale was equalized by setting the highest peak to 100% for all measurements.

**
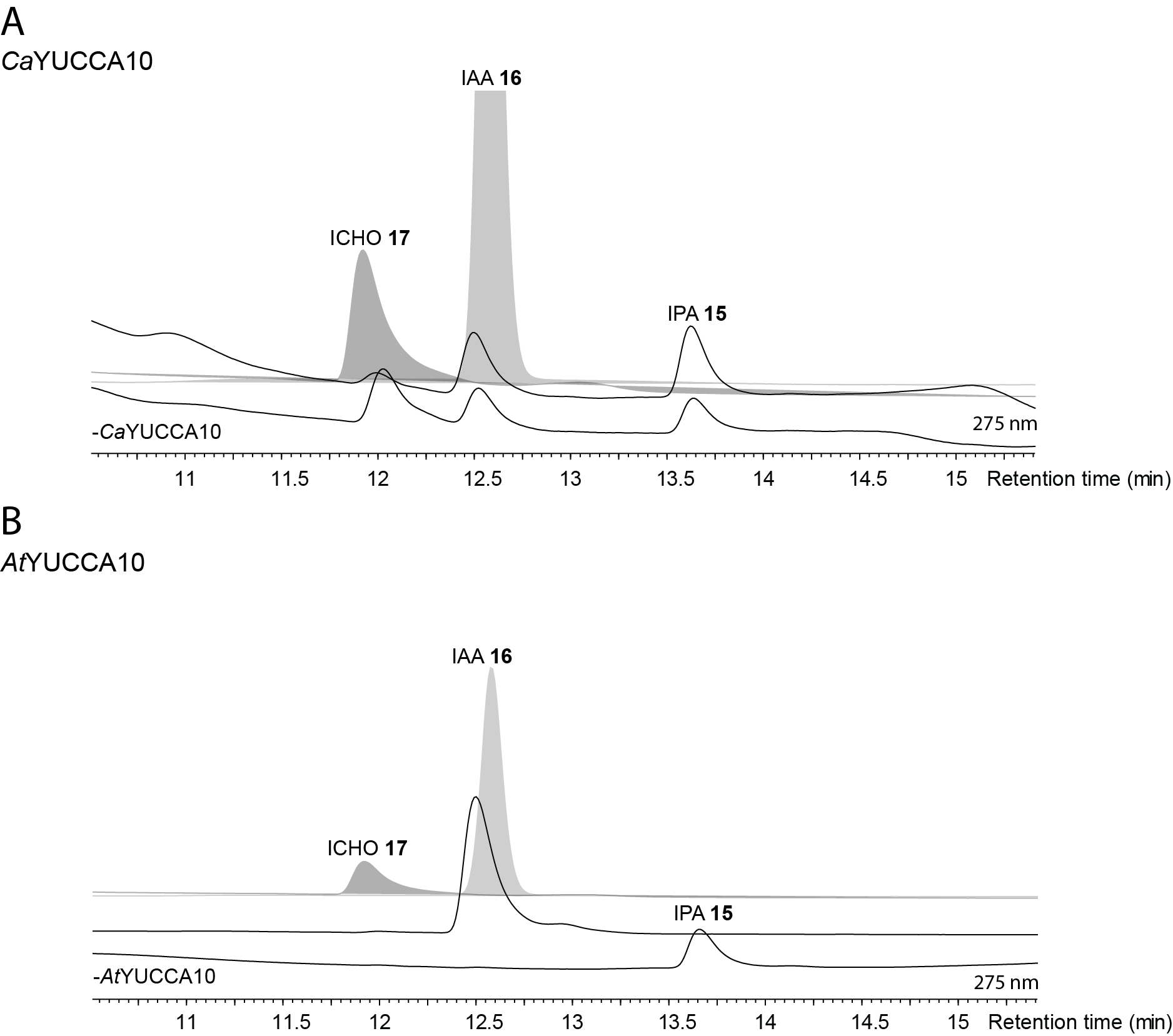
**

# **Figure S29** **IPA 15 conversion to IAA 16 catalyzed by *Ca*YUCCA10-MBP and *At*YUCCA10-MBP.**

**A** HPLC-DAD chromatogram shown at 275 nm of the conversion reaction catalyzed by *Ca*YUCCA10-MBP with IPA **16** after 20 min at 30°C and 600 rpm. The lower trace shows a negative control without added enzyme. Standards of IAA **16** and ICHO **17** are indicated by their shadow. **B** HPLC-DAD chromatogram shown at 275 nm of the conversion reaction catalyzed by *At*YUCCA10-MBP with IPA **16** after 30 min at 30°C and 600 rpm. The lower trace shows a negative control without added enzyme. Standards of IAA **16** and ICHO **17** are indicated by their shadow.


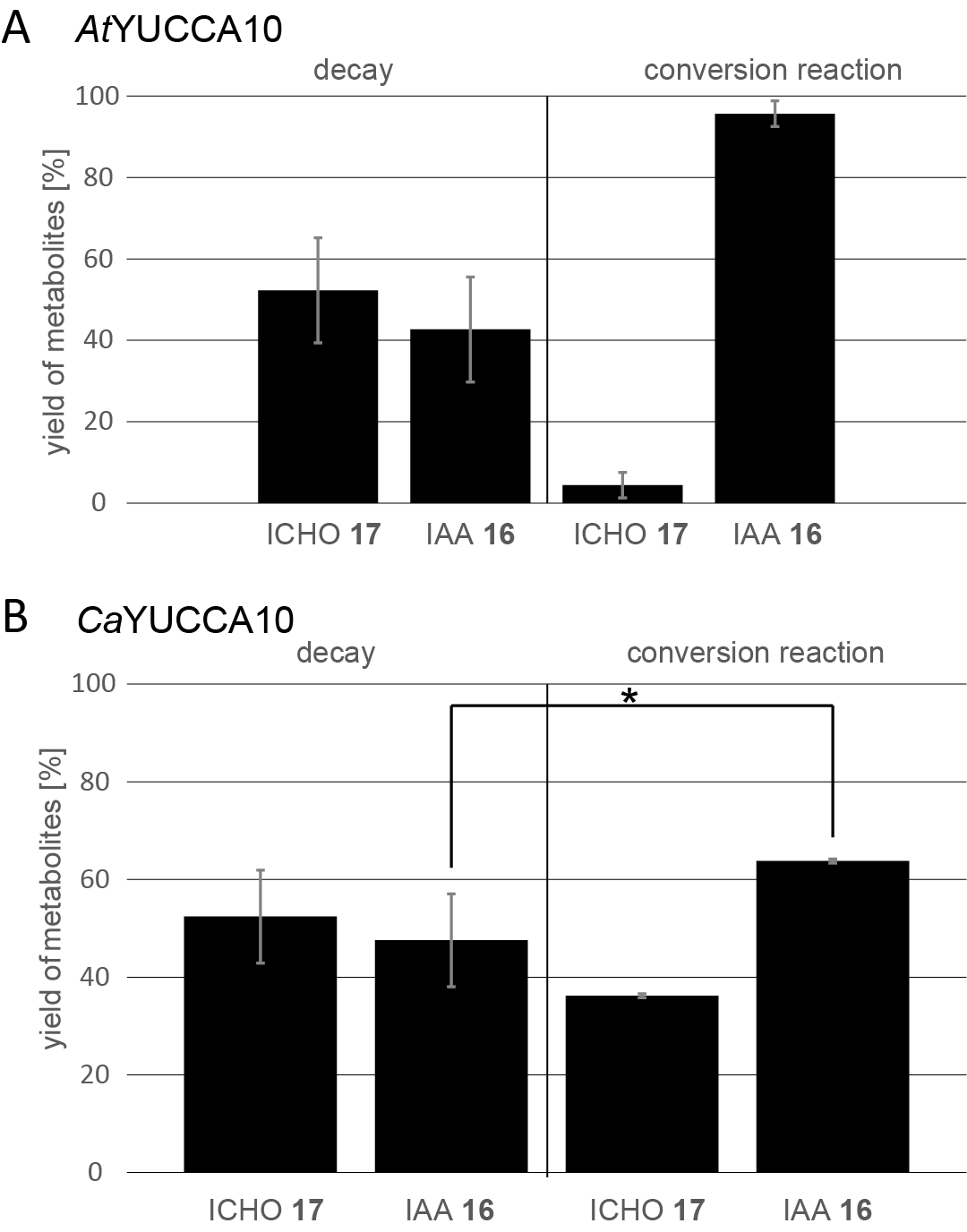


# **Figure S30 Bar chart comparing IPA 15 decay to enzymatic reaction catalyzed by *At*YUCCA10-MBP and *Ca*YUCCA10-MBP.**

**A** The bar chart shows the mean product yields of triplicates given in percent of IPA **15** decay compared to ICHO **17** and IAA **16** produced by enzymatic reaction catalyzed by *At*YUCCA10-MBP. **B** The bar chart shows the mean product yields of triplicates given in percent of IPA **15** decay compared to ICHO **17** and IAA **16** produced by enzymatic reaction catalyzed by *Ca*YUCCA10-MBP. Statistics were assessed by a two-sample t-test with equal variance assumption for triplicates. The product yields were determined by area under the curves (AUC) of XIC traces generated for **16** and **17** and subsequent calculation of product formation in percent as ratio of **16** to overall **16** and **17** formation. The error bars are shown as calculated standard deviations from the mean of 3 replicates. Statistical analysis resulted in a significant increase (P = 0.0418) of IAA **16** production for *Ca*YUCCA10. Values that were significantly different were marked by bars with ****: P<0.0001.***: P<0.0001-0.001, **:P<0.001-0.01 and *:P<0.01-0.05.


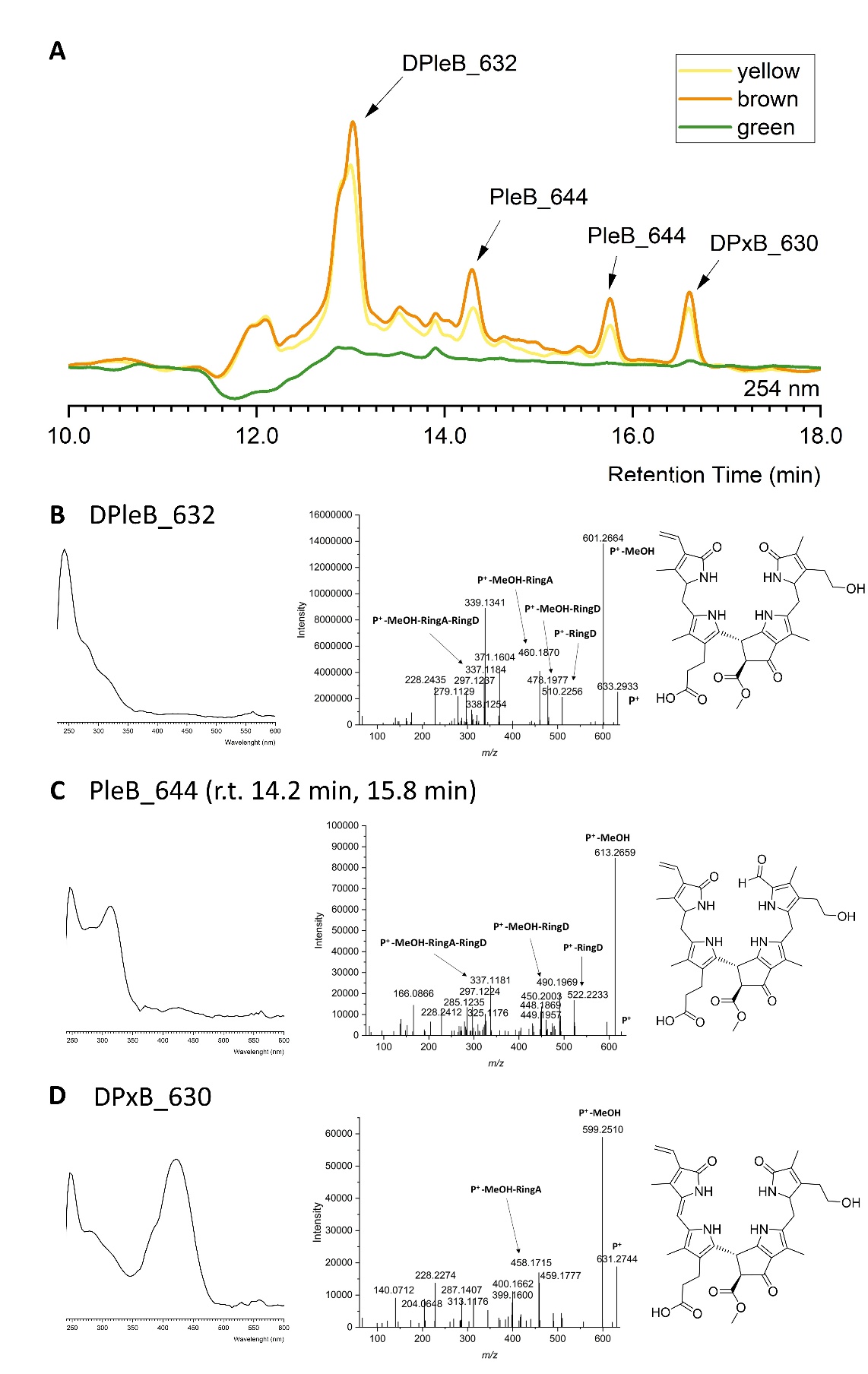


# **Figure S31** **HPLC-DAD chromatogram shown at 254 nm of lysate of coffee leaves and extracted ion chromatograms (XIC) shown for chlorophyll (Chl) catabolites detected.**

**A** HPLC-DAD chromatogram shown at 254 nm of extracts of green, yellow and brown leaf extracts of *Coffea arabica*. Online UV spectrum, MS^2^ spectrum and chemical structure of DPleB (**B**), PleB (**C**) and DPxB (**D**).

# **Figure S32** **Relative phyllobilin profiles in green, yellow, and brown *Coffea arabica* leaves.**

Relative peak areas of type-I and type-II phyllobilins detected in extracts from green, yellow, and brown leaves. Each bar represents the normalized peak area (percentage of the highest peak within each compound type). Phyllobilin structures were assigned based on characteristic UV spectra and MS² fragmentation patterns as seen in Figure S31.


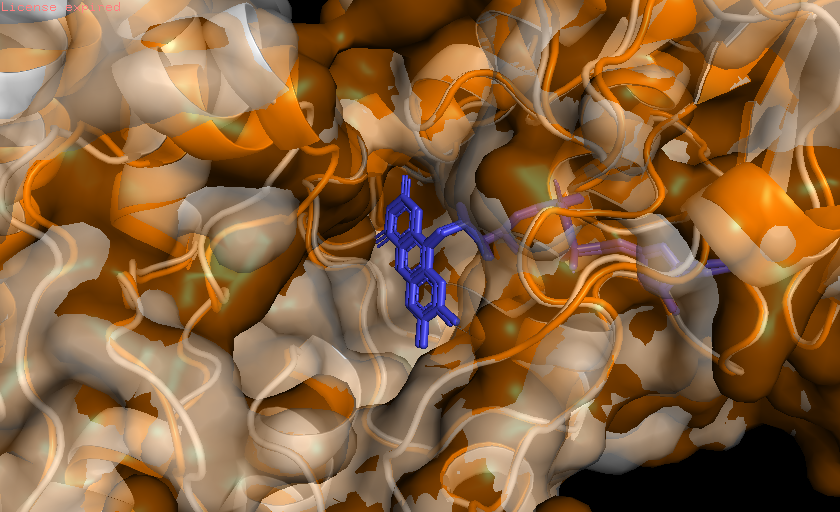


# **Figure S33** **Alphafold model prediction of *Vv*YUCCA10 and *Ca*YUCCA10 with co-factor FAD (blue).**

The overlay was generated by alignment with PyMOL. The view into the active site is shown.


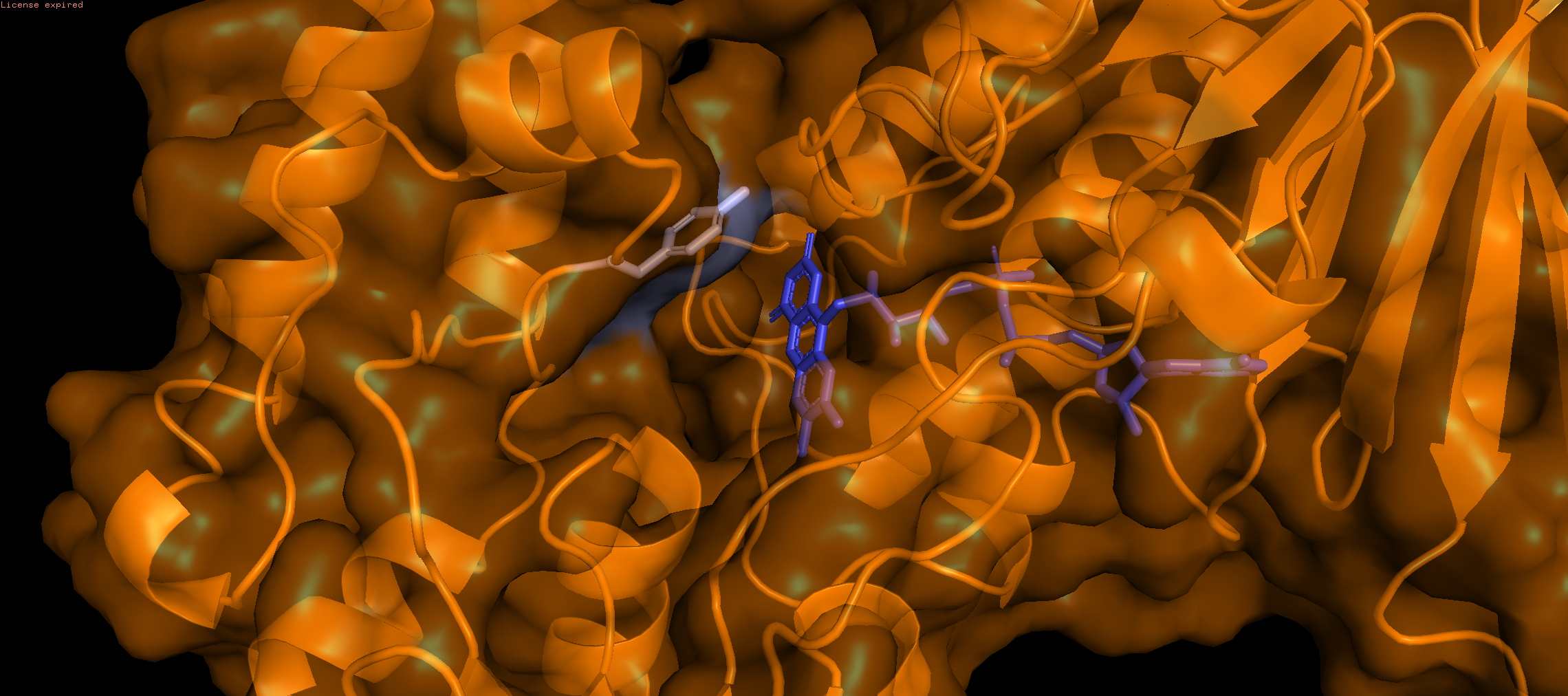


# **Figure S34** **Alphafold model prediction of *Vv*YUCCA10 with putative Tyr as catalytic residue.**

Tyr266 highlighted in beige, which is absent in *At*YUCCA10.


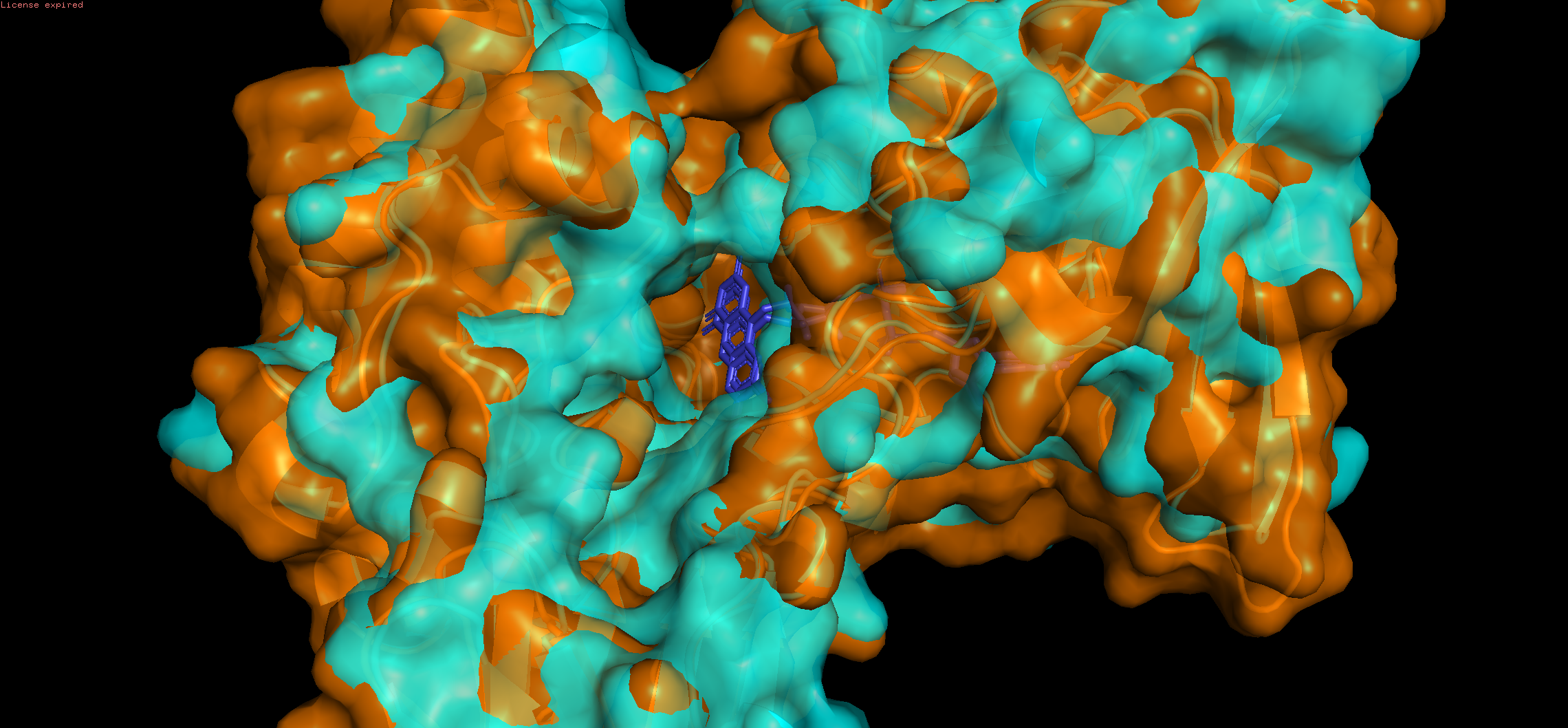


# **Figure S35** **Alphafold model prediction of *Vv*YUCCA10 and *At*YUCCA10 with co-factor FAD (blue).**

The overlay was generated by alignment with PyMOL. The view into the active site is shown.

# **SI References**

[5] C. A. Karg, M. Taniguchi, J. S. Lindsey, S. Moser “Phyllobilins – Bioactive Natural Products Derived from Chlorophyll – Plant Origins, Structures, Absorption Spectra, and Biomedical Properties.” *Planta Med.* **2023**, 89, 637.

[7] M. Oberhuber, J. Berghold, K. Breuker, S. Hörtensteiner, B. Kräutler “Breakdown of chlorophyll: A nonenzymatic reaction accounts for the formation of the colorless “nonfluorescent” chlorophyll catabolites.” *Proc. Natl. Acad. Sci. U.S.A.* **2003**, 100, 6910.

[11] C. A. Karg, P. Wang, F. Kluibenschedl, T. Müller, L. Allmendinger, A. M. Vollmar, S. Moser “Phylloxanthobilins are Abundant Linear Tetrapyrroles from Chlorophyll Breakdown with Activities Against Cancer Cells.” *Eur. J. Org. Chem.* **2020**, 4499.

[16] T. Erhart, C. Mittelberger, X. Liu, M Podewitz, C. Li, G. Scherzer, G. Stoll, J. Valls, P. Robatscher, K. R. Liedl, M. Oberhuber, B. Kräutler “Novel Types of Hypermodified Fluorescent Phyllobilins from Breakdown of Chlorophyll in Senescent Leaves of Grapevine ( *Vitis vinifera* ).” *Chem. Eur. J.* **2018**, 24, 17268.

[32] V. Kriechbaumer, S. W. Botchwy, C. Hawes “Localization and interactions between Arabidopsis auxin biosynthetic enzymes in the TAA/YUC-dependent pathway.” *J. Exp. Bot.* **2016**, 67, 4195.

[40] T. Obayashi, H. Hibara, Y. Kagaya, Y. Aoki, K. Kinoshita “ATTED-II v11: A Plant Gene Coexpression Database Using a Sample Balancing Technique by Subagging of Principal Components.” *PCP* **2022**, 63, 869.

[43] L. Kuang, S. Chen, Y. Guo, H. Ma “Quantitative Proteome Analysis Reveals Changes in the Protein Landscape During Grape Berry Development With a Focus on Vacuolar Transport Proteins.” *Front. Plant Sci.* **2019**, 10, 641.

[49] Y. Yamamoto, N. Kamiya, Y. Morinaka, M. Matsuoka, T. Sazuka “Auxin Biosynthesis by the *YUCCA* Genes in Rice.” *Plant Physiol.* **2007**, 143, 1362.

[53] S. Moser, M. Ulrich, T. Müller, B. Kräutler “A yellow chlorophyll catabolite is a pigment of the fall colours.” *Photochem. Photobiol. Sci.* **2008**, 7, 1577.

[57] M. Ulrich, S. Moser, T. Müller, B. Kräutler “How the Colourless ‘Nonfluorescent’ Chlorophyll Catabolites Rust.” *Chem. Eur. J.* **2011**, 17, 2330.

[63] S. Lu, M. Zhang, Y. Zhuge, W. Fu, Q. Quyang, W. Wang, Y. Ren, D. Pei, J. Fang “VvERF17 mediates chlorophyll degradation by transcriptional activation of chlorophyll catabolic genes in grape berry skin.” *Environ. Exp. Bot.* **2022**, 193, 104678.

[64] J. Jeong, H. Yim, J. Ryu, H. S. Lee, H. Lee, D. Seen, S. G. Kang “One-Step Sequence- and Ligation-Independent Cloning as a Rapid and Versatile Cloning Method for Functional Genomics Studies.” *Appl. Environ. Microbiol.* **2012**, 78, 5440.

[65] L. Höing, S: T. Sowa, M. Toplak, J. K. Reinhardt, R. Jakob, T. Maier, M. A. Lill, R. Teufel “Biosynthesis of the bacterial antibiotic 3,7-dihydroxytropolone through enzymatic salvaging of catabolic shunt products.” *Chem. Sci.* **2024**, 15, 7749.

[66] J. Abramson, J. Adler, J. Dunger, R. Evans, T. Green, A. Pritzel, O. Ronneberger, L. Willmore, A. J. Ballard, J. Bambrick, S. W. Bodenstein, D. A. Evans, C. Hung, M. O’Neill, D. Reiman, K. Tunyasuvunakool, Z. Wu, A. Žemgulytė, E. Arvaniti, C. Beattie, O. Bertolli, A. Bridgland, A. Cherepanov, M. Congreve, A. I. Cowen-Rivers, A. Cowie, M. Figurnov, F. B. Fuchs, H. Gladman, R. Jian, Y. A. Khan, C. M. R. Low, K. Perlin, A. Potapenko, P. Savy, S. Singh, A. Stecula, A. Thillaisundaram, C. Tong, S. Yakneen, E. D. Zhong, M. Zielinski, A. Žídek, V. Bapst, P. Kohli, M. Jaderberg, D. Hassabis, J. M. Jumper “Accurate structure prediction of biomolecular interactions with AlphaFold 3.” *Nature* **2024**, 630, 493.

[67] K. Tamura, G. Stecher, S. Kumar, F. U. Battistuzzi “MEGA11: Molecular Evolutionary Genetics Analysis Version 11.” *Mol. Biol. Evol.* **2021**, 38, 3022.

[68] S. Moser, T. Erhart, S. Neuhauser, B. Kräutler “Phyllobilins from Senescence-Associated Chlorophyll Breakdown in the Leaves of Basil (*Ocimum basilicum*) Show Increased Abundance upon Herbivore Attack.” *J. Agric. Food Chem.* **2020**, 68, 7132.

[69] B. Christ, M. Hauenstein, S. Hörtensteiner “A liquid chromatography–mass spectrometry platform for the analysis of phyllobilins, the major degradation products of chlorophyll in *Arabidopsis thaliana*.” *Plant J.* **2016**, 88, 505.

[70] C. Li, T. Erhart, X. Liu, B. Kräutler “Yellow Dioxobilin-Type Tetrapyrroles from Chlorophyll Breakdown in Higher Plants—A New Class of Colored Phyllobilins.” *Chem. Eur. J.* **2019**, 25, 4052.
